# Five immune-related genes as diagnostic markers for endometriosis and their correlation with immune infiltration

**DOI:** 10.3389/fendo.2022.1011742

**Published:** 2022-10-06

**Authors:** Yi Huang, Qiong Li, Rui Hu, Ruiyun Li, Yuan Yang

**Affiliations:** ^1^ The First Clinical Medical College, Lanzhou University, Lanzhou, China; ^2^ Department of Obstetrics and Gynecology, Minqin People’s Hospital, Minqin, China; ^3^ The Reproductive Medicine Center, The 1st Hospital of Lanzhou University, Lanzhou, China

**Keywords:** endometriosis, diagnosis, immune infiltration, CD8+, pathological molecular networks

## Abstract

Endometriosis (EMS) is a chronic disease that can cause dysmenorrhea, chronic pelvic pain, and infertility, among other symptoms. EMS diagnosis is often delayed compared to other chronic diseases, and there are currently no accurate, easily accessible, and non-invasive diagnostic tools. Therefore, it is important to elucidate the mechanism of EMS and explore potential biomarkers and diagnostic tools for its accurate diagnosis and treatment. In the present study, we comprehensively analyzed the differential expression, immune infiltration, and interactions of EMS-related genes in three *Homo sapiens* datasets. Our results identified 332 differentially expressed genes (DEGs) associated with EMS. Gene ontology analysis showed that these changes mainly focused on the positive regulation of endometrial cell proliferation, cell metabolism, and extracellular space, and EMS involved the integrin, complement activation, folic acid metabolism, interleukin, and lipid signaling pathways. The LASSO regression model was established using immune DEGs with an area under the curve of 0.783 for the internal dataset and 0.656 for the external dataset. Five genes with diagnostic value, *ACKR1*, *LMNB1*, *MFAP4*, *NMU*, and *SEMA3C*, were screened from M1 and M2 macrophages, activated mast cells, neutrophils, natural killer cells, follicular T helper cells, CD8^+^, and CD4^+^ cells. A protein−protein interaction network based on the immune DEGs was constructed, and ten hub genes with the highest scores were identified. Our results may provide a framework for the development of pathological molecular networks in EMS.

## Introduction

Endometriosis (EMS) is a common gynecological endocrine disease in women of childbearing age, with an incidence of approximately 10–15% in the general population and up to 50% in infertile women (1). The main pathological feature is the presence of functional endometrial tissues and cells outside the uterine cavity. The clinical manifestations include chronic pelvic pain, sexual pain, abnormal menstruation, and infertility. Because EMS has characteristics of malignant tumors, such as implantation, invasion, distant metastasis, and recurrence, it seriously affects women’s physical and mental health. “Menstrual blood reflux” is the classic pathogenesis theory of EMS. Although more than 90% of women have menstrual blood reflux, only approximately 10−15% have EMS (2). In addition, the reverse flow of endometrial debris can occur any time after menarche while EMS occurs mainly at 25−45 years of age, implying a 10-year lag between the onset of both conditions. Therefore, changes in the intraperitoneal environment, such as an imbalance of the immune system and the invasion, proliferation, and angiogenesis of ectopic endometrial tissue, may play an important role in the pathogenesis of EMS (3). Research on immune dysfunction may also contribute to disclose the role of the immune system in the pathogenesis of EMS as well as its new diagnosis and treatment strategies.

EMS can be divided into peritoneal, ovarian, and deep infiltration types and can cause other internal diseases, according to the site of lesion invasion. Each type exists either alone or in combination. The sensitivity and specificity of transvaginal ultrasonography for the diagnosis of ovarian endometriotic cysts is > 96%, and a typical homogeneous dense spot in the anechoic area has been shown; however, the endo-peritoneum was not detected. Laparoscopy is currently recognized as the best method for the diagnosis of EMS, but its operation is risky and invasive, requires certain technology and equipment, and costs are high; therefore, it is not used as a routine diagnostic method. CA125 is the most important biomarker of epithelial ovarian tumors discovered in 1980s (4). Although various imaging techniques and biomarkers have been explored as diagnostic tests for EMS, none has been used in clinical practice. Moreover, non-invasive diagnostic methods developed so far, such as ultrasound, magnetic resonance imaging, or blood tests, have not been sufficiently powerful to diagnose EMS. Therefore, a highly sensitive and specific biomarker is urgently required for the early diagnosis and treatment of this disease.

Microarray and bioinformatics analyses extend previous work based on genome-level studies to screen for genetic alterations. Gabriel et al. (5) analyzed genome-wide mRNA expression differences in the endometrium and peritoneum of patients with EMS and healthy women using EndometDB and obtained the gene characteristics of these patients. Mirza et al. (6), using the Gene Omnibus (GEO) database found that 1590 unique differentially expressed genes (DEGs; 129 upregulated and 1461 downregulated) were biologically related in 156 patients with EMS and 118 healthy controls. These findings provide a comprehensive understanding of the molecular mechanisms underlying EMS as well as new diagnosis and treatment options. Dai et al. (7) also performed a genome-wide comprehensive bioinformatics analysis between EMS lesions and healthy controls using information extracted from the GEO database. Consequently, 103 DEGs were found to be associated with cell migration, adhesion, and hypoxia-inducible factor signal transduction; these results were consistent with the immunohistochemical results, providing further understanding on the molecular mechanisms of EMS. However, these studies only noted the role of immune dysfunction in the pathogenesis of EMS. Zhong et al. (8) applied the cell-type identification by estimating relative subsets of RNA transcripts (CIBERSORT) method to conduct an in-depth analysis of the immune cell atlas and endometrial tissue using gene expression microarray data. Their results showed that the total number of macrophages in the EMS group increased, and M2 macrophages were the main subtype, consistent with the immunohistochemical results. Although this study provided a more specific understanding of the role of immune cells in the development of EMS, it was not of practical clinical value for the diagnosis of this disease.

Therefore, the present study aimed to analyze the DEGs between normal endometrium and ectopic lesions in patients with EMS and to explore the potential mechanism targets and immune microenvironment, using a variety of machine learning algorithms for feature selection to screen for diagnostic markers. The obtained diagnostic markers were verified based on the new GEO dataset. CIBERSORT was used to obtain the immune cell infiltration matrix, and the correlation and differences in infiltrated immune cells were visualized. Overall, the results allowed elucidating the molecular diagnostic mechanisms for the pathogenesis of EMS at the immune level.

## Materials and methods

### Gene expression data

GEO (http://www.ncbi.nlm.nih.gov/geo) is a public functional genomics database that contains high-throughput data for gene expression and microarray analyses. The EMS-related datasets, GSE25628, GSE6364, and GSE51981, were downloaded from the GEO database using the GEO query package (9−12). All three datasets belonged to *Homo sapiens*; the GSE6364 and GSE51981 data platform was GPL570, and the GSE25628 data platform was GPL571. GSE25628 contained 22 samples, including 6 controls and 16 diseased samples, and GSE6364 contained 37 samples, including 16 controls and 21 disease samples. All samples in these two datasets were included in the present study, and the two datasets were combined. The R package “sva” was used to remove the batch effect and construct the expression matrix (12). As an external dataset, GSE51981 was used to verify the model value of machine learning. The R package “limma” was used for standardized data processing (13).

### Identification of DEGs

According to the expression matrix resulting from the combination of datasets GSE25628 and GSE6364, the samples were classified into a normal control group and an EMS group. DEGs between the different groups were identified using the R package “limma” and absolute values of log fold-change (FC) > 1 and *P* < 0.05 as thresholds. Values of logFC > 0 and logFC < 0 identified upregulated and downregulated DEGs, respectively, in the EMS group. The results of DEG analysis are displayed as heatmaps and volcano maps produced using the R packages “pheatmap” and “ggplot2”, respectively (14, 15).

### Functional enrichment analysis

Gene Ontology (GO) categories include biological process (BP), molecular function (MF), and cellular composition (CC), which are often used for large-scale functional enrichment studies. The Kyoto Encyclopedia of Genes and Genomes (KEGG) database stores a wide range of genomic, disease, and biomedical information, while DisGeNET database is mainly used to identify disease-associated target genes. The “ClusterProfiler” R package was used for GO annotation analysis and KEGG pathway enrichment analysis of DEGs (16), and the DisGeNET database was used to analyze related diseases on which the DEGs were involved. A false discovery rate (FDR) critical value < 0.05 was considered statistically significant (17). Gene set enrichment analysis (GSEA) is a computational method used to analyze whether a particular gene set is statistically different between two biological states, and it is generally used to estimate changes in pathways and BPs in samples of expression datasets. We used GSEA to investigate differences in BPs among different populations. The “C2.cp.v7.2.Symbols.GMT [Curated]” gene set was downloaded from the Molecular Signatures database (MSigDB; http://www.gsea-msigdb.org/gsea/msigdb) for GSEA and gene sets with FDR < 0.25 were considered to be significantly enriched.

### Construction of prognostic models

Least absolute shrinkage and selection operator (LASSO) regression is a commonly used machine-learning algorithm to construct diagnostic models. Regularization is used to solve overfitting in the curve-fitting process and improve the accuracy of the model. The “Glmnet” R package was used to construct a model with differentially expressed immune genes, and the parameters Seed (1234567) and family = “binomial” were set (18).

The basic unit of Random Forest (RF) is a decision tree, and each classifier is represented by a decision tree. This computing method integrates multiple decision trees based on ensemble learning, which integrates all classification voting results and takes the category with the most votes as the final output. We used the “randomForest” R package to construct the RF prediction model with the genes screened by LASSO and the parameters Ntree = 1000 and nPerm = 50. After the model was constructed, both internal and external datasets were used to verify it. The results are presented by the receiver operating characteristic (ROC) curve drawn using the R package “pROC” (19).

### Immune infiltration analysis

The CIBERSORT software is based on the deconvolution method, which uses single-cell RNA-sequencing (RNA-seq) data to extract features and infer the composition, abundance, and proportion of immune cells in a mixture of cells. We uploaded the gene expression matrix data to CIBERSORT, combined with the LM22 eigengene matrix, filtered the output samples at *P* < 0.05, and finally obtained the immune cell infiltration matrix. The “Corrplot” R package was used to show the correlations between 22 types of immune cells (20). A histogram was drawn using the “ggplot2” R package to show the distribution of the 22 immune cell infiltrates in each sample.

### Construction of the protein−protein interaction (PPI) network

The STRING database contains 2031 species, 9.6 million proteins, and 1.38 million PPIs (21), including results obtained from experimental data, results mined from PubMed abstracts in Chinese, results from the synthesis of data from other databases, and results predicted using bioinformatic methods. Therefore, the STRING database was used to construct a PPI network of immune DEGs with a correlation coefficient of 0.4. PPI results were exported from the STRING database and visualized using Cytoscape (https://cytoscape.org). In addition, its CytoHubba plug-in was used to analyze hub genes in the PPI network, and its MCODE plug-in was used to screen subnetworks.

### Construction of the transcription factor (TF)-hub gene network

The TF-target gene network was constructed using the NetworkAnalyst database (22). NetworkAnalyst is an online visual analysis platform that can be used for gene expression analysis and meta-analysis, as it can compare and quantify gene expression, differential gene expression and enrichment analyses, PPI analysis, and multiple datasets integration analysis. It can also draw principal component analysis plots, PPI network maps, heatmaps, volcano maps, and Venn diagrams. For constructing a TF−mRNA network, we input ten hub genes into the NetworkAnalyst database, using ENCODE database information and Cytoscape for visualization.

### Construction of the microRNA (miRNA)-hub gene network

The prediction of miRNAs upstream of hub genes was based on three databases: TargetScan (23), miRDB (24), and miRWalk (25). The intersection of the prediction results of the three databases was used to construct the miRNA-hub gene network. Cytoscape was used for visualization, and the Cytohubba plug-in was used to analyze hub molecules.

### Construction of the drug molecule−hub gene network

The STITCH database contains more than 30,000 small-molecule compounds and 2.6 million PPIs from 1133 species. This database integrates information from the literature, as well as screening data from databases of multiple biological pathways, drug−target relationships, and binding affinities. The STITCH database was used to construct the small-molecule (drug)−hub gene network setting a correlation coefficient of 0.4. The results were exported from the STITCH database and visualized using Cytoscape.

### Statistical analyses

All data calculations and statistical analyses were performed using R (https://www.r-project.org/, version 4.0.2). The Student’s *t*-test was used to compare two groups of continuous variables and to analyze statistical differences in normally distributed variables. The Mann−Whitney U test (i.e., the Wilcoxon rank-sum test) was used to analyze differences between variables that were not normally distributed. The Spearman’s rank correlation test was used to calculate correlations. *P* < 0.05 was considered statistically significant.

## Results

### DEG analysis

**Figure 1:**
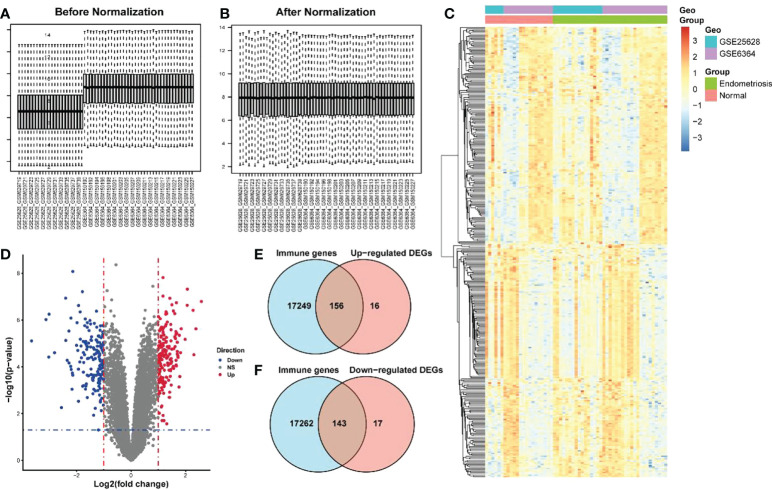
Screening of differentially expressed immune genes. **(A)** Bar chart before correction for batch joint analysis of GSE25628 and GSE6364 datasets; **(B)** Bar chart after correction for batch analysis of GSE25628 and GSE6364 datasets; **(C)** Heat map of differentially expressed genes; **(D)** Volcano map of differentially expressed genes, red represents up-regulated molecules in the disease group, blue represents down-regulated molecules in the disease group, and gray represents no differentially expressed molecules. Level of the blue line represents the p - value < 0.05 threshold, the vertical red line represents thea fold change> 1 threshold; **(E, F)** Venn diagram for screening differentially expressed immune genes.

The endometriosis-related datasets GSE25628 (platform GPL571) and GSE6364 (platform GPL570) were downloaded from the GEO database and were combined for analysis. **Figures 1A, B** are the bar charts of the dataset before and after batch correction, respectively. The results showed that the batch effect was significantly removed after correction, and the expression matrix could be used for further analysis. Setting the screening thresholds to |logFC| > 1 and *P* < 0.05, 332 DEGs (172 upregulated and 160 downregulated) were identified. The upregulated DEGs were visualized using a heatmap (**Figure 1C**) and volcano map (**Figure 1D**). After downloading the immune-related genes from the Genecards database and intersecting them with the DEGs, 156 significantly upregulated DEGs and 143 significantly downregulated DEGs were obtained. **Figures 1E, F** present the intersection results. Enrichment analysis results of GO and KEGG of differentially expressed immune genes (**Table 1**).

**Table 1:** Enrichment analysis results of GO and KEGG of differentially expressed immune genes.

ONTOLOGY	ID	Description	p.adjust	Count
BP	GO:0000070	mitotic sister chromatid segregation	4.3451E-14	25
BP	GO:0140014	mitotic nuclear division	6.616E-14	31
BP	GO:0000819	sister chromatid segregation	3.5157E-13	26
BP	GO:0000280	nuclear division	4.8997E-12	35
BP	GO:0007059	chromosome segregation	6.5876E-12	31
CC	GO:0005819	spindle	3.0387E-10	29
CC	GO:0000775	chromosome, centromeric region	1.6771E-09	21
CC	GO:0062023	collagen-containing extracellular matrix	4.7951E-09	29
CC	GO:0000776	kinetochore	7.5549E-09	17
CC	GO:0000779	condensed chromosome, centromeric region	7.5549E-09	16
MF	GO:0005539	glycosaminoglycan binding	1.0626E-05	19
MF	GO:0005201	extracellular matrix structural constituent	1.0626E-05	16
MF	GO:0008201	heparin binding	7.1072E-05	15
MF	GO:1901681	sulfur compound binding	0.00038184	17
MF	GO:0008017	microtubule binding	0.0010559	16
KEGG	hsa04978	Mineral absorption	8.2207E-05	10
KEGG	hsa04110	Cell cycle	0.00129967	12
KEGG	hsa04115	p53 signaling pathway	0.01080825	8
KEGG	hsa04610	Complement and coagulation cascades	0.02337189	8

Adjust from small to large, the top five ids of BP, CC, MF and KEGG are listed below.

### Functional enrichment analyses

**Figure 2:**
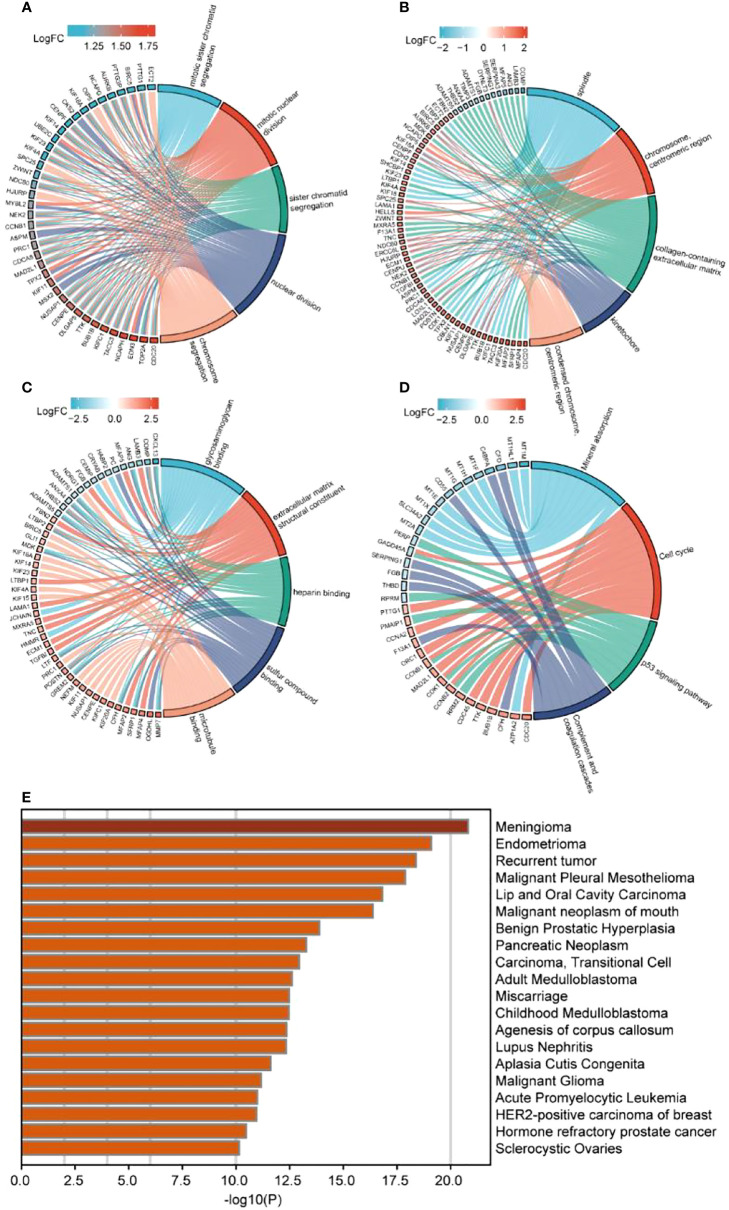
Enrichment analysis of differentially expressed immune genes. **(A–C)** GO enrichment analysis, including **(A)** BP, **(B)** CC, **(C)** MF; **(D)** KEGG enrichment analysis; **(E)** Disgenet database disease enrichment analysis.

**Figure 3:**
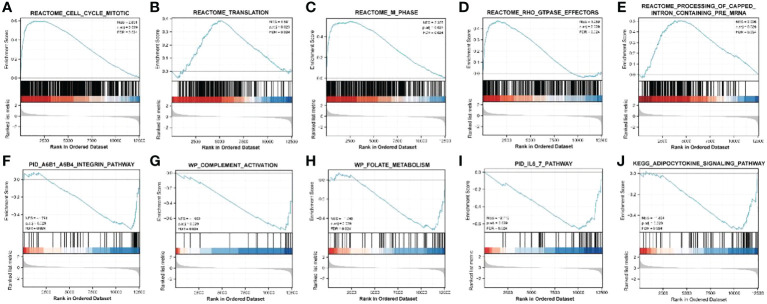
GSEA analysis of differentially expressed immune genes. **(A–E)** Five up-regulated pathways significantly enriched in the disease group; **(F–J)** Five down-regulated pathways significantly enriched in the disease group.

ClusterProfiler was used for GO and KEGG analyses of DEGs. The GO category, BP, was significantly enriched in mitotic sister chromatid segregation, mitotic nuclear division, sister chromatid segregation, nuclear division, and segregation (**Figure 2A**), while CC was significantly enriched in the spindle and chromosome, centromeric region, collagen-containing extracellular matrix, kinetochore, condensed idea, and centromeric region, among other terms (**Figure 2B**). The MF category was significantly enriched in glycosaminoglycan binding, extracellular matrix structural constituent, heparin binding, sulfur compound binding, microtubule binding, and other terms (**Figure 2C**). The significantly enriched KEGG pathways were mineral absorption, cell cycle, p53 signaling pathway, complement, and coagulation cascades (**Figure 2D**). The DisGeNET database enrichment results showed that DEGs were significantly enriched in multiple diseases, including endometrioma (**Figure 2E**). GSEA analysis of differentially expressed immune genes (**Figures 3A–E**). Five up-regulated pathways significantly enriched in the disease group (**Figures 3F–J**); Five down-regulated pathways significantly enriched in the disease group.

### LASSO and random forest model construction

**Figure 4:**
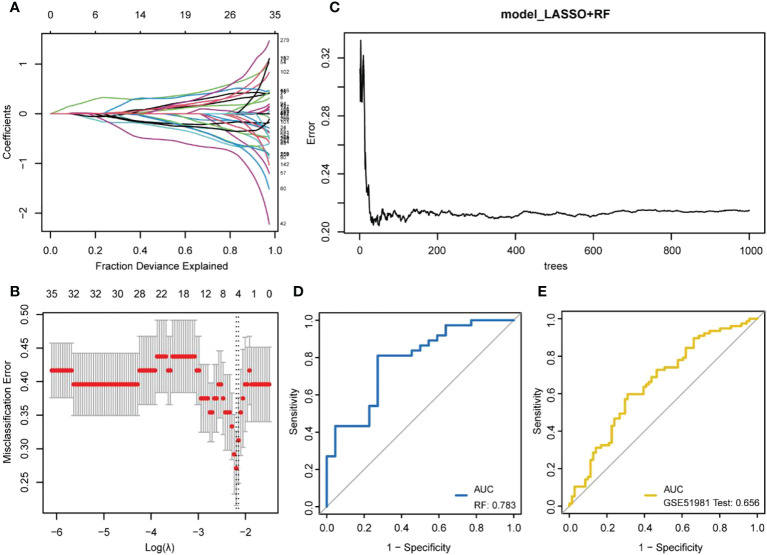
Machine learning model building. **(A, B)** LASSO regression model establishment. **(A)** the number and coefficients of selected features in different λ states during the model establishment; **(B)** the best model (left dashed line) and the simplest model (right dashed line). The upper value represents the number of features included in the model for the λ value; **(C)** The training process of random forests; **(D)** ROC curve verified by internal data sets; **(E)** ROC curve verified by external data sets.

The LASSO regression model was established using immune DEGs, and five genes with diagnostic value were screened: atypical chemokine receptor 1 (*ACKR1*), lamin B1 (*LMNB1*), microfibril-associated protein 4 (*MFAP4*), neuromedin U (*NMU*), and semaphorin 3C (*SEMA3C*). The construction process of LASSO is shown in **Figures 4A, B**. These five genes were used to build an RF prediction model as displayed in **Figure 4C**. It can be observed that the training error of the model changed with the increasing number of decision trees. The ROC curve for the internal dataset (**Figure 4D**) presented an area under the curve (AUC) of 0.783, while the ROC curve for the external dataset (**Figure 4E**) presented an AUC of 0.656.

### Immune infiltration analysis

**Figure 5:**
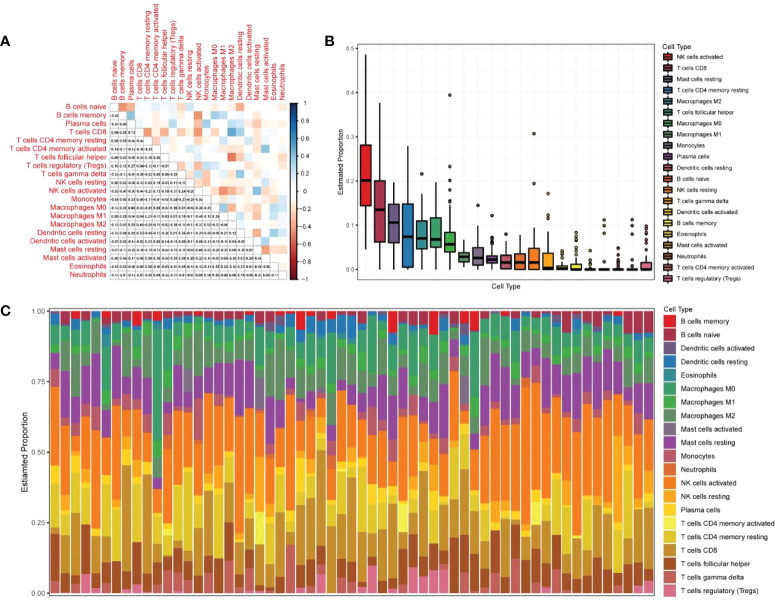
Analysis of immune infiltration results. **(A)** Correlation analysis between different kinds of immune cells, red represents positive correlation, blue represents negative correlation, the darker the color, the higher the correlation; **(B)** the proportion of different immune cells in all samples; **(C)** The proportion of different immune cells in a single sample.

**Figure 6:**
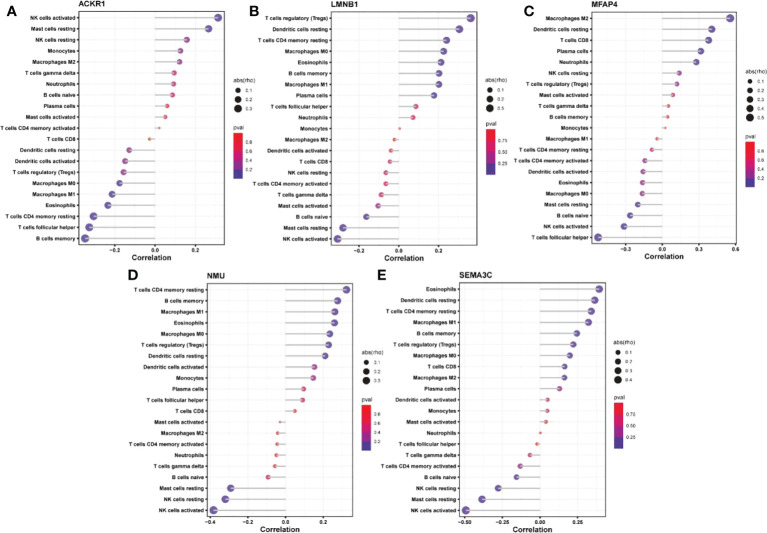
Correlation analysis between diagnostic markers and immune cells. Correlation analysis between **(A)** ACKR1, **(B)** LMNB1, **(C)** MFAP4, **(D)** NMU, **(E)** SEMA3C and different immune cells.

The expression matrices of the GSE25628 and GSE6364 combined datasets were used for immune infiltration analysis. **Figure 5A** shows the correlations between the different types of immune cells. **Figure 5B** shows the proportions of the different immune cells in all samples; natural killer (NK) cells showed the highest proportion. **Figure 5C** shows the proportions of the different immune cells in a single sample. Correlation analysis between diagnostic markers and immune cells. Correlation analysis between (A) ACKR1, (B) LMNB1, (C) MFAP4, (D) NMU, (E) SEMA3C and different immune cells (**Figures 6A–E**). Diagnostic marker genes and expression information obtained by machine learning algorithm (**Table 2**). Specific R values and P values of correlation analysis between immune cell matrix obtained by CIBERSORT and all diagnostic markers R value (**Table 3**).

**Table 2:** Diagnostic marker genes and expression information obtained by machine learning algorithm.

Gene	Direction	Log2FC	Description
ACKR1	Down	-1.37	A glycosylated membrane protein and a non-specific receptor for several chemokines
LMNB1	Up	1.63	One of the two B-type lamin proteins and a component of the nuclear lamina
MFAP4	Up	1.79	An extracellular matrix protein involved in cell adhesion or intercellular interactions
NMU	Up	1.38	A member of the neuromedin family of neuropeptides
SEMA3C	Up	1.05	A secreted glycoprotein involved in the regulation of developmental processes

**Table 3:** Specific R values and P values of correlation analysis between immune cell matrix obtained by CIBERSORT and all diagnostic markers R value.

cell	ACKR1	LMNB1	MFAP4	NMU	SEMA3C
B cells naive	0.086218	-0.1634	-0.26303	-0.0924	-0.15558
B cells memory	-0.34715	0.202323	0.043158	0.275835	0.24457
Plasma cells	0.060172	0.177033	0.315698	0.095549	0.129337
T cells CD8	-0.02677	-0.04457	0.379162	0.048974	0.16354
T cells CD4 memory resting	-0.30521	0.23993	-0.08449	0.322706	0.340325
T cells CD4 memory activated	0.019954	-0.06503	-0.14176	-0.04403	-0.13072
T cells follicular helper	-0.32644	0.085612	-0.52515	0.090168	-0.01913
T cells regulatory (Tregs)	-0.15591	0.361375	0.119932	0.228279	0.221031
T cells gamma delta	0.09479	-0.08769	0.049566	-0.05615	-0.06641
NK cells resting	0.157452	-0.065	0.138588	-0.31981	-0.27713
NK cells activated	0.312953	-0.30811	-0.31304	-0.38214	-0.49196
Monocytes	0.126026	0.00408	0.024145	0.146755	0.048558
Macrophages M0	-0.17615	0.225443	-0.16349	0.234513	0.198017
Macrophages M1	-0.21312	0.201581	-0.04185	0.261258	0.321649
Macrophages M2	0.121307	-0.02181	0.555588	-0.04192	0.162034
Dendritic cells resting	-0.12817	0.304566	0.40493	0.210099	0.363694
Dendritic cells activated	-0.14842	-0.03924	-0.157	0.152858	0.050094
Mast cells resting	0.265782	-0.28086	-0.2001	-0.29089	-0.38439
Mast cells activated	0.05075	-0.10436	0.086846	-0.02831	0.038707
Eosinophils	-0.23401	0.212376	-0.16138	0.259252	0.393179
Neutrophils	0.091699	0.071152	0.278323	-0.04882	0.002557

### PPI networks

**Figure 7:**
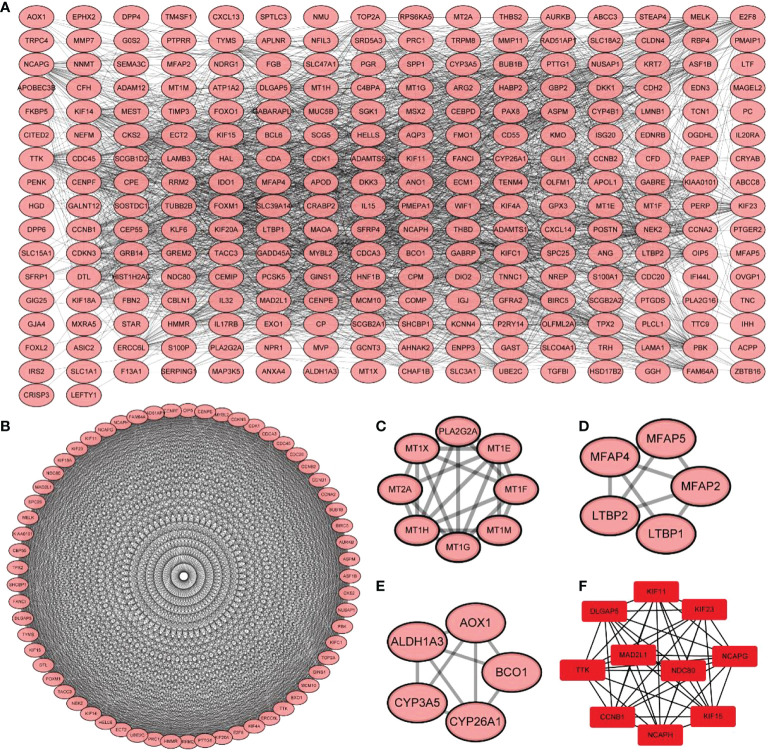
Protein-protein interaction network construction and core molecular screening of differentially expressed immune genes. **(A)** protein-protein interaction network of differentially expressed immune genes; **(B–E)** MCODE plug-in screening subnetwork; **(F)** Cytohubba plug-in screening core molecules.

**Figure 8:**
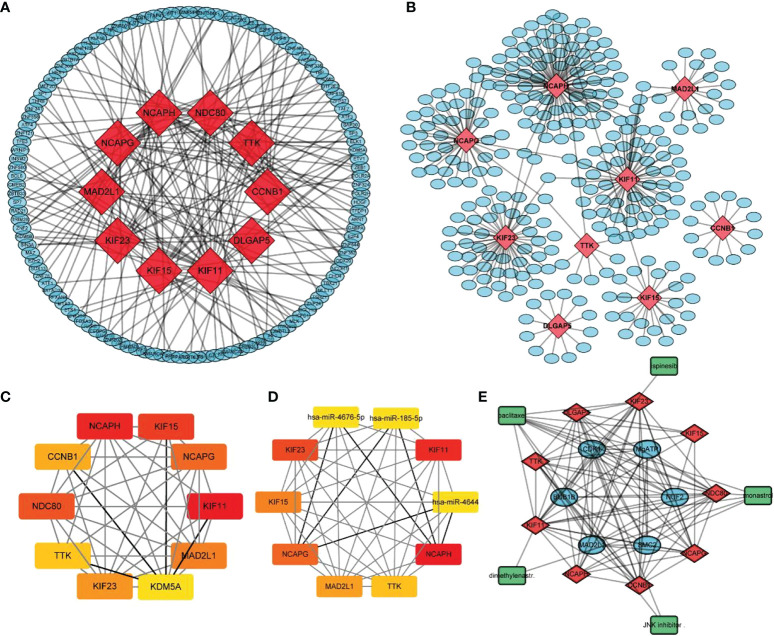
Analysis of core molecule interaction network. **(A)** TF-mrna regulatory network, red square represents hub gene, blue circle represents upstream transcription factors; **(B)** mirNA-mrna regulatory network, red square represents hub gene, blue circle represents its upstream miRNA; **(C)** TF-mrna regulatory network hub molecule; **(D)** Hub molecule of mirNA-mrna regulatory network; **(E)** Drug small molecule-mrna regulatory network, blue circle represents the protein interacting with Hub gene, red diamond represents Hub gene, green square represents the drug small molecule interacting with hub gene.

Using the STRING database, 299 PPI networks with immune DEGs were constructed using a screening threshold of 0.4. Cytoscape was used to visualize the results, as shown in **Figure 7A**. Using the MCODE plug-in of Cytoscape four subnetworks were identified (**Figures 7B−E**). Finally, using the Cytohubba plug-in of Cytoscape allowed identifying several hub genes in the PPI networks, including *KIF11, KIF23, NCAPG, KIF15, NCAPH, CCNB1, TTK, DLGAP5, MAD2L1*, and *NDC80*, as shown in **Figure 7F**. Analysis of core molecule interaction network. (A) TF-mrna regulatory network, red square represents hub gene, blue circle represents upstream transcription factors; (B) mirNA-mrna regulatory network, red square represents hub gene, blue circle represents its upstream miRNA; (C) TF-mrna regulatory network hub molecule; (D) Hub molecule of mirNA-mrna regulatory network; (E) Drug small molecule-mrna regulatory network, blue circle represents the protein interacting with Hub gene, red diamond represents Hub gene, green square represents the drug small molecule interacting with hub gene (**Figures 8A–E**).

## Verification of ACKR1, LMNB1, MFAP4, NMU, and SEMA3C expression in endometrial tissue samples

**Figure 9:**
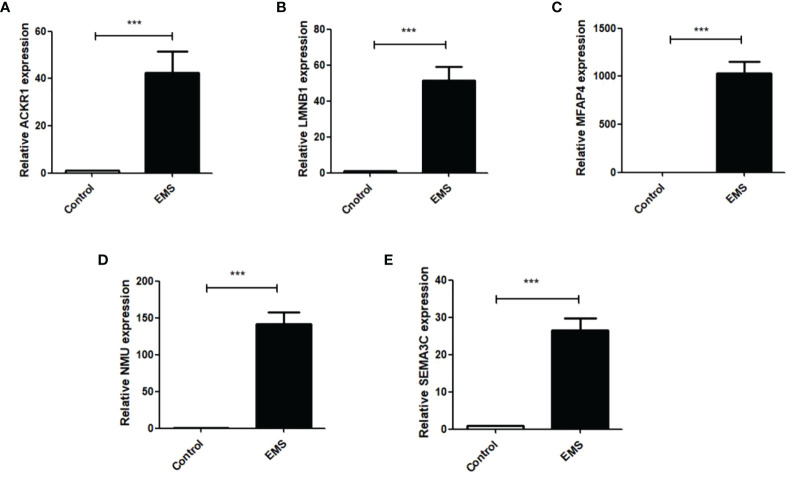
Q RT-PCR was used to detect the mRNA expressions of ACKR1, LMNB1, MFAP4, NMU and SEMA3C in normal female endometrial tissues and ectopic endometrial tissues of endometriosis patients. The results showed that: **(A)** the expression of ACKR1 was increased in the EMS group (***P<0.001); **(B)** the expression of LMNB1 was significantly increased in the EMS group (***P<0.001); **(C)** the expression of MFAP4 was increased in the EMS group (***P<0.001); **(D)** the expression of NMU was significantly increased in the EMS group (***P<0.001); **(E)** the mRNA expression of SEMA3C was significantly increased compared with the control group (***P<0.001).

Q RT-PCR was used to detect the expression of *ACKR1, LMNB1, MFAP4, NMU*, and *SEMA3C* mRNA in ectopic endometrial tissues of patients with endometriosis. As shown in **Figures 9A–E**, ACKR1, LMNB1, MFAP4, NMU, and SEMA3C were upregulated in ectopic endometrial tissues of patients with endometriosis compared with normal endometrium (*P* < 0.001), which was consistent with the results of bioinformatics analysis.

## Discussion

The pathogenesis of EMS is still unclear, but an increasing number of studies have shown that EMS is a chronic inflammatory disease. As the menstrual blood enters the abdominal cavity countercurrent, it induces an aseptic inflammatory response by chemotaxis of various immune cells, which secrete pro-inflammatory factors, and induces the activation of the immune response. At present, many researchers have focused on autoimmune disease mechanisms associated with EMS because most of its characteristics fit autoimmune disease classification standards, including polyclonal activation of T cells, B cells, and B cell dysfunction, increased apoptosis, tissue damage, and multiple organ dysfunction (8). Immune response disorders play an important role in EMS pathogenesis. Immune cells include innate immune cells, such as neutrophils, dendritic cells (DCs), NK cells, and acquired immune cells (T and B cells). Ectopic and *in situ* endometrium are separated by endometrial tissue, hormones, and both innate (macrophages, neutrophils, DCs, and NK cells) and adaptive (T and B cells) immune cells. In depth studies on the pathogenesis of EMS have allowed strengthening research on markers for early diagnosis of EMS. However, owing to insufficient sample size and defects of markers when used alone, the accuracy of diagnosis needs to be further improved. Efficient microarray and bioinformatics analyses that help us to understand the molecular mechanisms of EMS initiation and development are necessary to explore genetic variations and identify potential diagnostic biomarkers of this disease.

In the present study, 332 DEGs were found in EMS tissue and further screened using bioinformatics methods; among these, 156 upregulated and 143 downregulated DEGs were immune-related genes. Functional analysis of these DEGs revealed that they were significantly enriched during the cell cycle and played a key role in endometrial cell mitosis, chromosome separation, nuclear division, and intracellular compound binding. GO and KEGG enrichment analyses showed that the DEGs screened in the present study were mainly related to A6B1 and A6B4 integrin, complement activation, folic acid metabolism, interleukin (IL)-6/IL-7, adipokines, and other signaling pathways, participating in the regulation of cell proliferation, migration, nervous system development, and other BPs. Five diagnostic genes (*ACKR1, LMNB1, MFAP4, NMU*, and *SEMA3C*) were screened using the LASSO regression model, and the correlations between these diagnostic markers and different immune cells were further analyzed.

There are many theories about the pathogenesis of EMS but all have their own limitations. In 2003, M. Berkkanoglu and A. Arici of Yale University published a report on the relationship between EMS and immunity, suggesting that local immune abnormalities in the abdominal cavity (including abnormalities in NK cells toxicity, macrophages, cytotoxic T cells, and cytokine networks) increase the possibility of ectopic endometrium with reversed blood flow being implanted outside the uterine cavity (26), which would lead to the development of endotropia. According to the body against foreign body (such as countercurrent menstrual blood), both innate and acquired immunity systems are activated. Innate immunity can be inherited, does not target a particular antigen, lacks immune memory, and provides the same level of immune response to multiple stimuli of the same antigen. Acquired immunity, on the contrary, cannot be inherited, responds to specific antigens, and has memory. Maintaining homeostasis in the immune system is therefore a key factor in ensuring good health. Some researchers hypothesize that changes in the abdominal environment promote the proliferation of ectopic endometrial cells with abnormal immune responses, thus leading to the occurrence and development of EMS (27). Immune system imbalance is associated with the invasion, proliferation, and angiogenesis of ectopic endometrial tissue. In EMS, the study of immune dysfunction may contribute to the understanding of the role of the immune system in the pathogenesis of this disease and to the discovery of new therapeutic strategies for EMS.

Although EMS is a benign disease, its biological characteristics, such as metastasis, invasion, and implantation, are similar to those of tumors. It has been confirmed that cell adhesion molecules, neovascularization, and cell migration are involved in the migration and implantation of ectopic tissues (28). At the molecular level, a variety of cell adhesion molecules, cytokines, and other factors may be involved in assisting the endometrium to settle outside the uterine cavity. ACKR1 is a glycoprotein that is mainly expressed on the cell membrane surface. On the erythrocyte membrane, it can interact with chemokines to remove excess inflammatory chemokines and reduce their release into the circulatory system, thus regulating the inflammatory response (29). However, no study has found that ACKR1 is associated with EMS. LMNB1 is a member of the lamin family, which is a key component of lamin formation and a basic protein of the nuclear matrix. It has been reported that LMNB1 is involved in BPs, such as the proliferation and migration of various tumor cells (30). Although EMS is a benign gynecological disease, this chronic multifactorial disease usually exhibits many biological behaviors similar to those of tumors, such as cell invasion and metastasis. Therefore, abnormal cell migration and invasion are important for the pathogenesis of EMS. MFAP4, a member of the extracellular matrix, acts as a ligand and participates in the recognition of adhesion and biochemical interactions between cells, as well as in the formation of elastic fibers (31). Studies have shown that the upregulation of MFAP4 can inhibit the effect of miRNA (Mir)-147b on cell malignant invasiveness (32, 33). NMU is a neuropeptide secreted by the cholinergic, non-cholinergic, and sensory nervous systems that is distributed in the immune organs and tissues. NMU is expressed in monocytes, DCs, and B cells and modulates immune responses. It has been reported that NMU secreted by cholinergic nerve endings in mucosa binds to NMU receptor 1 (NMUR1) on the surface of type 2 innate lymphoid cells (ILC2s), thereby activating ILC2s through this pathway and inducing the release of T helper 2 (Th2)-like cytokines (e.g., IL-4, IL-5, and IL-13) by ILC2s, thus inducing Th2-like inflammatory response (34). This also suggests that NMU is an important pro-inflammatory factor in inflammatory diseases that can promote the occurrence of inflammation by binding to NMURl. SEMA3C is overexpressed in gastric, breast, and liver cancers, and its expression level is correlated with the stage and grade of gastric and breast cancers (35). The higher the SEMA3C expression, the higher is the TNM stage. Esselens et al. found that SEMA3C promotes cell migration, which is mediated by a disintegrin and metalloproteinase with thrombospondin motifs1(ADAMTS1) and promotes its release from the cytoplasm (36). Malik et al. also found that SEMA3C plays an important role in the occurrence and development of breast cancer, can promote the proliferation, invasion, and metastasis of breast cancer cells, and is positively correlated with the TNM stage of the tumor (35). In mice, SEMA3C overexpression in lung cancer cell lines significantly enhanced the ability of tumor cells to metastasize (37). SEMA3C also enhanced endothelial cell adhesion and stimulated angiogenesis in gastric cancer tissues by activating integrin phosphorylation and promoting vascular endothelial growth factor 120 secretion (38). In the present study, bioinformatics analyses were used to screen DEGs on the EMS mRNA chip, which provided new routes for the study of the pathogenesis of this disease. It would be beneficial to increase the credibility of the obtained results using clinical samples.

Due to the limitation of screening conditions, only three datasets were included in the present study, corresponding to 22 normal endometrial tissue samples and 37 EMS tissue samples. Therefore, sample size may have been insufficient. As CIBERSORT analysis was based on limited genetic data, which may deviate from cellular atypia interactions, disease-induced disorders, or phenotypic plasticity, only 37 EMS samples were included, indicating low test efficiency. Therefore, validation using a larger sample size is required. Our dataset was built based on previous studies. The information on the processing of the original data and the development of experimental sequencing in previous studies is not known, which will directly affect the accuracy of the present study. Because bioinformatics methods and GEO database mining were used to explore potential biomarkers of EMS, the hypotheses drawn here are also based on the results of previous studies, and therefore need to be further verified by supplementary experiments. In order to verify the results of prematurity analysis, we collected 20 tissue samples from endometriosis patients treated in The 1st Hospital of Lanzhou University and 20 normal endometrial tissue samples from uterine fibroids. The expression levels of *ACKR1, LMNB1, MFAP4, NMU*, and *SEMA3C* in ectopic endometrial tissues and normal endometrial tissues were analyzed by qRT-PCR. The results showed that *ACKR1, LMNB1, MFAP4, NMU*, and *SEMA3C* were up-regulated in ectopic endometrial tissues, which was consistent with the differential expression obtained by TCGA database in our previous study.

In conclusion, 332 DEGs and ten hub genes were identified through bioinformatics analysis of EMS expression profile data. Five diagnostic biomarkers (*ACKR1*, *LMNB1*, *MFAP4*, *NMU*, and *SEMA3C*) were identified by ROC analysis of hub genes. In addition, immune cell infiltration analysis revealed that NK cells and M1 macrophages may be involved in the occurrence and development of EMS, which provides a theoretical basis for the development of new diagnostic biomarkers of this disease and further study of its molecular mechanisms. In future studies, we will verify the accuracy of the results of the present study at the molecular, cellular, and tissue levels and further explore the regulatory relationship between the five diagnostic markers, NK cells, and M1 macrophages.

## Data availability statement

Publicly available datasets were analyzed in this study. This data can be found here: The endometriosis related datasets GSE25628, GSE6364, and GSE51981 were downloaded from the GEO database through the GEOquery package.

## Author contributions

YH and QL conceived the study idea. YH, QL, and RH collected the data to be analyzed. YH, QL, RH, and RL performed the data analysis and produced the results. YH, QL, and YY wrote and revised the manuscript. All authors contributed to the article and approved the submitted version.

## Funding

Lanzhou Science and Technology Bureau Foundation (2021-RC-133), the Regional Scientists Fund of the National Natural Science Foundation of China(No. 81960275), Gansu Provincial Department of Science and Technology Foundation (21YF5FA119).

## Conflict of interest

The authors declare that the research was conducted in the absence of any commercial or financial relationships that could be construed as a potential conflict of interest.

## Publisher’s note

All claims expressed in this article are solely those of the authors and do not necessarily represent those of their affiliated organizations, or those of the publisher, the editors and the reviewers. Any product that may be evaluated in this article, or claim that may be made by its manufacturer, is not guaranteed or endorsed by the publisher.
