# Supplementary figures and images for: Five immune-related genes as diagnostic markers for endometriosis and their correlation with immune infiltration
Source: Front Endocrinol (Lausanne). 2022 Oct 6;13:1011742. doi: 10.3389/fendo.2022.1011742 (PMC9582281; doi:10.3389/fendo.2022.1011742)

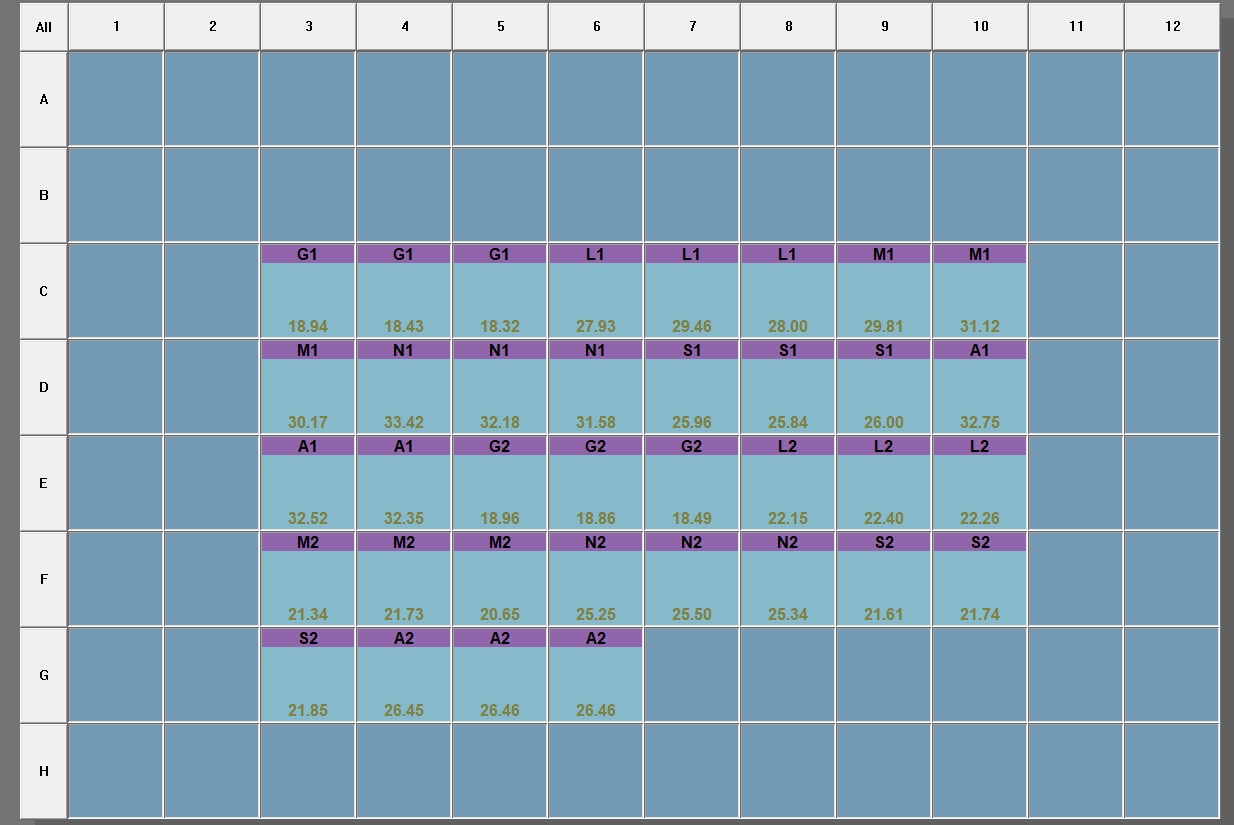

Supplement: Supplementary file 1 [file DataSheet_1.zip › excel+p/1-1.jpg]

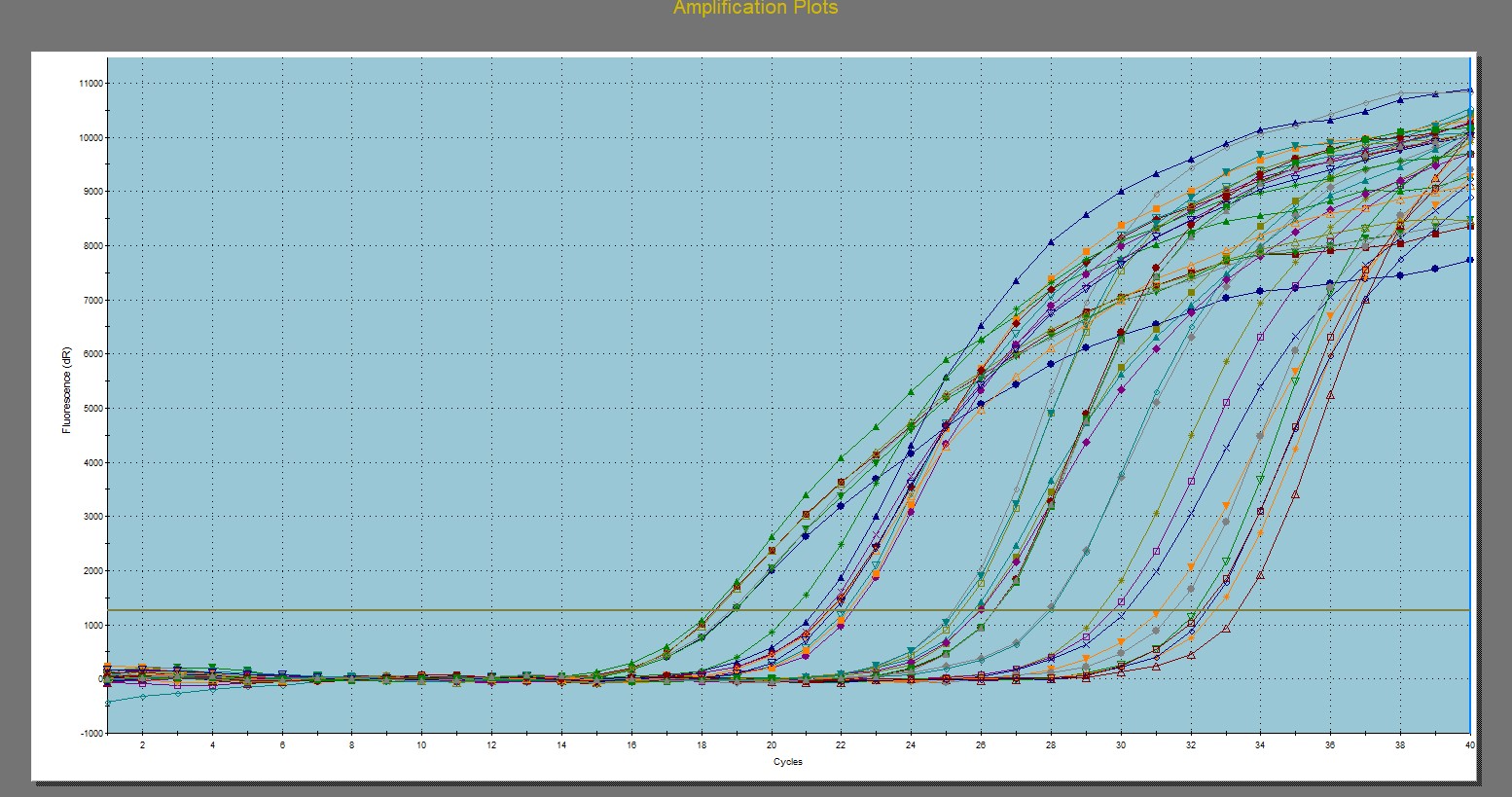

Supplement: Supplementary file 1 [file DataSheet_1.zip › excel+p/1-2.jpg]

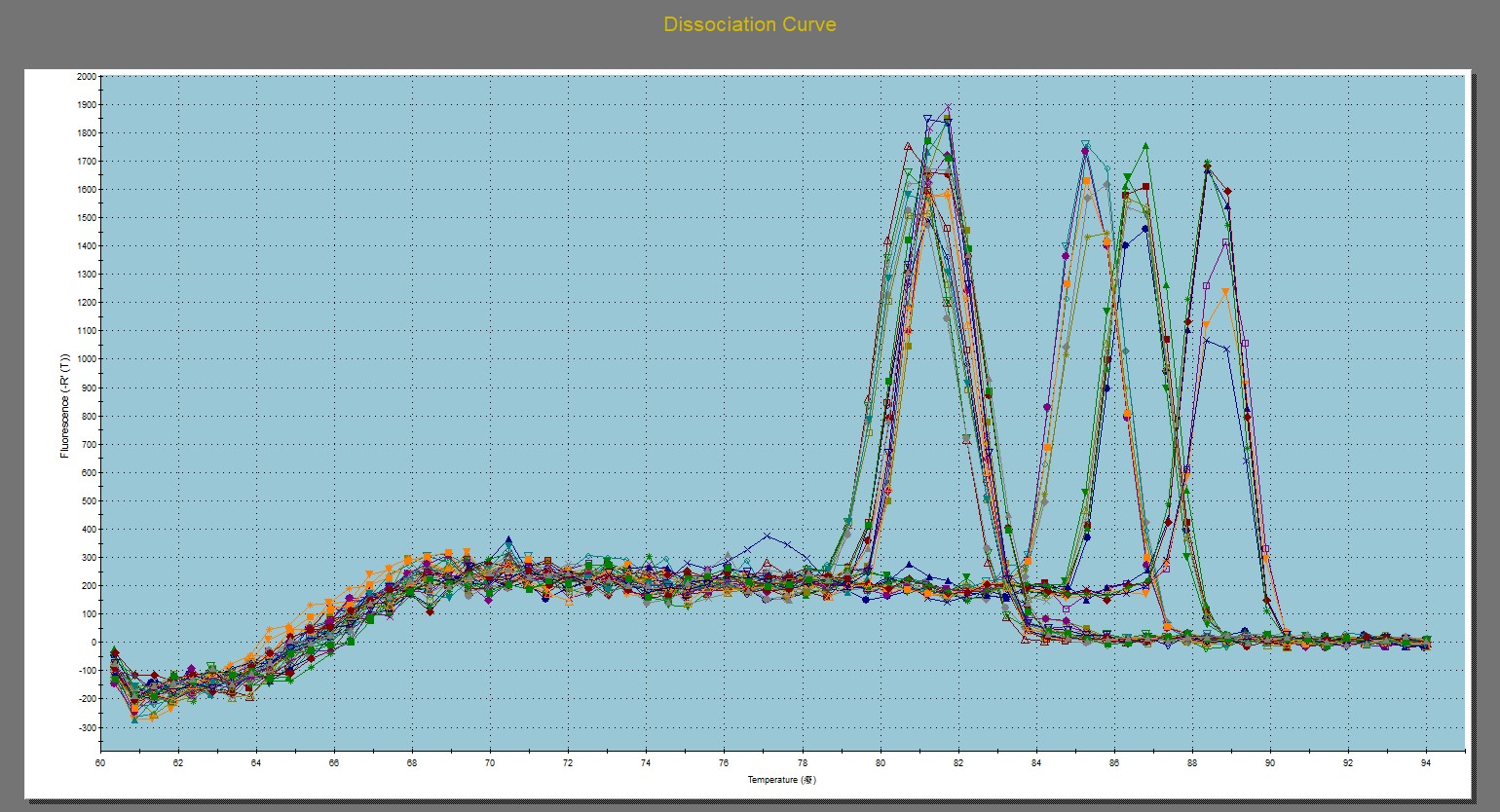

Supplement: Supplementary file 1 [file DataSheet_1.zip › excel+p/1-3.jpg]

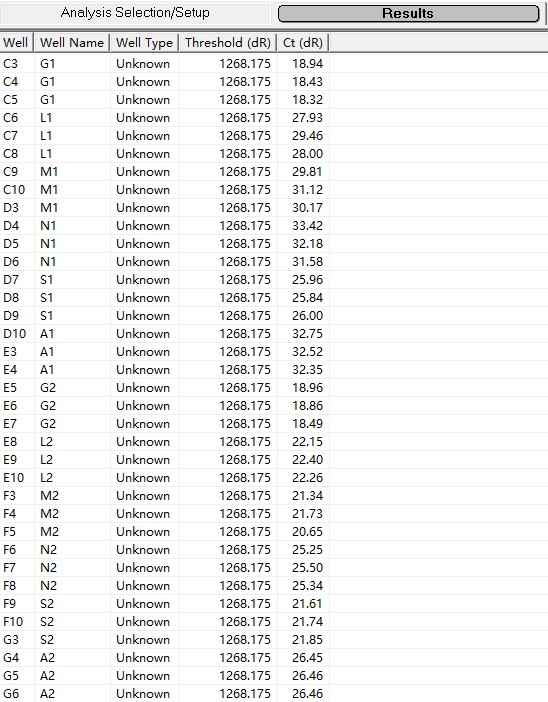

Supplement: Supplementary file 1 [file DataSheet_1.zip › excel+p/1-4.jpg]

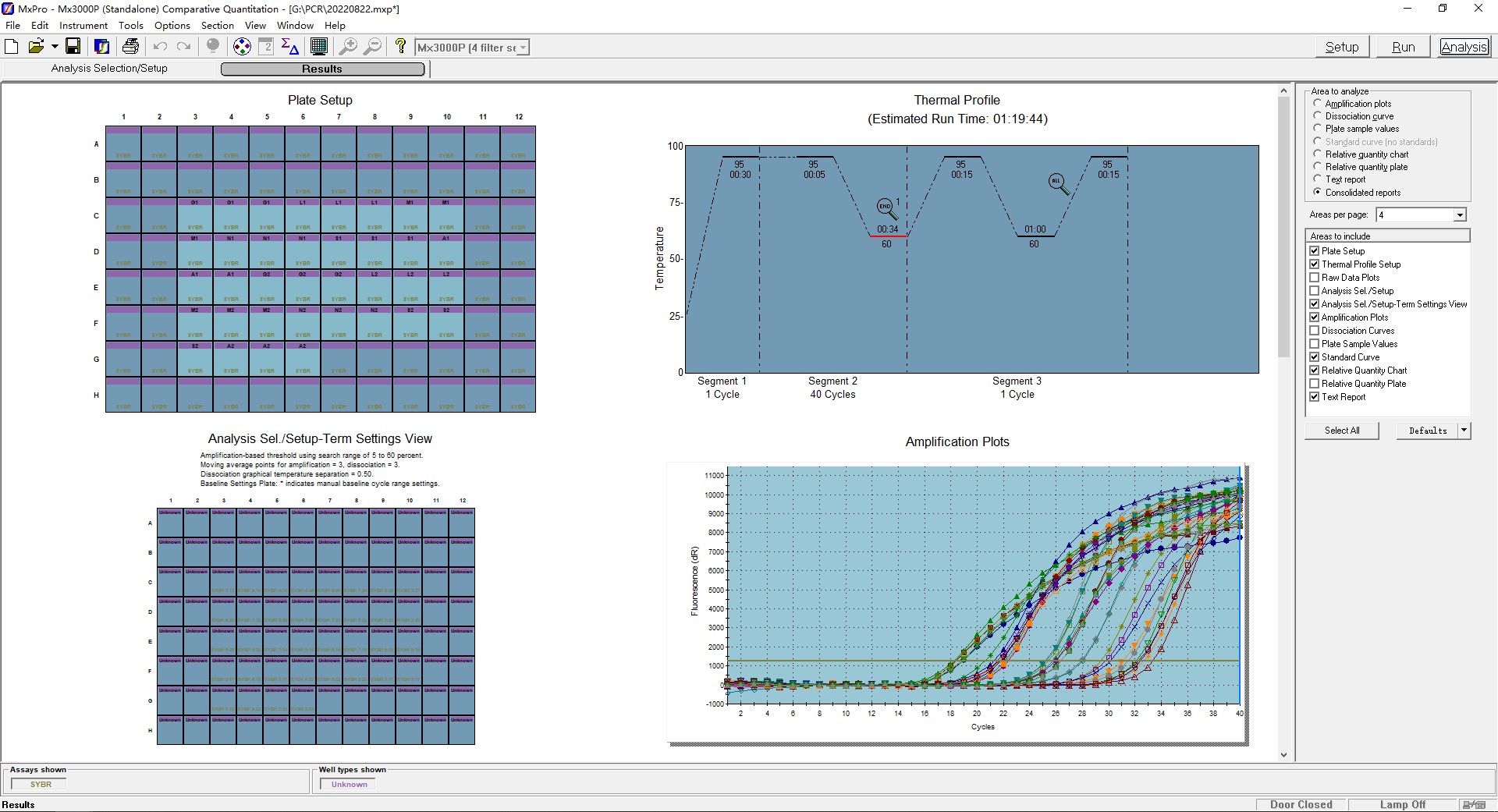

Supplement: Supplementary file 1 [file DataSheet_1.zip › excel+p/1.jpg]

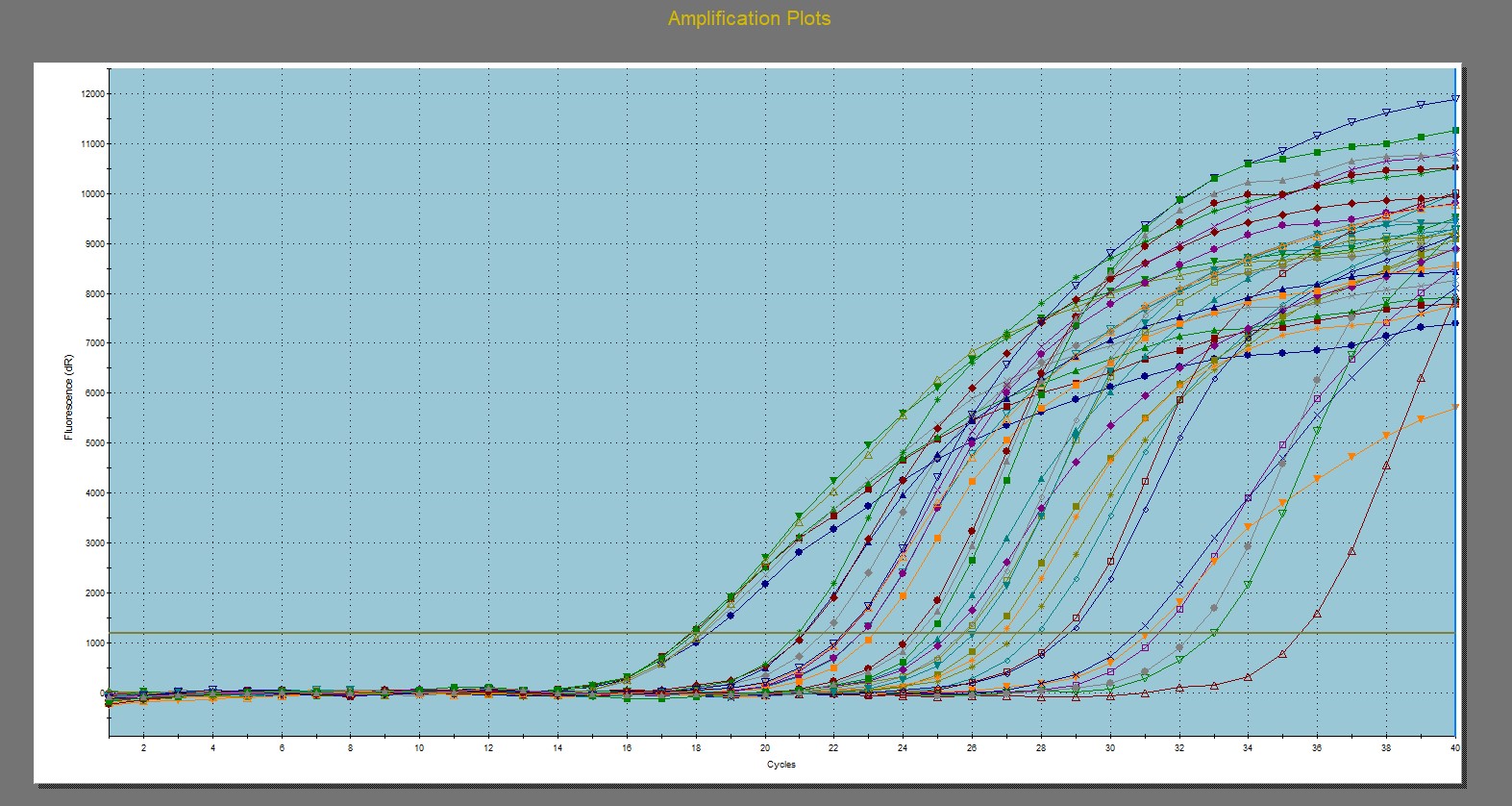

Supplement: Supplementary file 1 [file DataSheet_1.zip › excel+p/10-1.jpg]

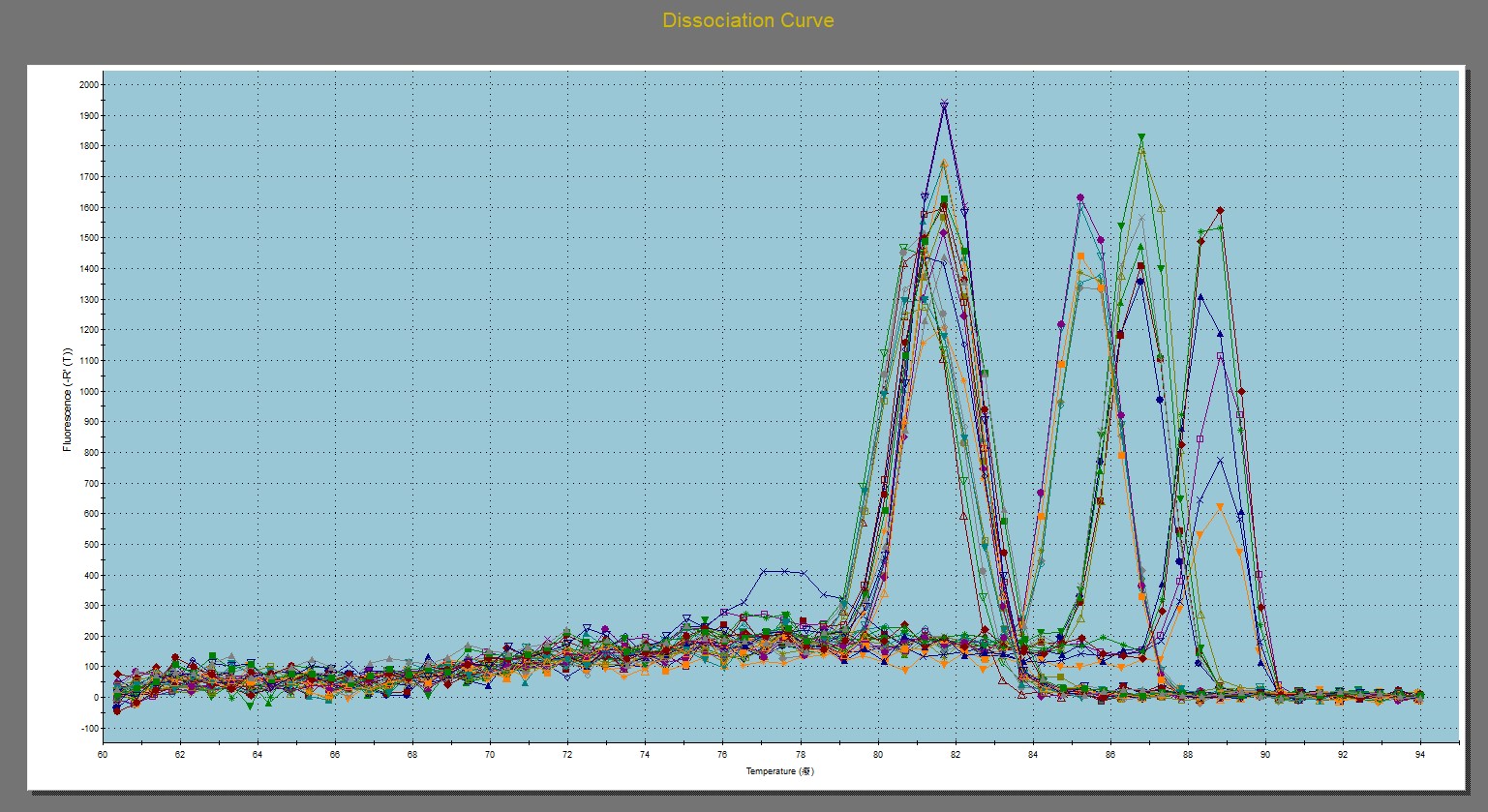

Supplement: Supplementary file 1 [file DataSheet_1.zip › excel+p/10-2.jpg]

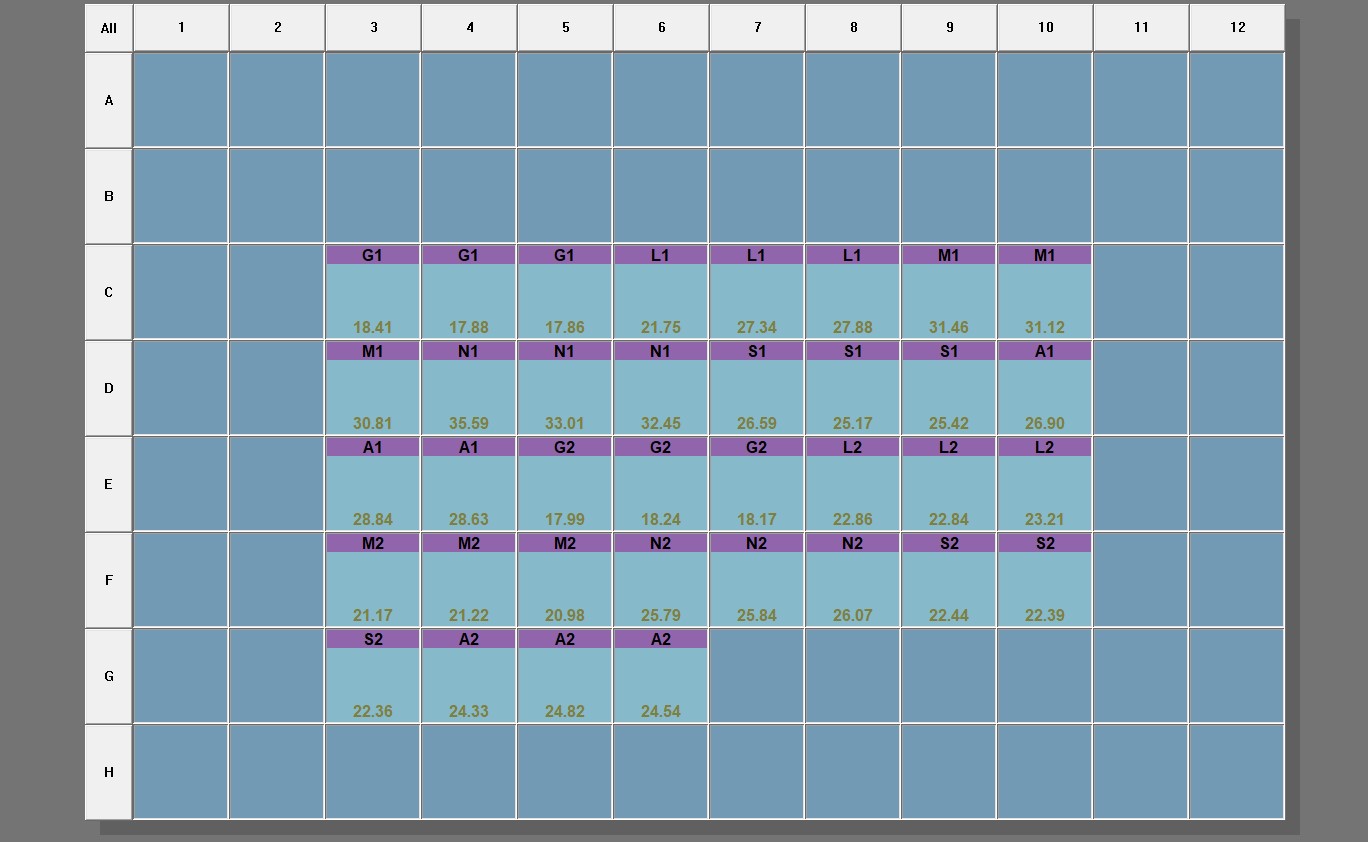

Supplement: Supplementary file 1 [file DataSheet_1.zip › excel+p/10-3.jpg]

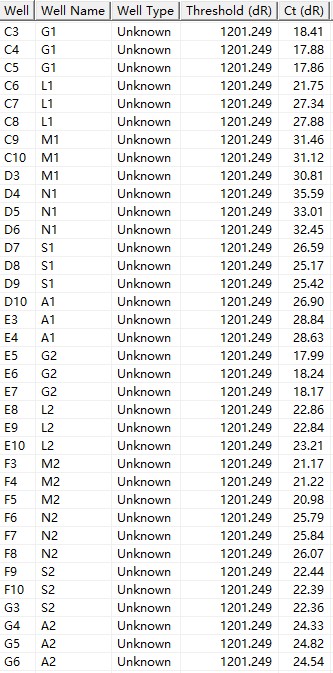

Supplement: Supplementary file 1 [file DataSheet_1.zip › excel+p/10-4.jpg]

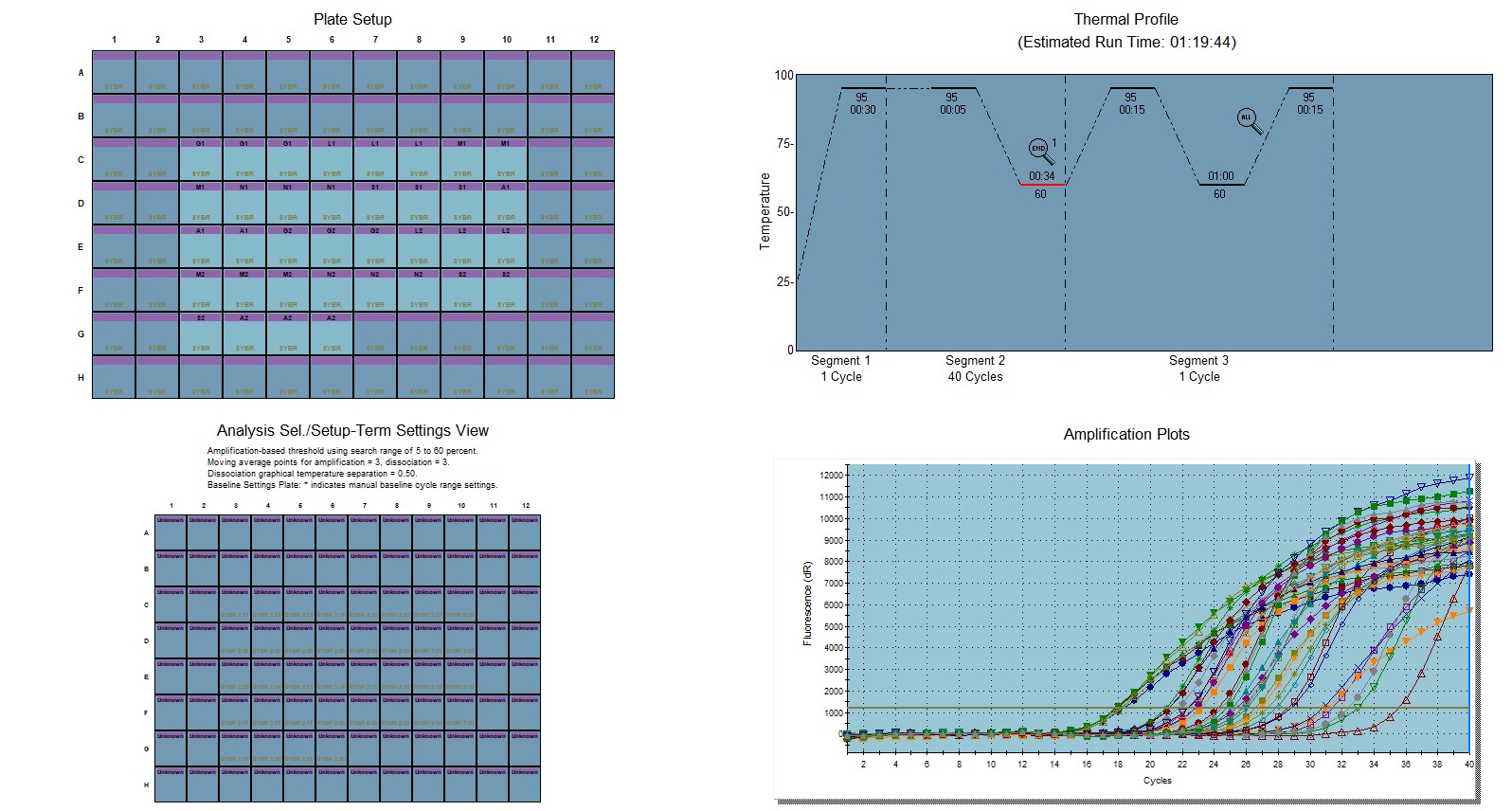

Supplement: Supplementary file 1 [file DataSheet_1.zip › excel+p/10.jpg]

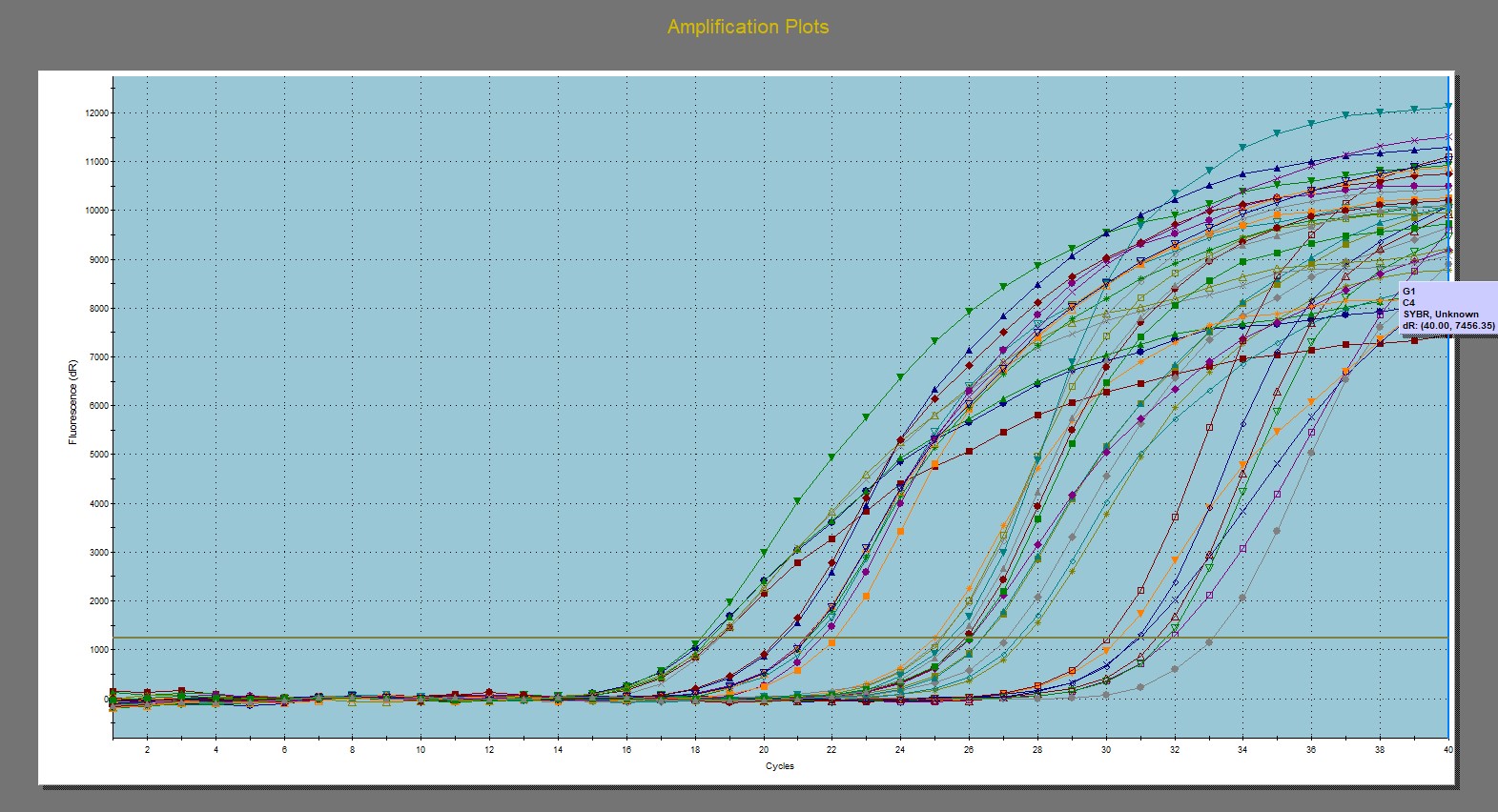

Supplement: Supplementary file 1 [file DataSheet_1.zip › excel+p/11-1.jpg]

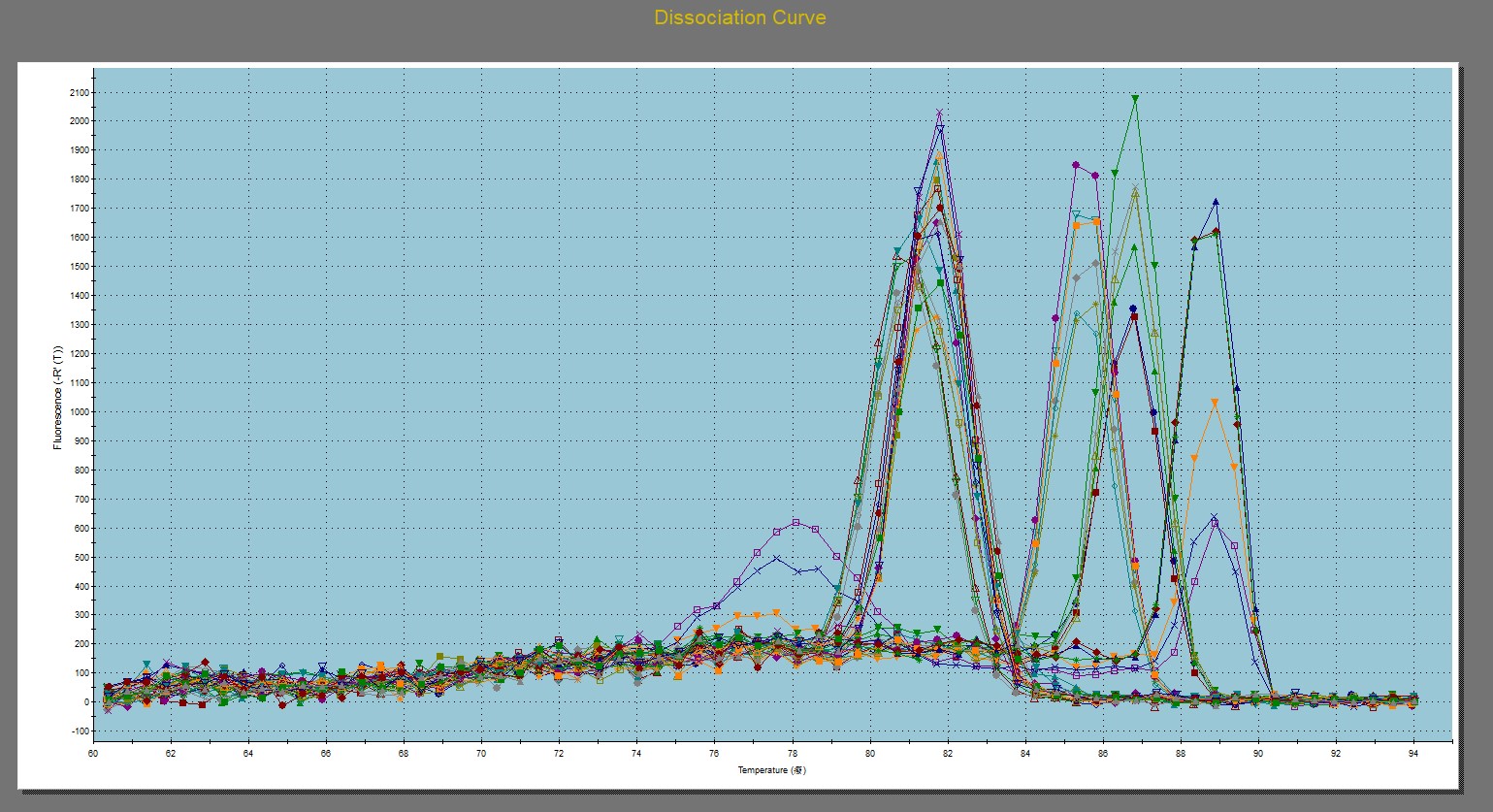

Supplement: Supplementary file 1 [file DataSheet_1.zip › excel+p/11-2.jpg]

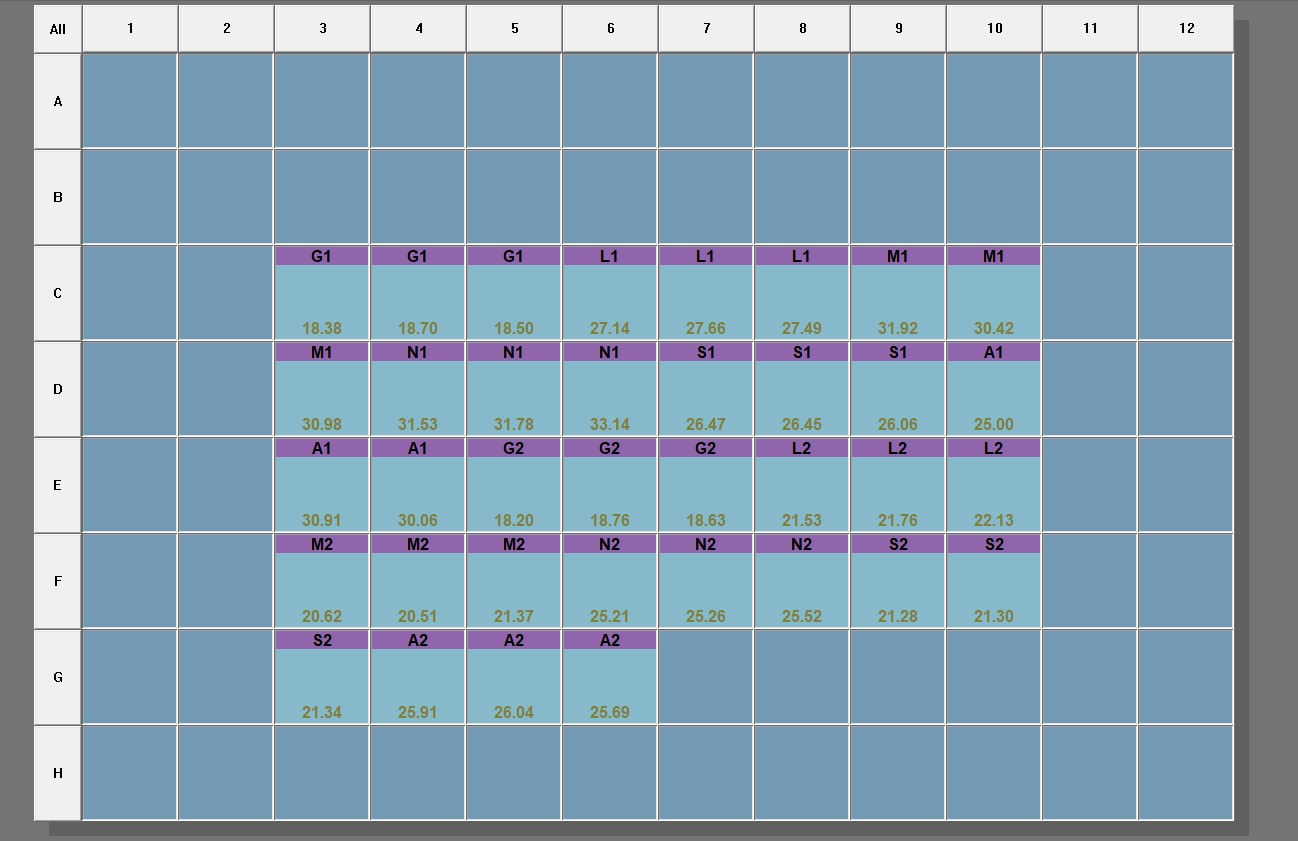

Supplement: Supplementary file 1 [file DataSheet_1.zip › excel+p/11-3.jpg]

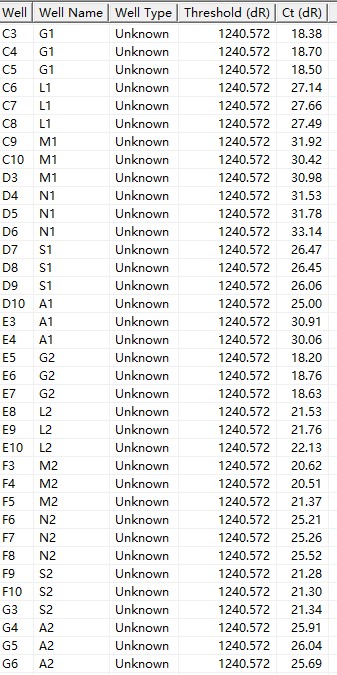

Supplement: Supplementary file 1 [file DataSheet_1.zip › excel+p/11-4.jpg]

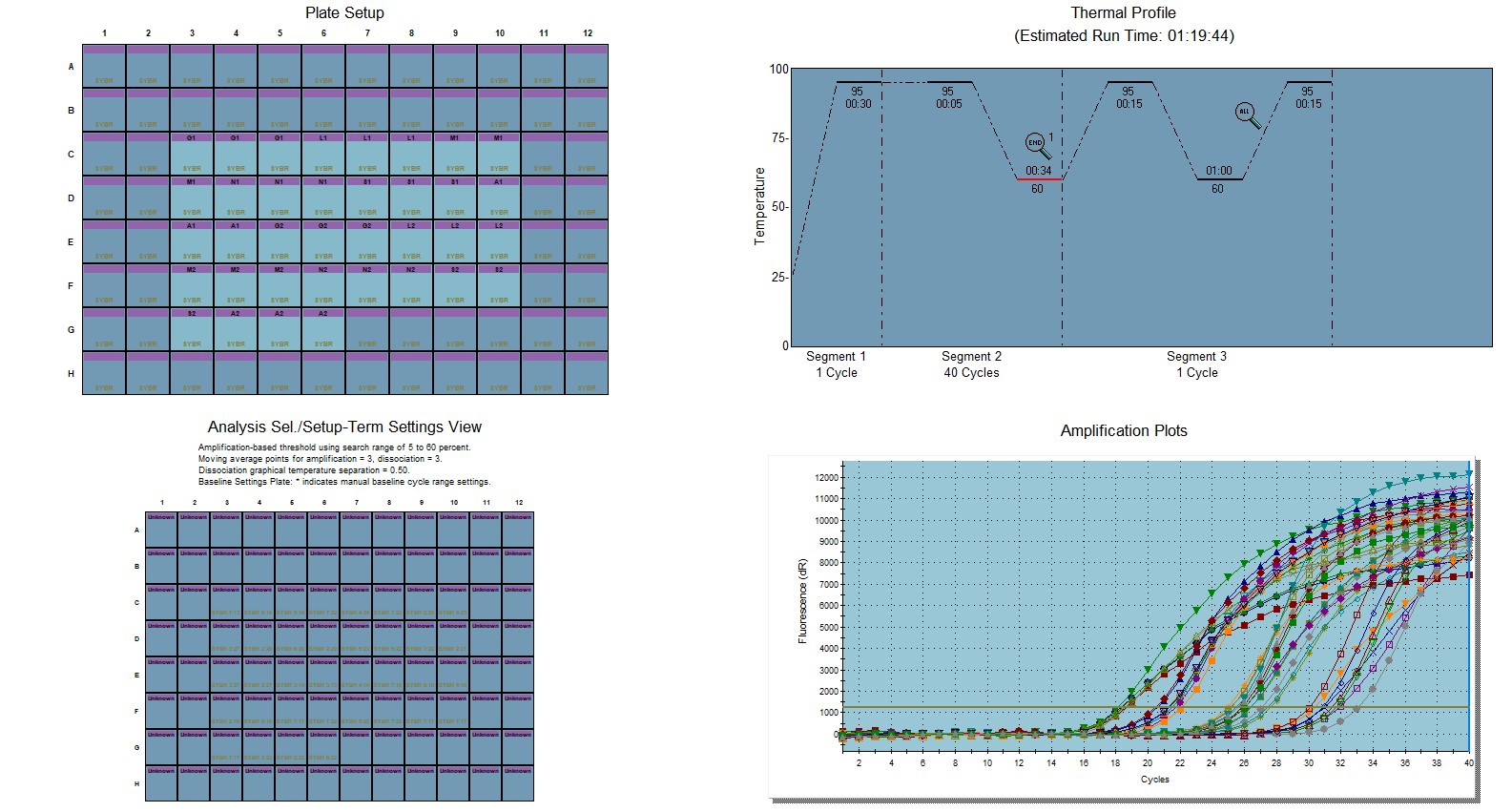

Supplement: Supplementary file 1 [file DataSheet_1.zip › excel+p/11.jpg]

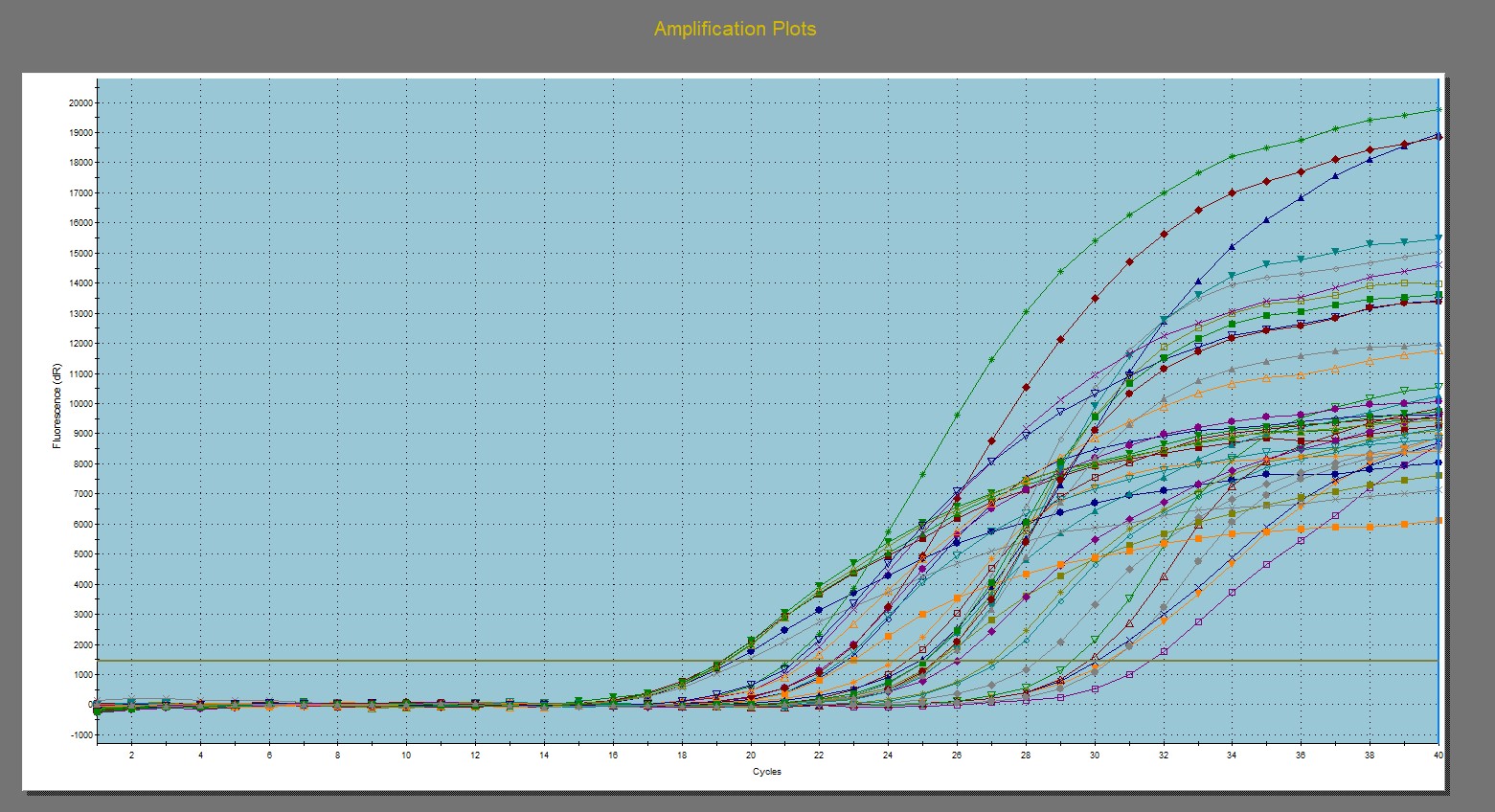

Supplement: Supplementary file 1 [file DataSheet_1.zip › excel+p/12-1.jpg]

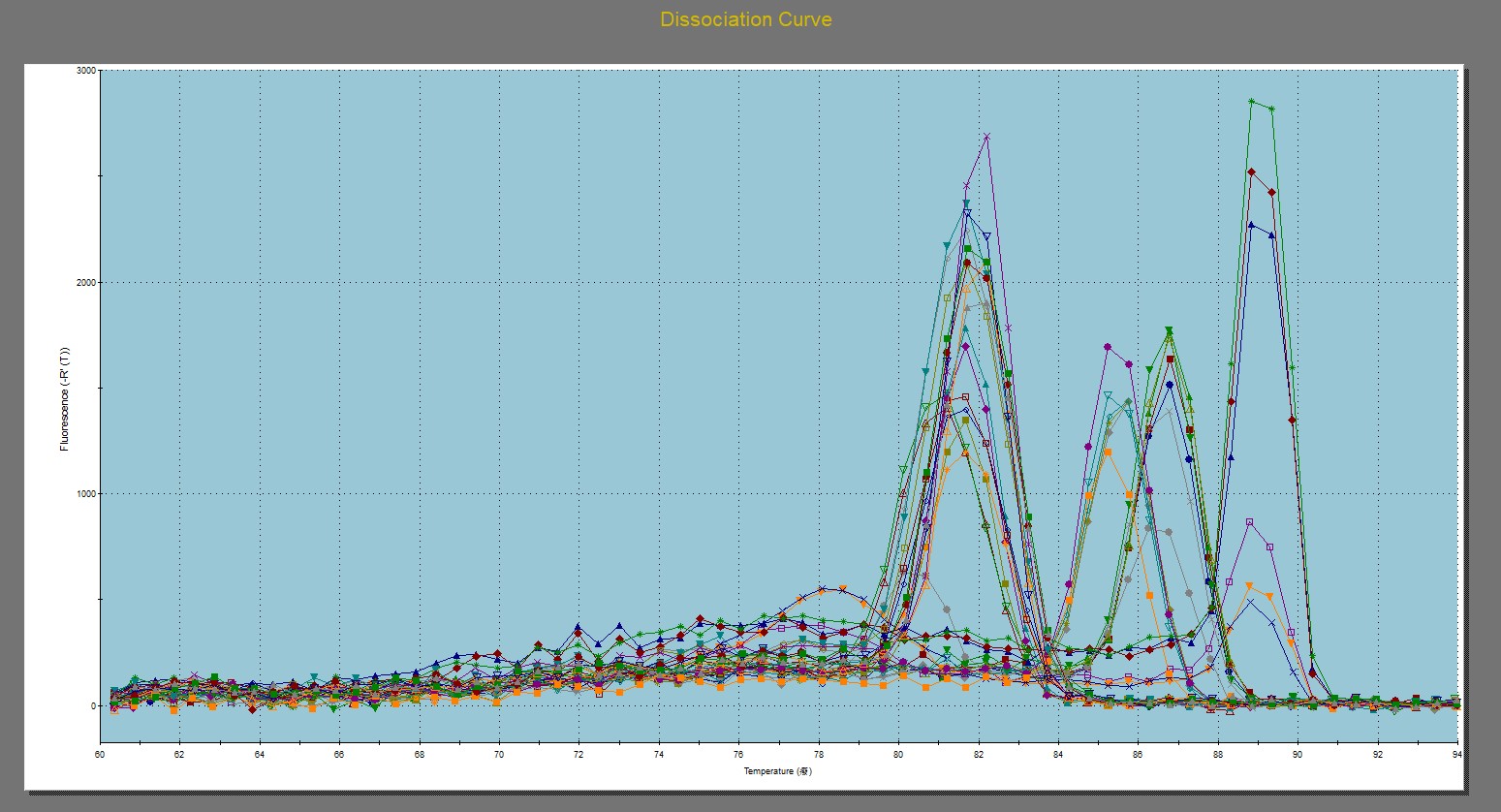

Supplement: Supplementary file 1 [file DataSheet_1.zip › excel+p/12-2.jpg]

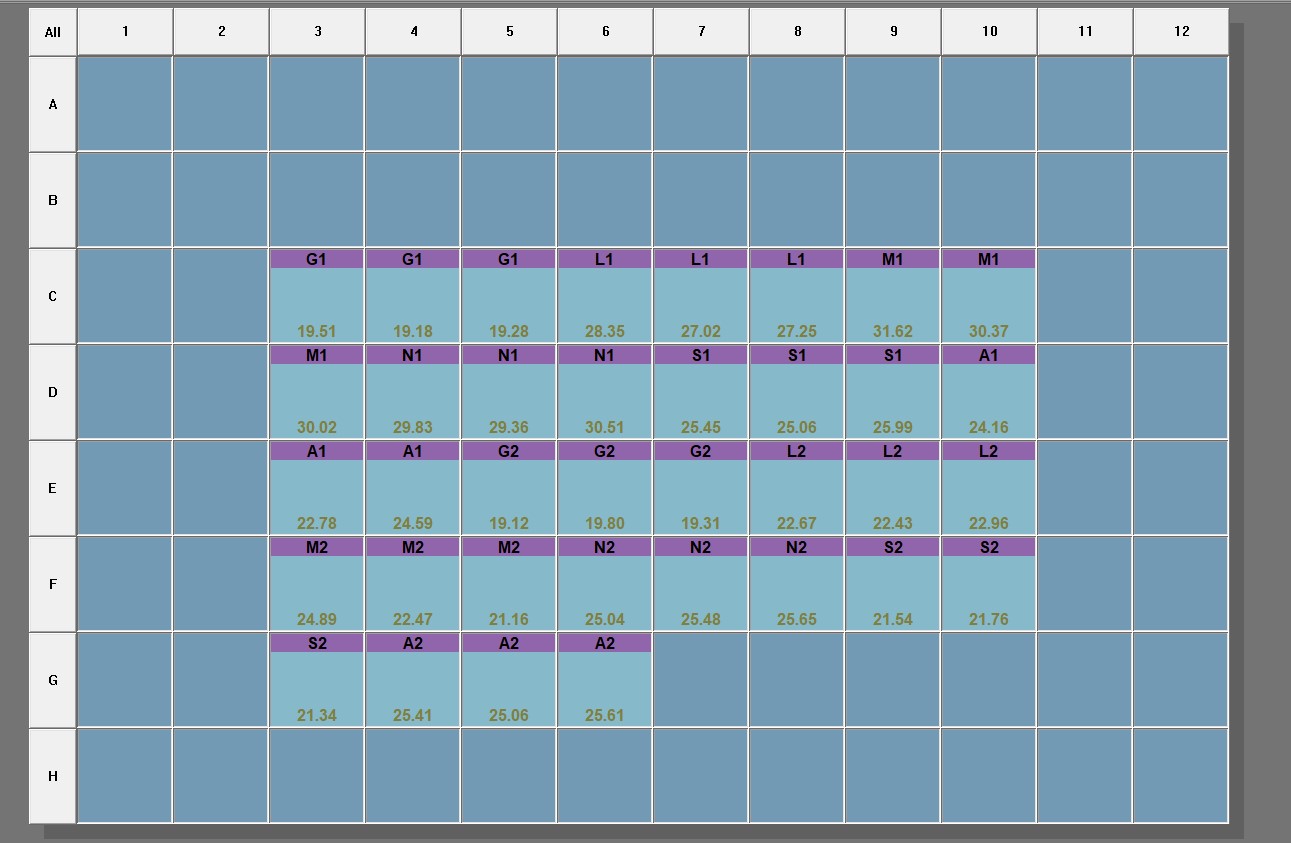

Supplement: Supplementary file 1 [file DataSheet_1.zip › excel+p/12-3.jpg]

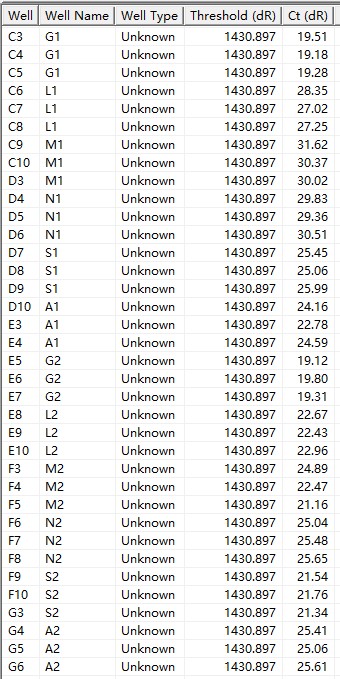

Supplement: Supplementary file 1 [file DataSheet_1.zip › excel+p/12-4.jpg]

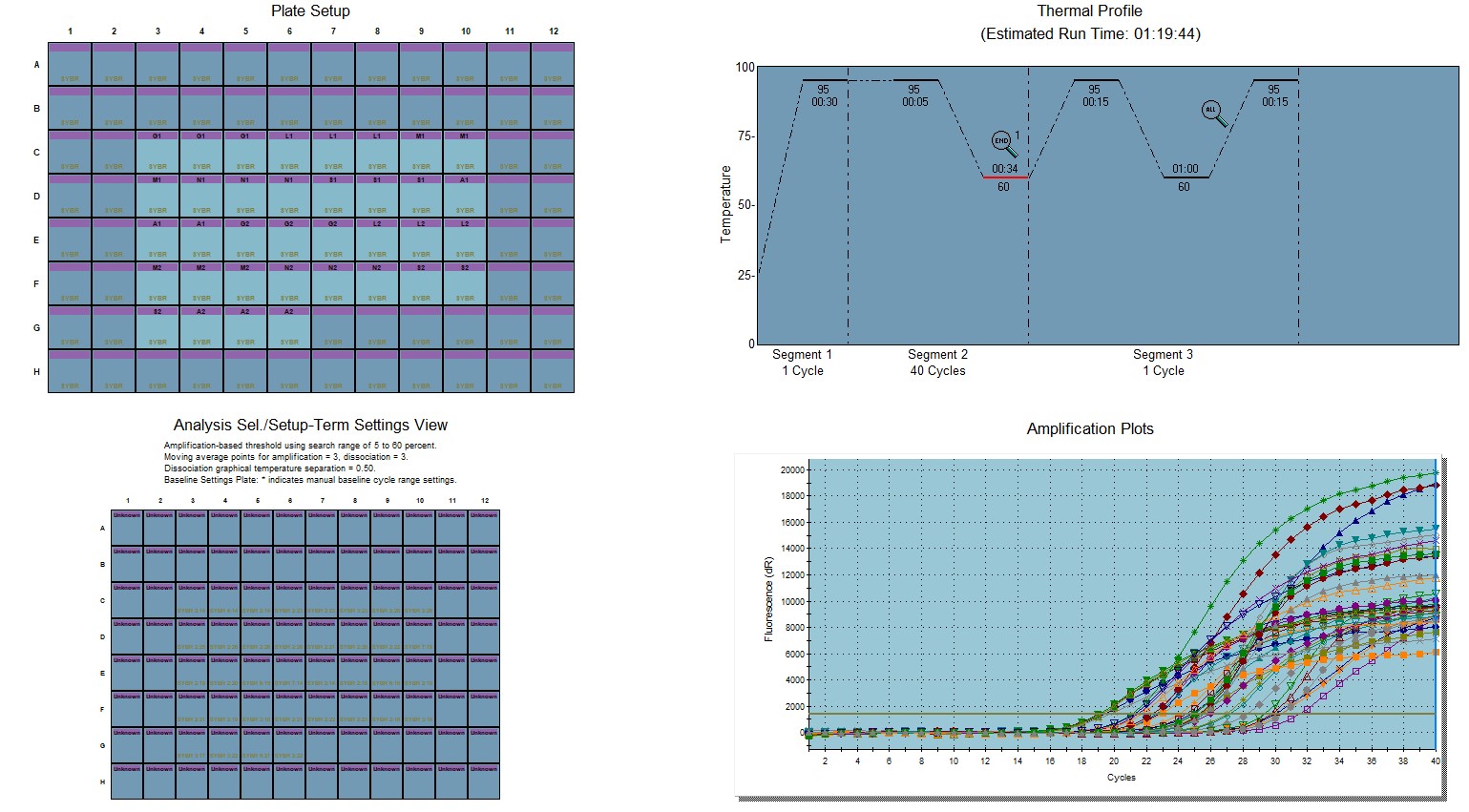

Supplement: Supplementary file 1 [file DataSheet_1.zip › excel+p/12.jpg]

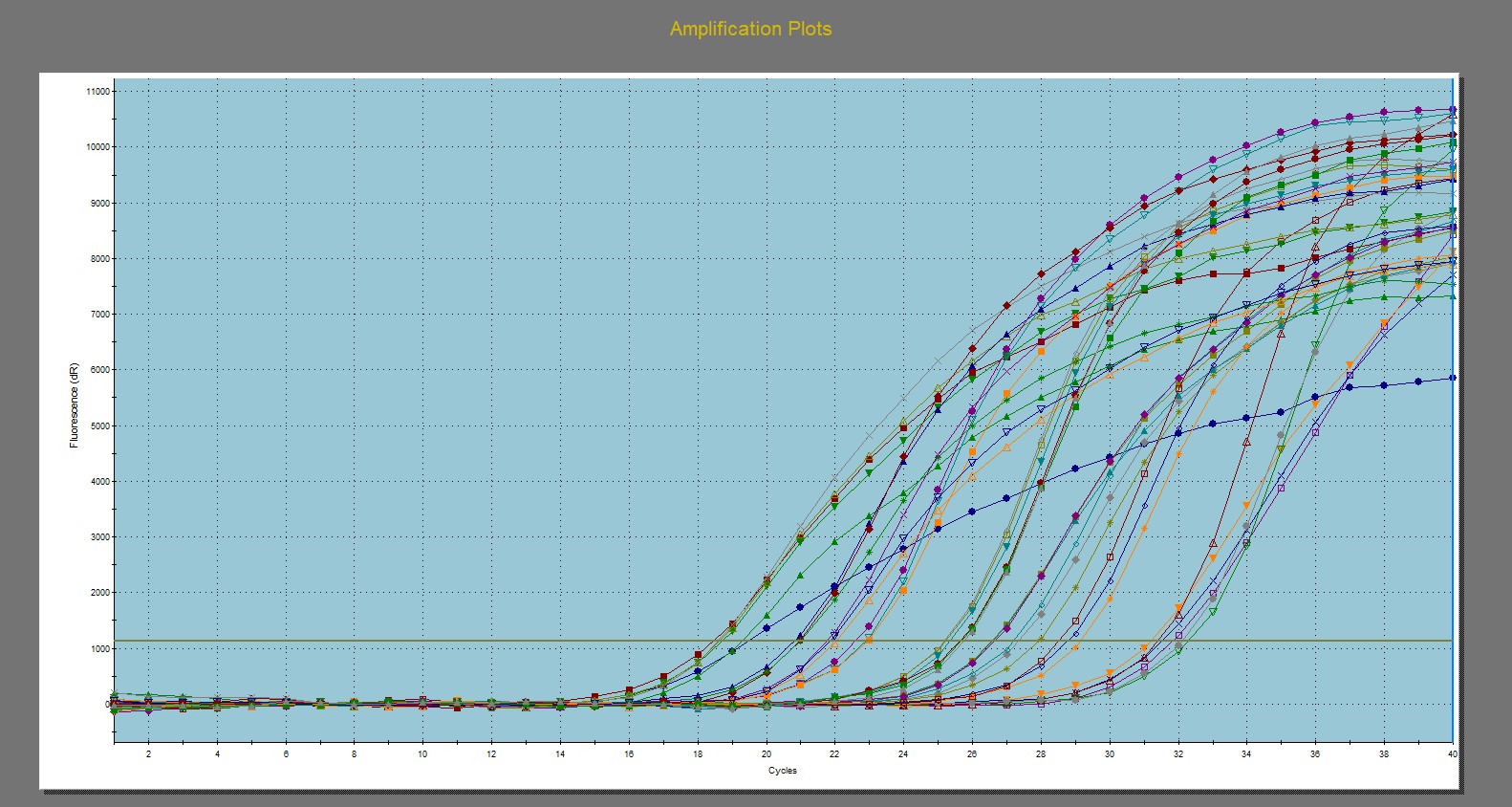

Supplement: Supplementary file 1 [file DataSheet_1.zip › excel+p/13-1.jpg]

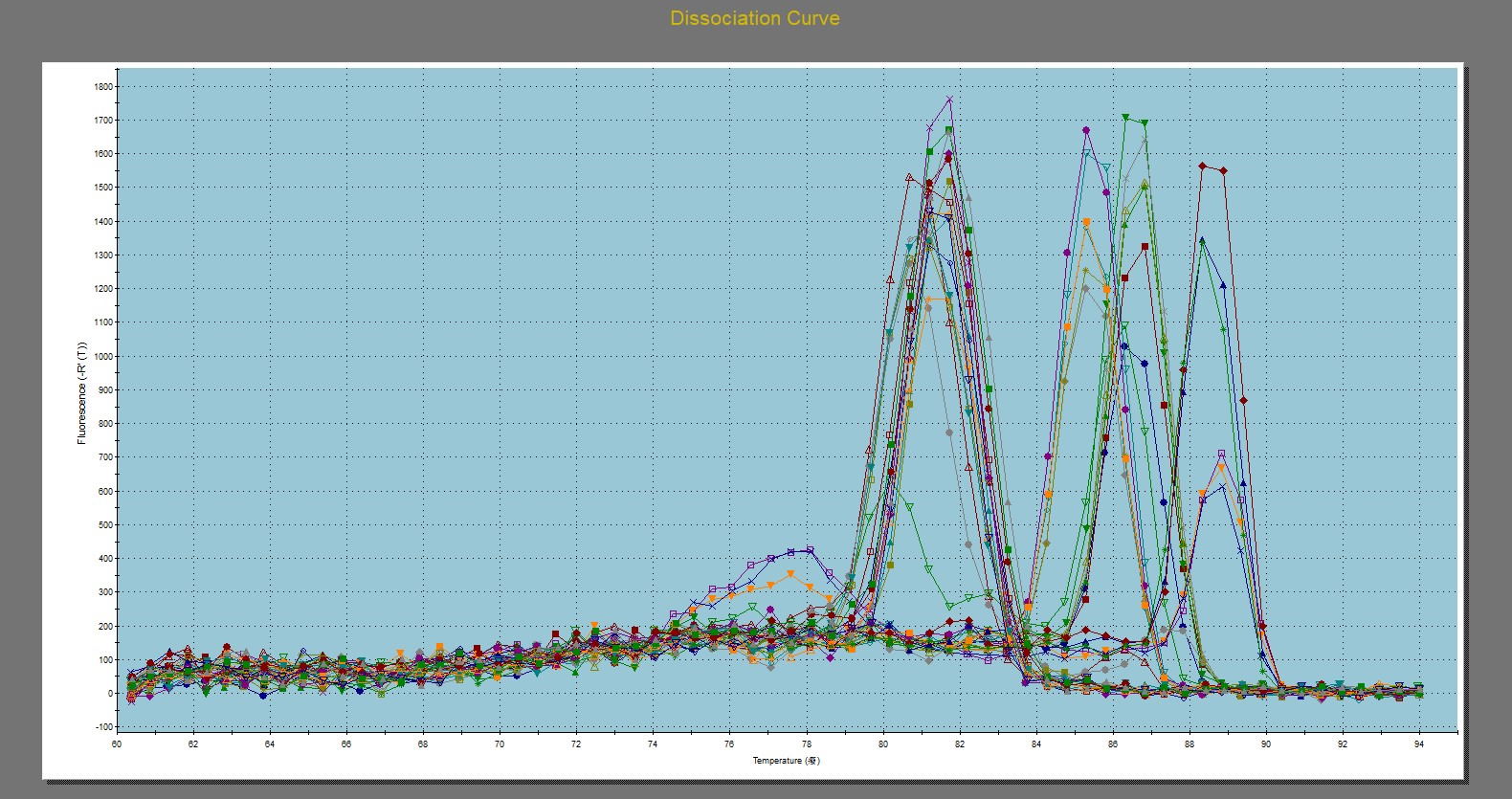

Supplement: Supplementary file 1 [file DataSheet_1.zip › excel+p/13-2.jpg]

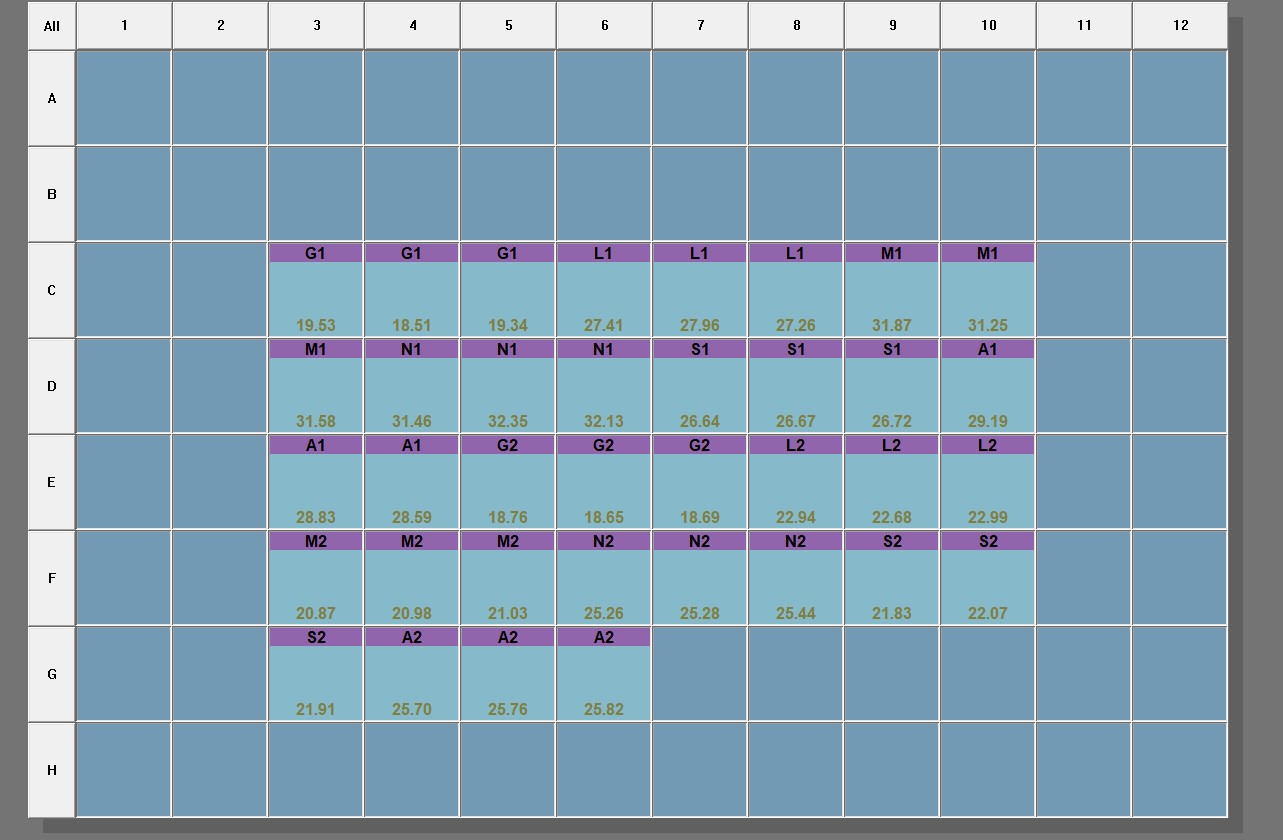

Supplement: Supplementary file 1 [file DataSheet_1.zip › excel+p/13-3.jpg]

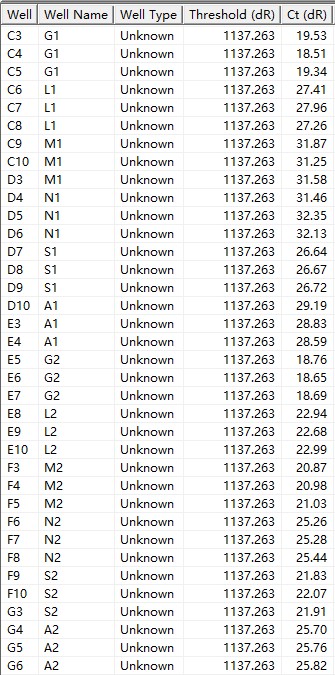

Supplement: Supplementary file 1 [file DataSheet_1.zip › excel+p/13-4.jpg]

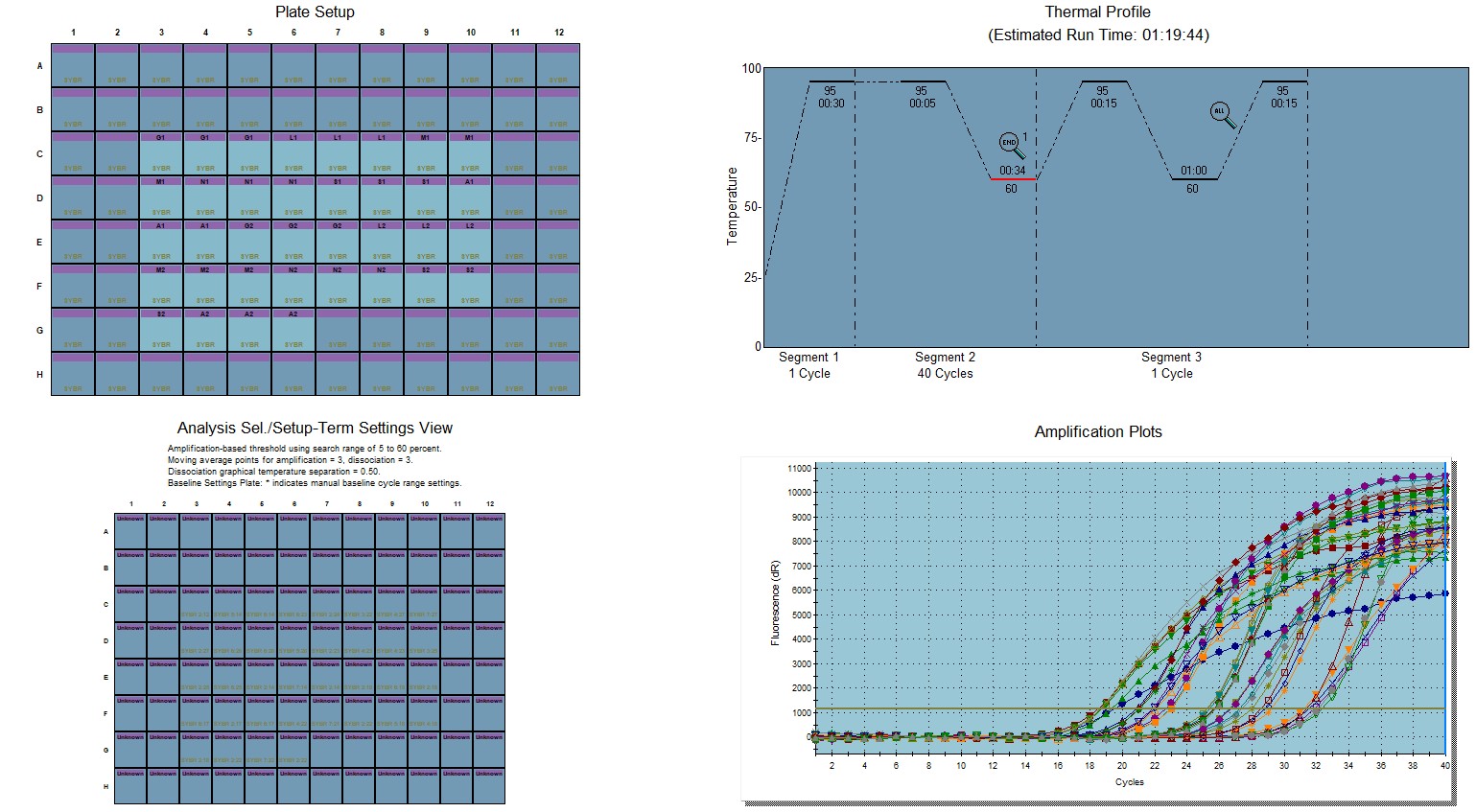

Supplement: Supplementary file 1 [file DataSheet_1.zip › excel+p/13.jpg]

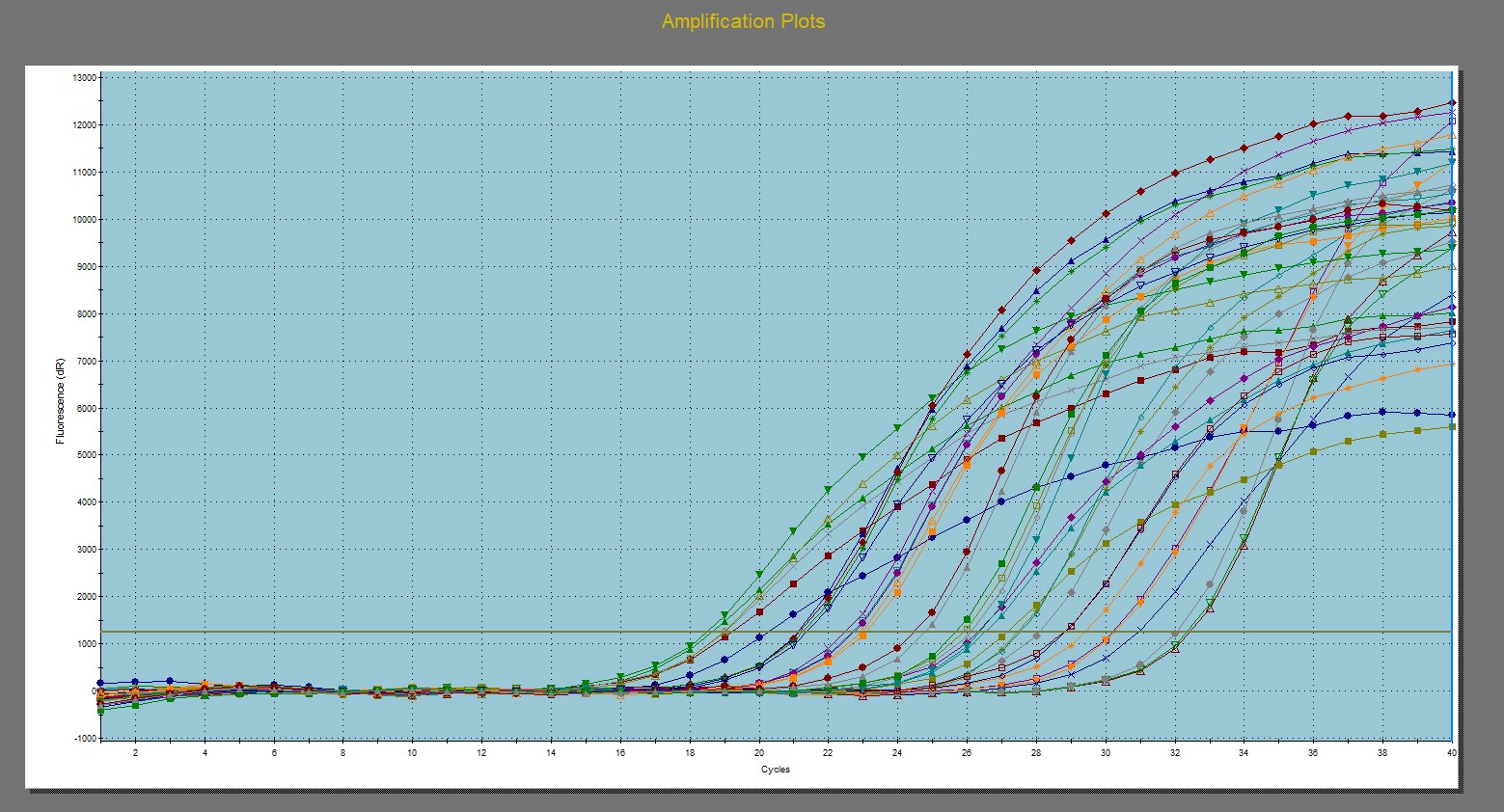

Supplement: Supplementary file 1 [file DataSheet_1.zip › excel+p/14-1.jpg]

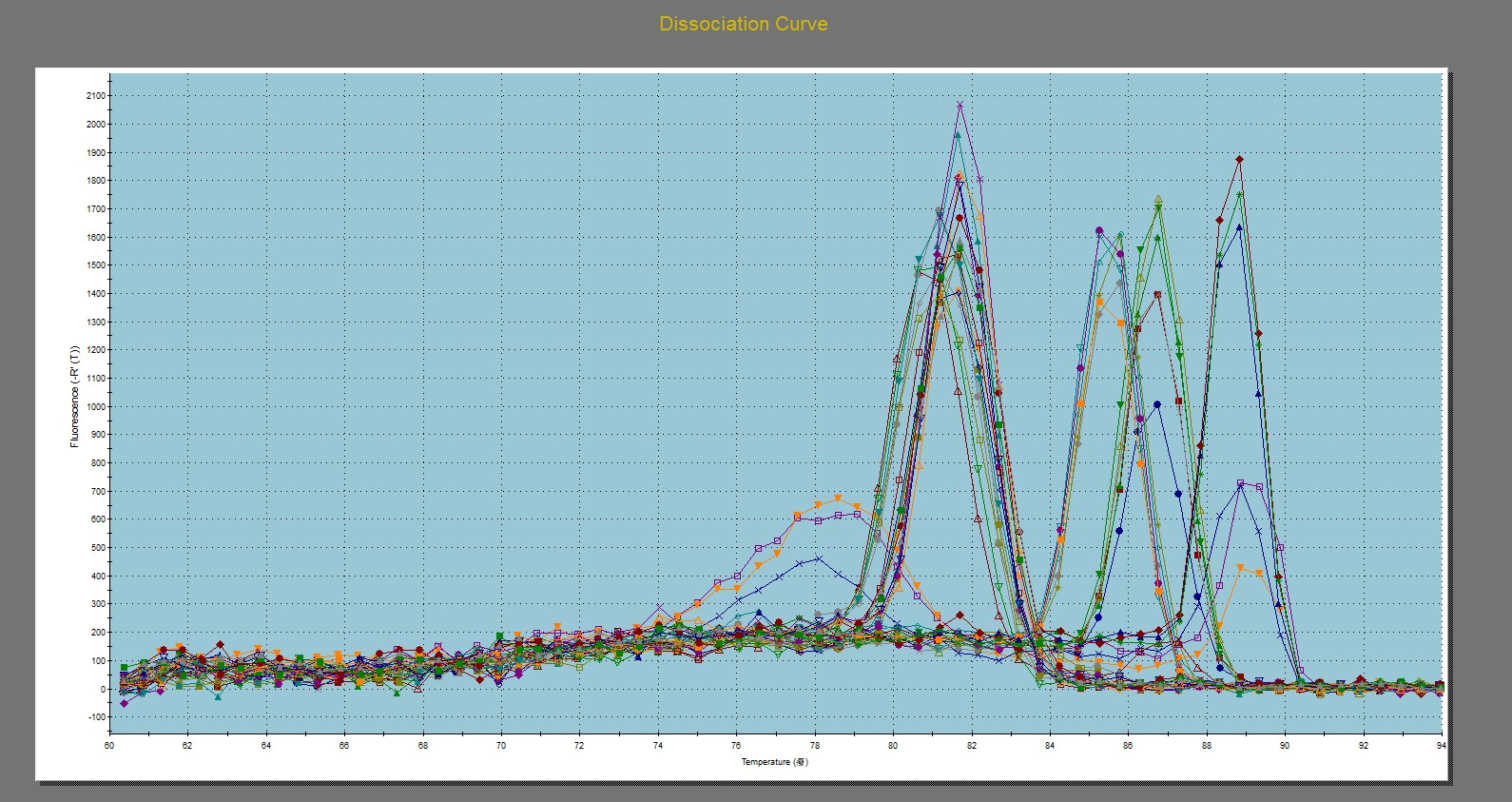

Supplement: Supplementary file 1 [file DataSheet_1.zip › excel+p/14-2.jpg]

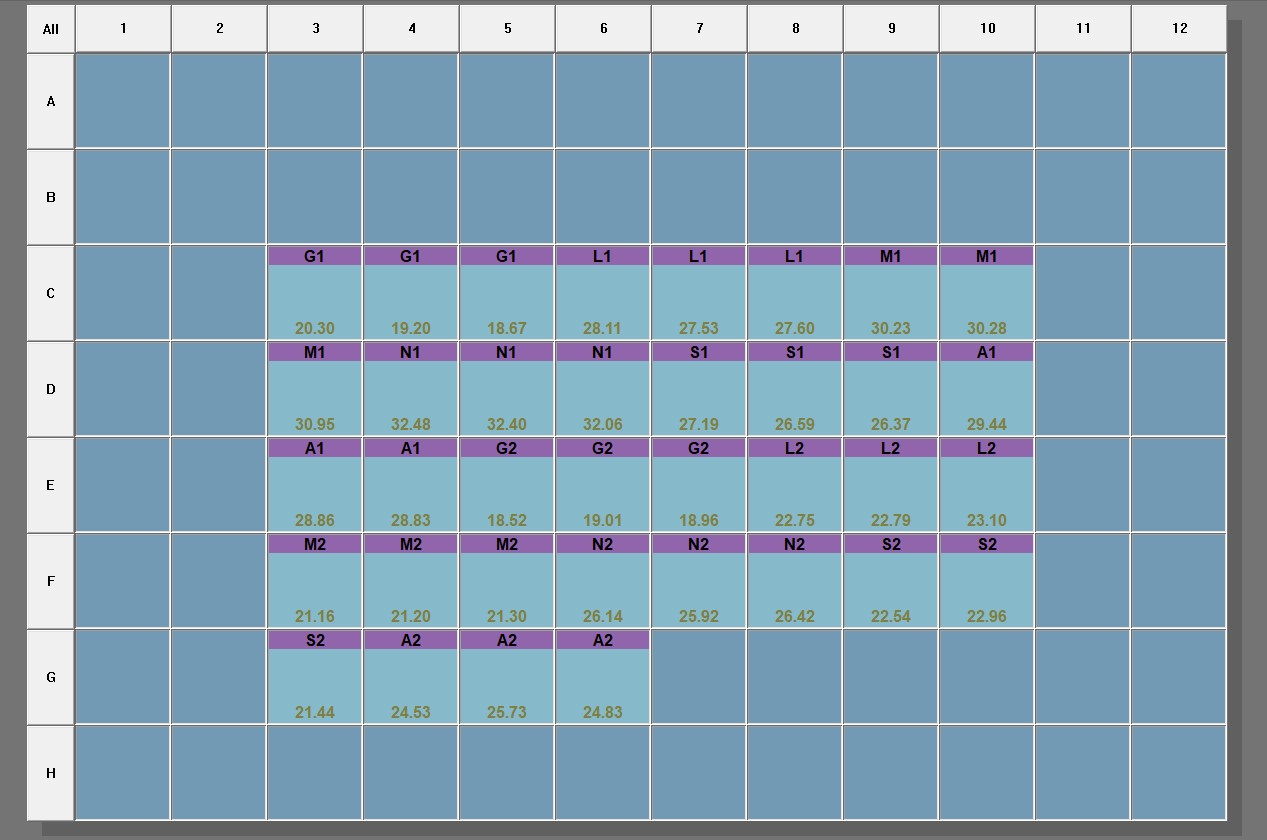

Supplement: Supplementary file 1 [file DataSheet_1.zip › excel+p/14-3.jpg]

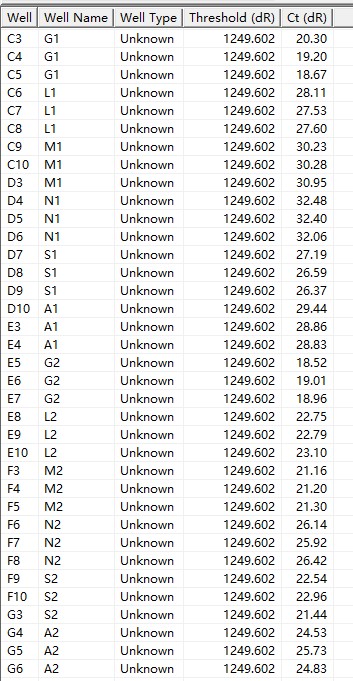

Supplement: Supplementary file 1 [file DataSheet_1.zip › excel+p/14-4.jpg]

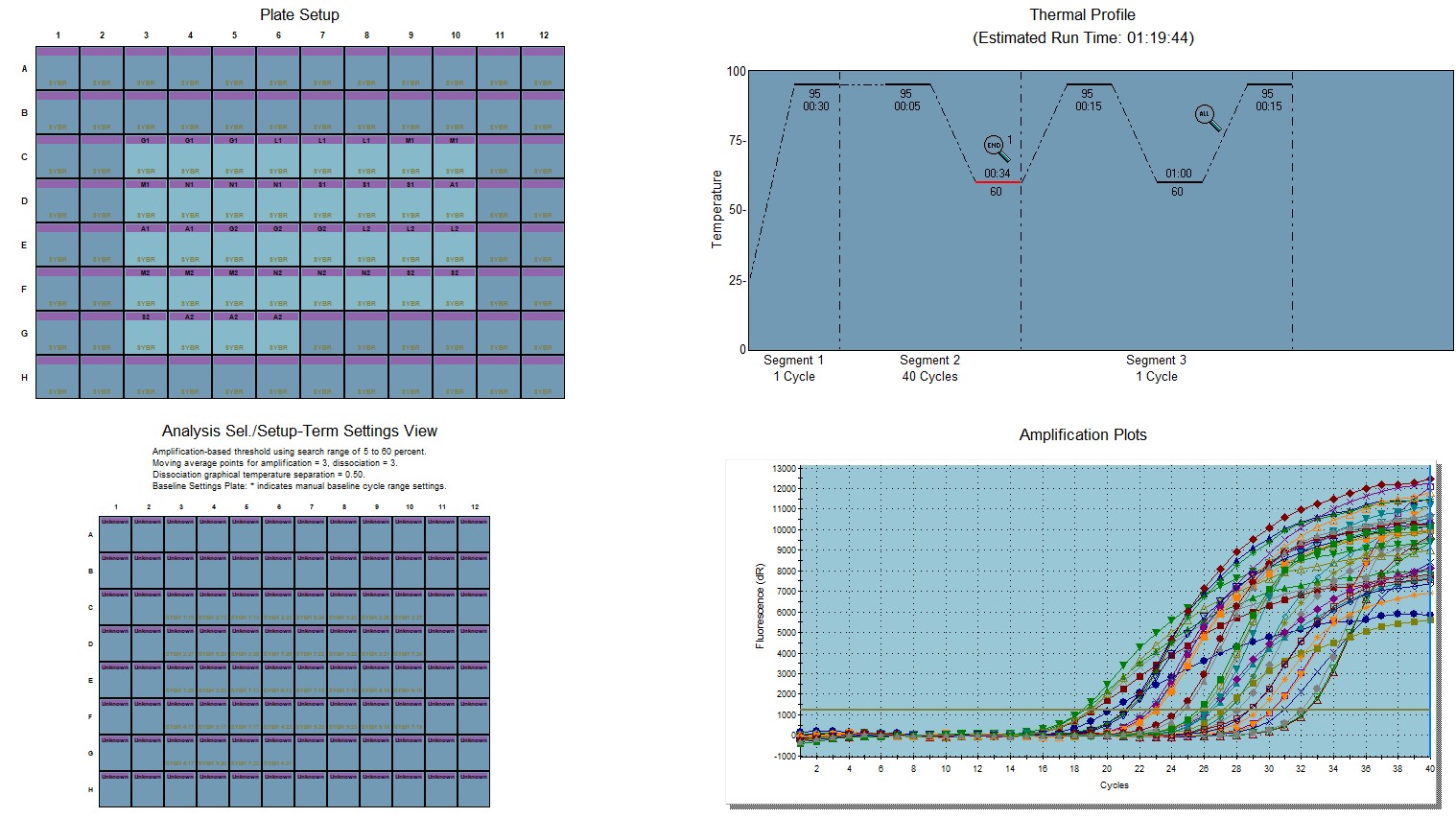

Supplement: Supplementary file 1 [file DataSheet_1.zip › excel+p/14.jpg]

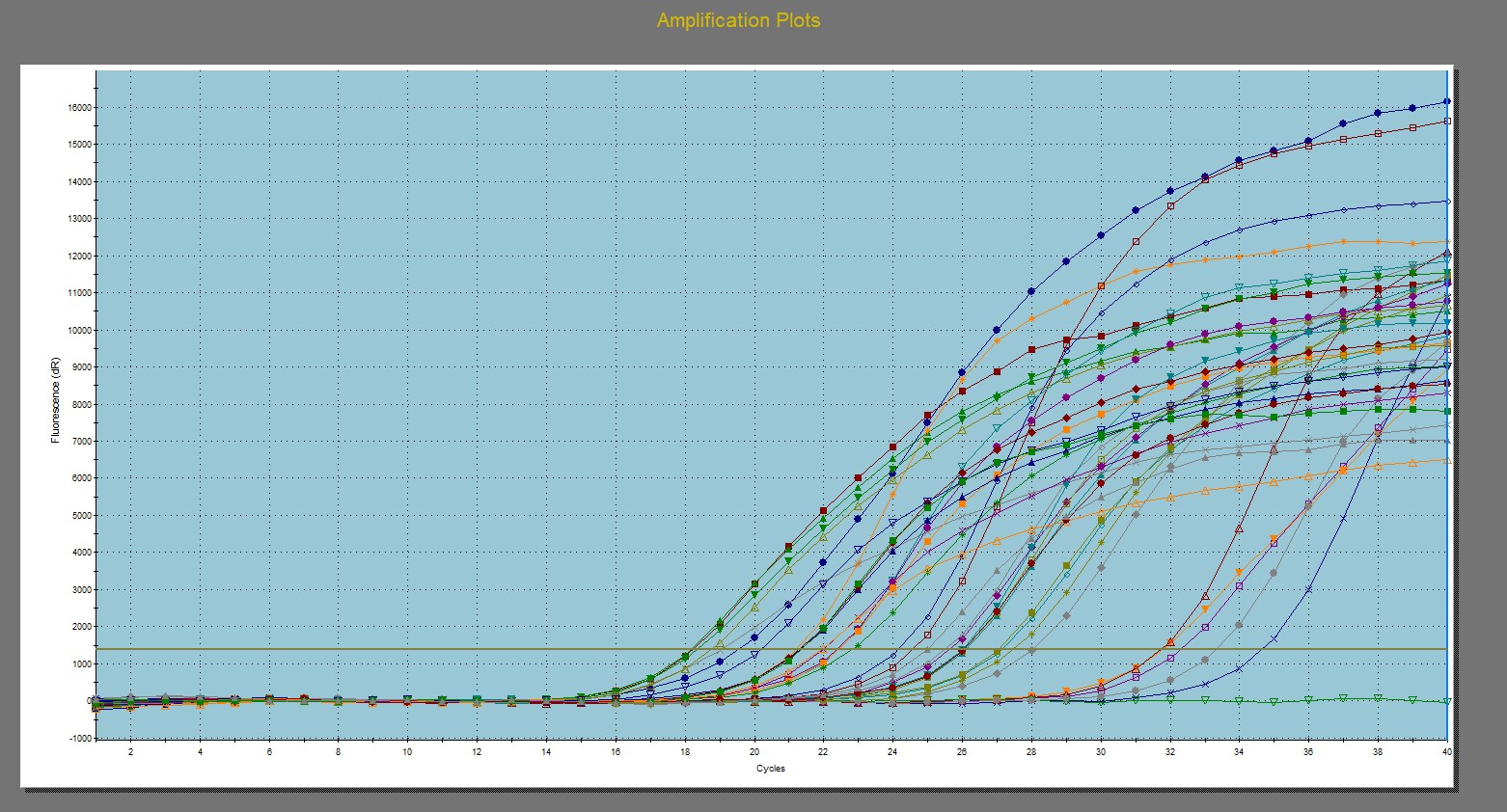

Supplement: Supplementary file 1 [file DataSheet_1.zip › excel+p/15-1.jpg]

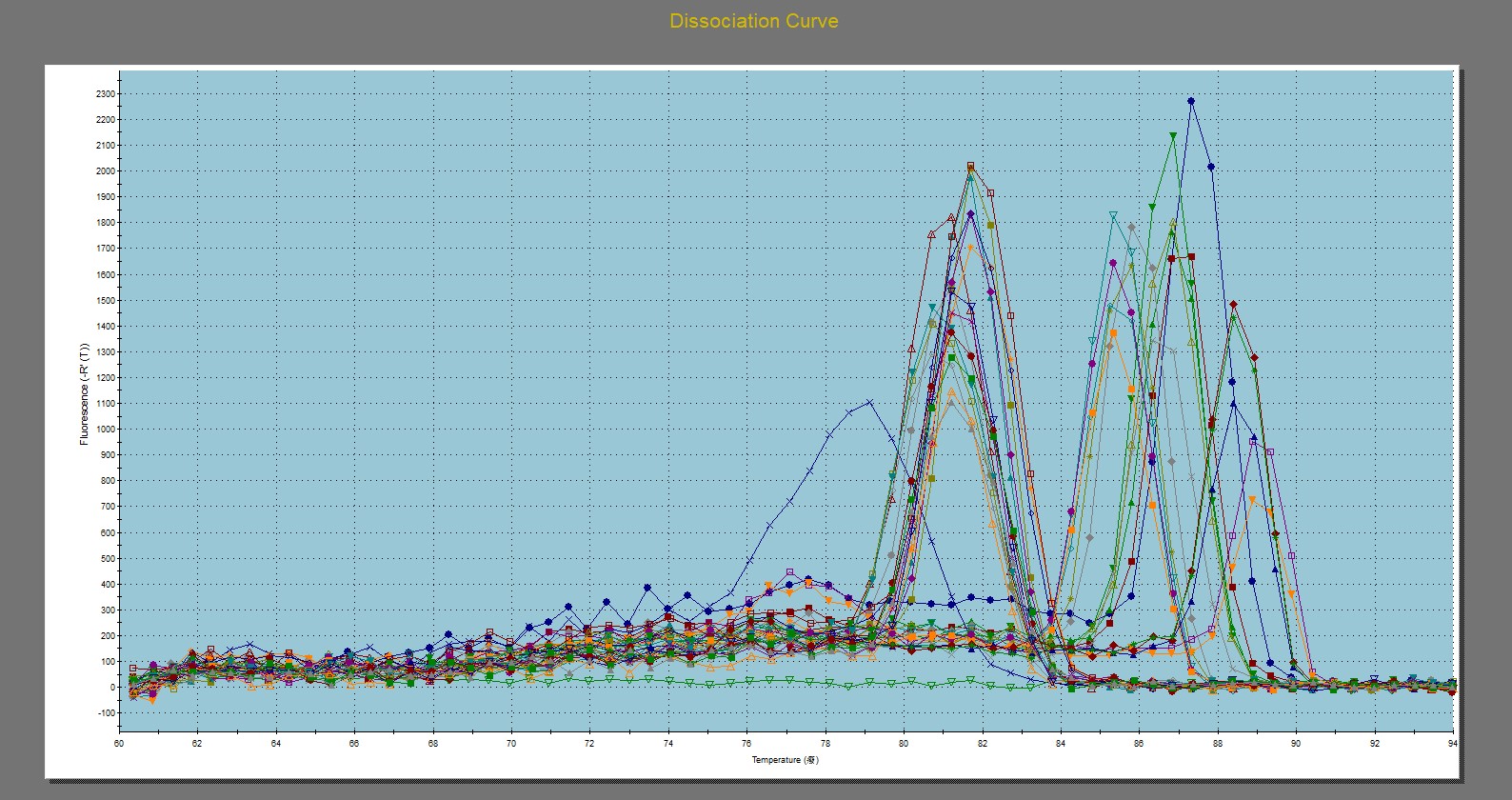

Supplement: Supplementary file 1 [file DataSheet_1.zip › excel+p/15-2.jpg]

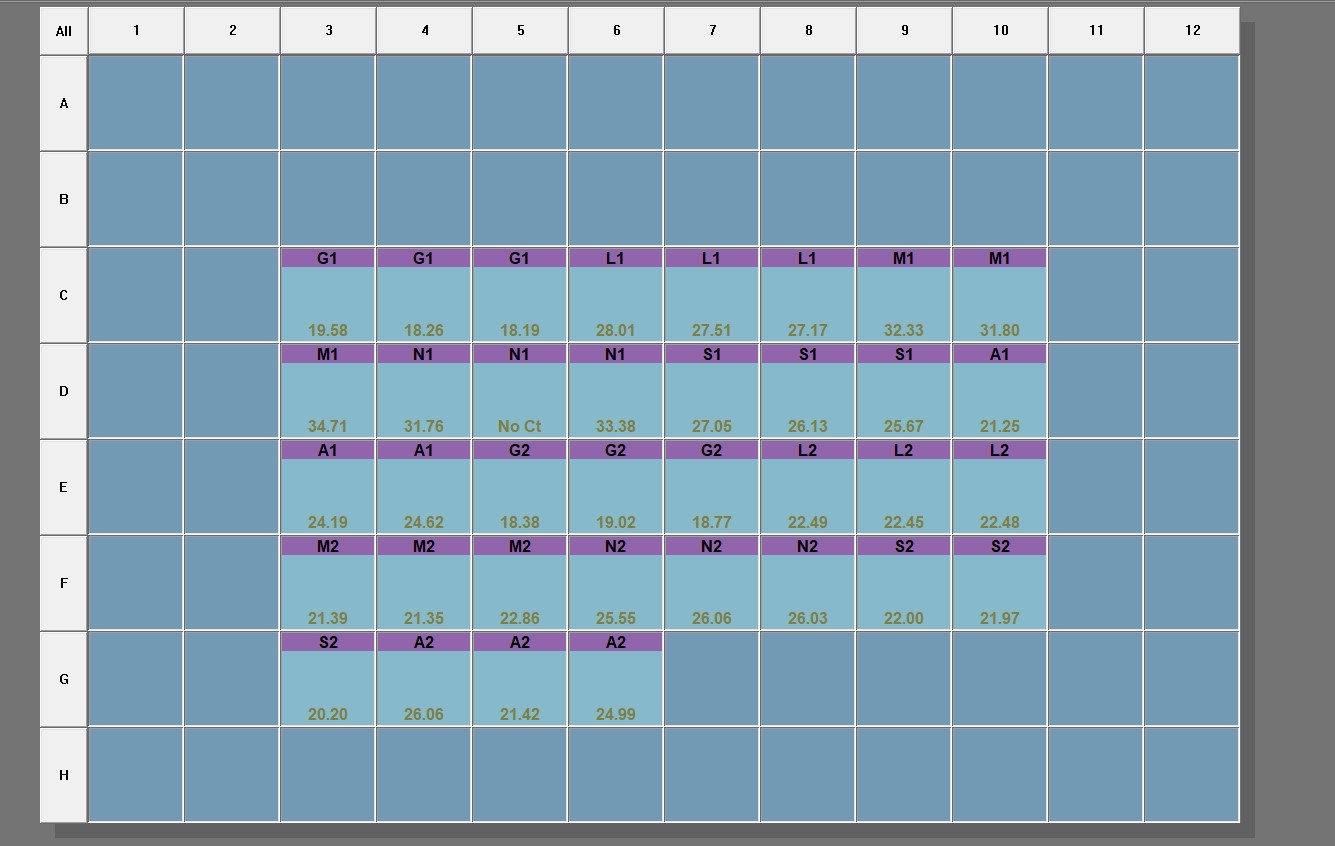

Supplement: Supplementary file 1 [file DataSheet_1.zip › excel+p/15-3.jpg]

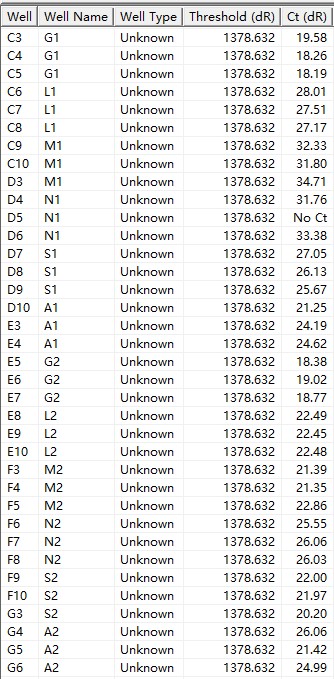

Supplement: Supplementary file 1 [file DataSheet_1.zip › excel+p/15-4.jpg]

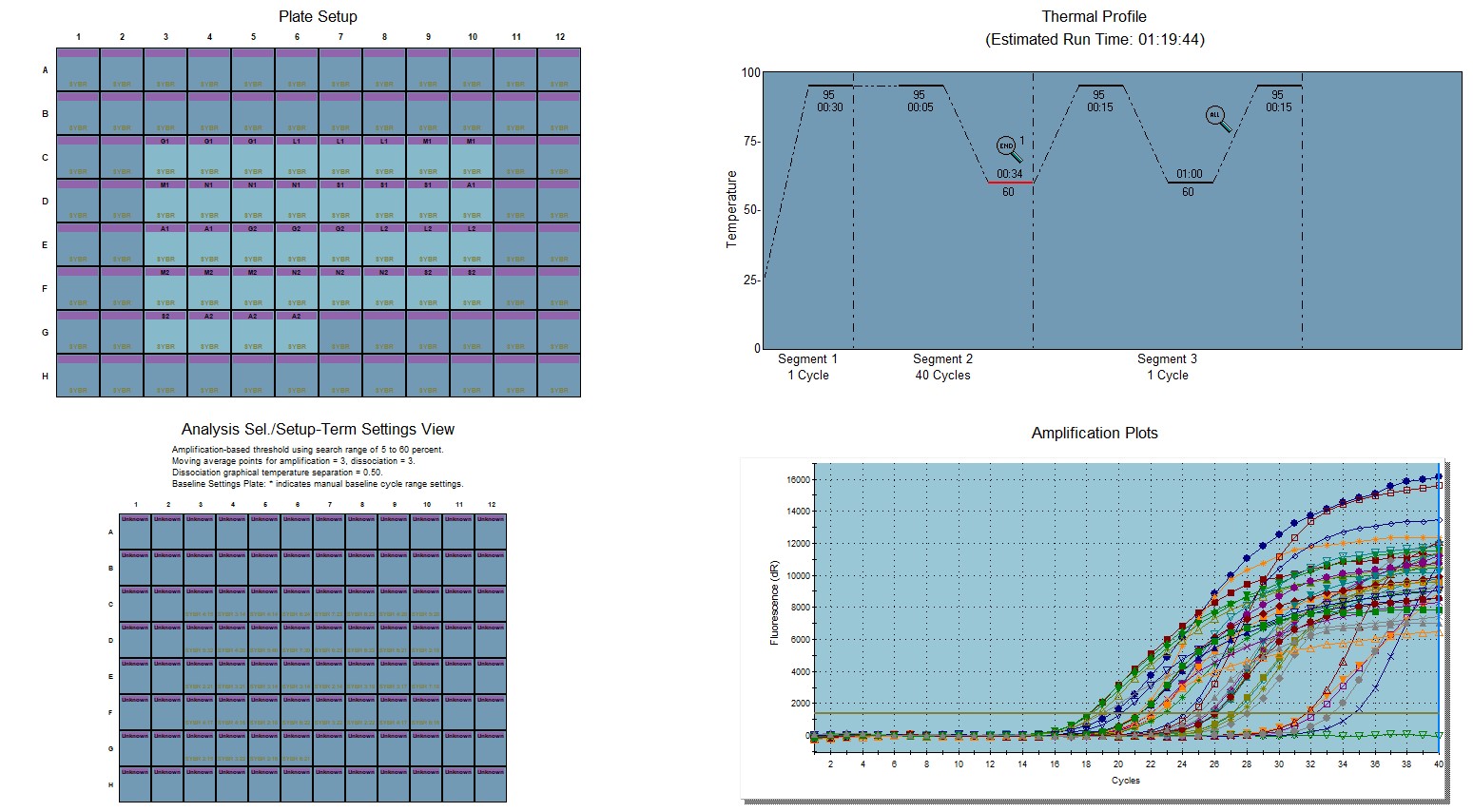

Supplement: Supplementary file 1 [file DataSheet_1.zip › excel+p/15.jpg]

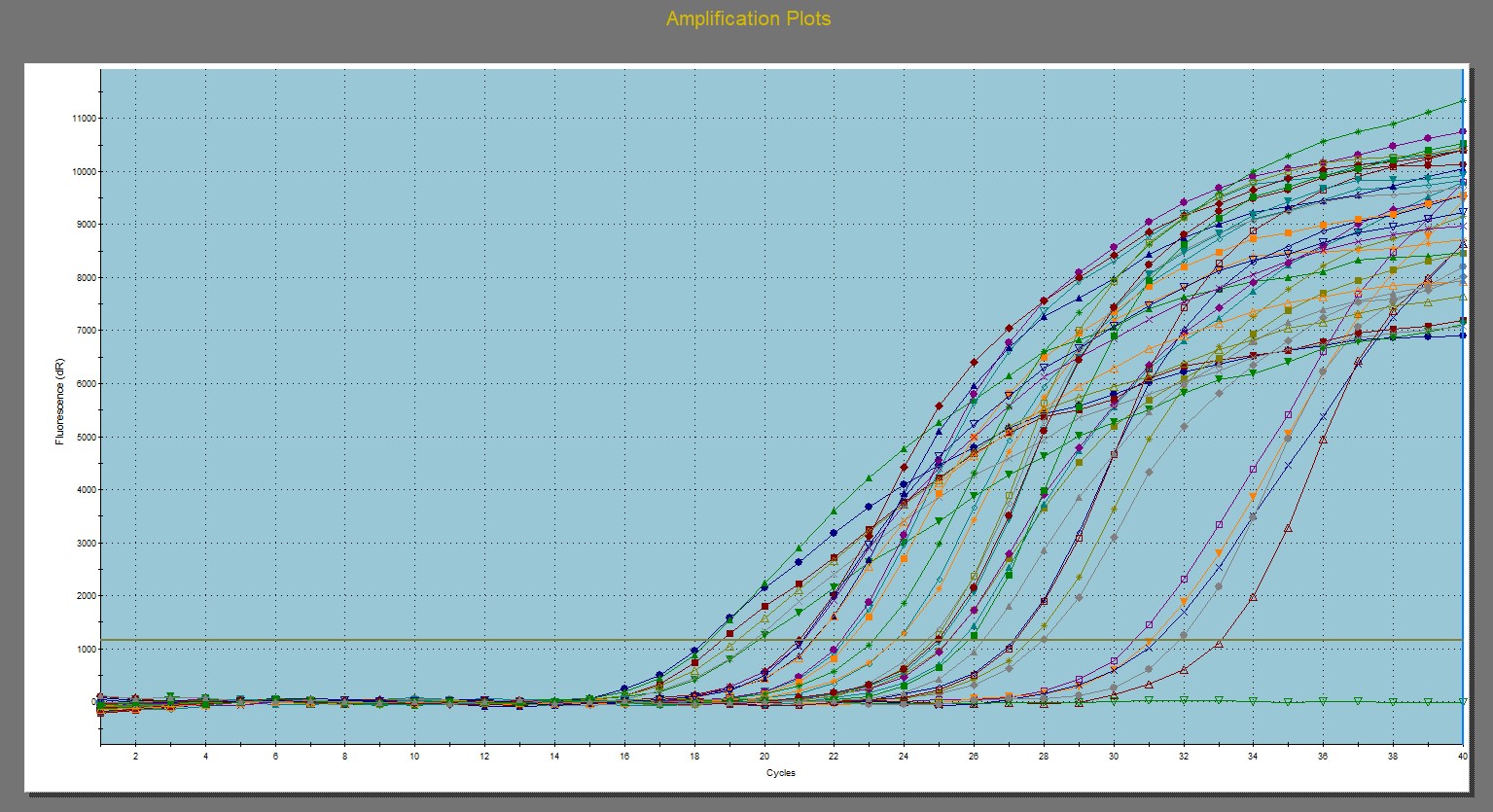

Supplement: Supplementary file 1 [file DataSheet_1.zip › excel+p/16-1.jpg]

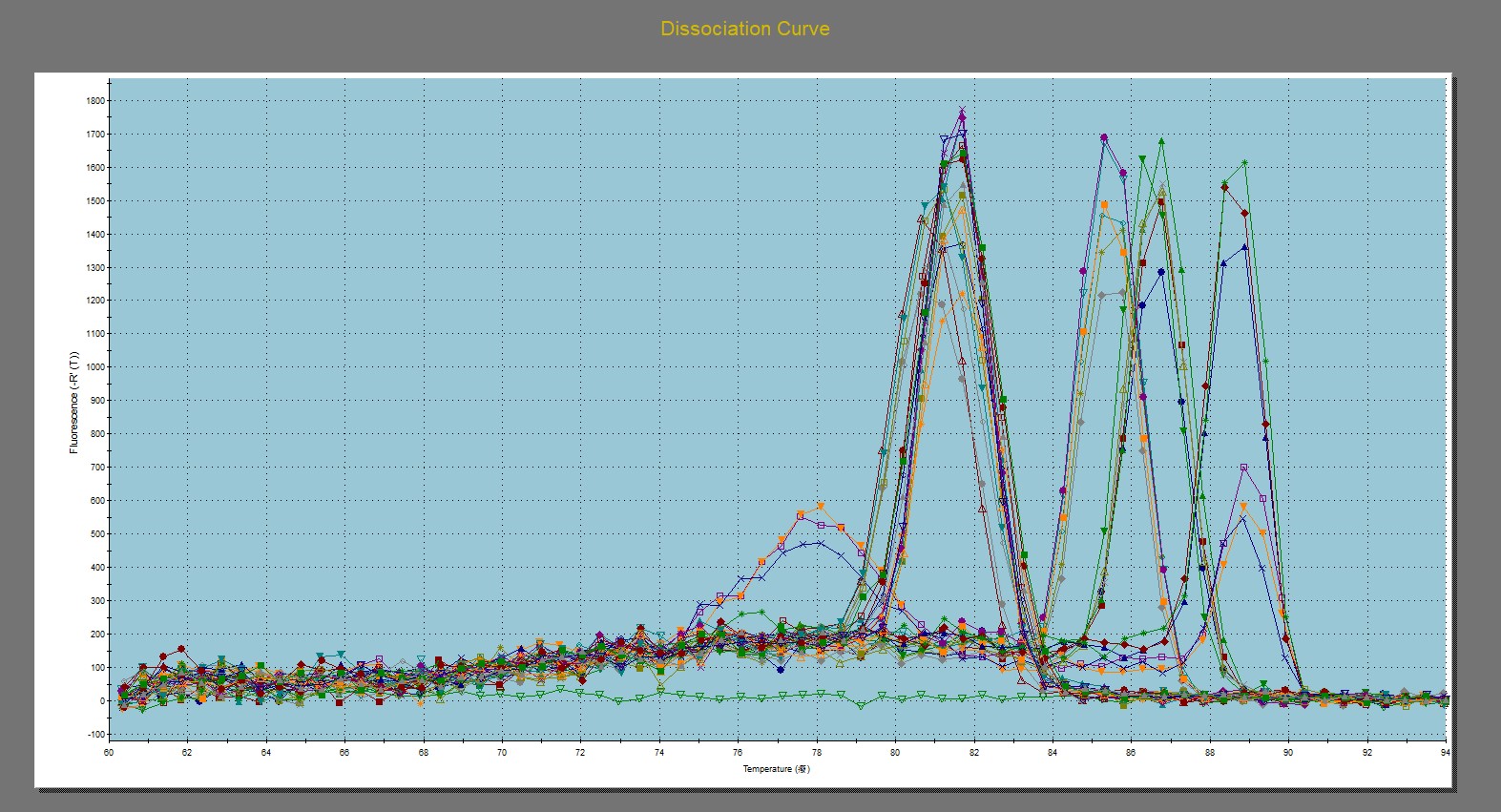

Supplement: Supplementary file 1 [file DataSheet_1.zip › excel+p/16-2.jpg]

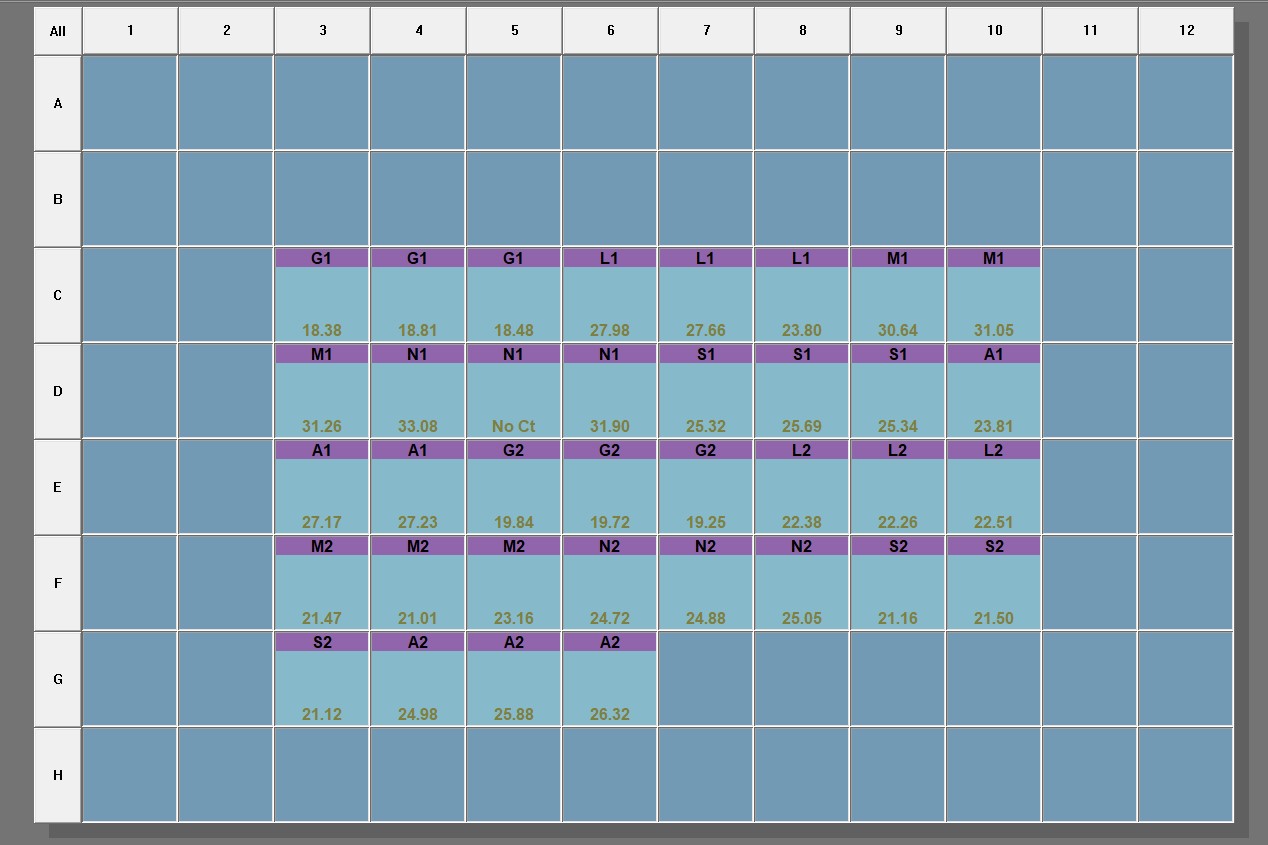

Supplement: Supplementary file 1 [file DataSheet_1.zip › excel+p/16-3.jpg]

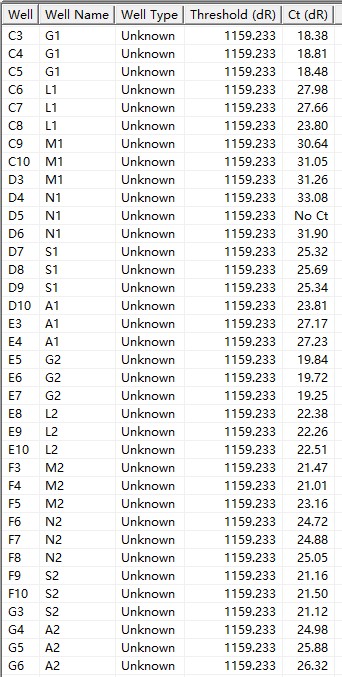

Supplement: Supplementary file 1 [file DataSheet_1.zip › excel+p/16-4.jpg]

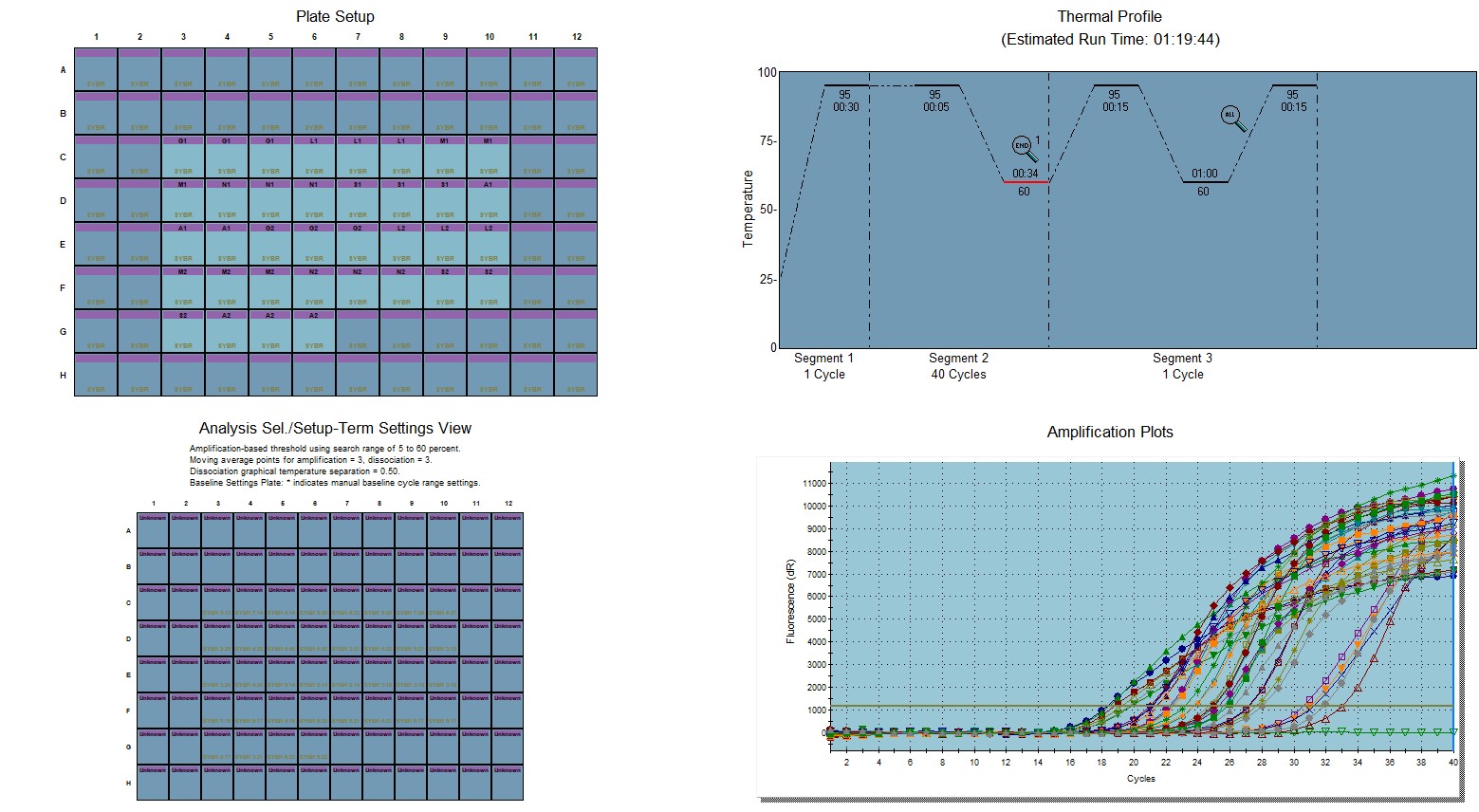

Supplement: Supplementary file 1 [file DataSheet_1.zip › excel+p/16.jpg]

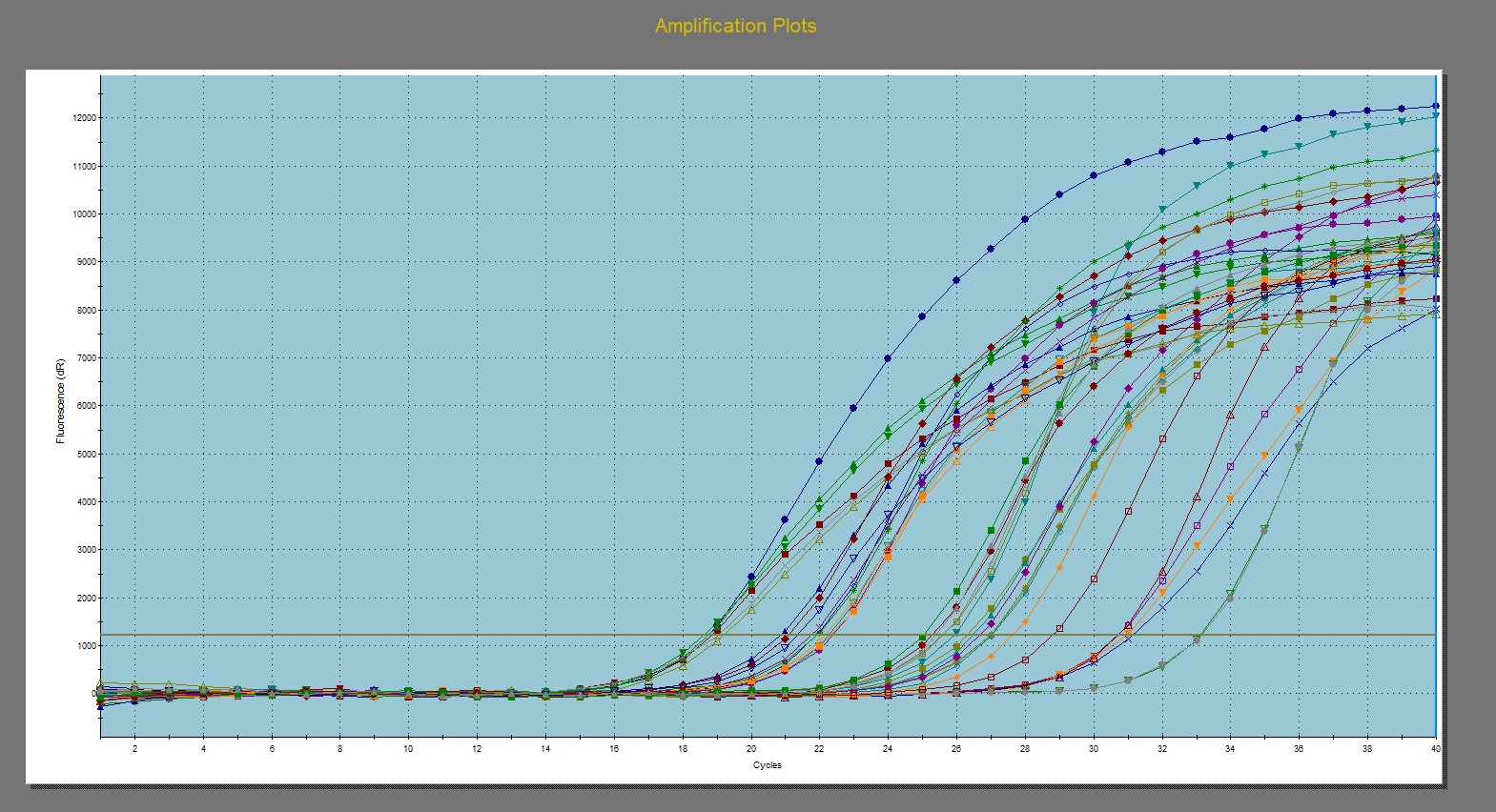

Supplement: Supplementary file 1 [file DataSheet_1.zip › excel+p/17-1.jpg]

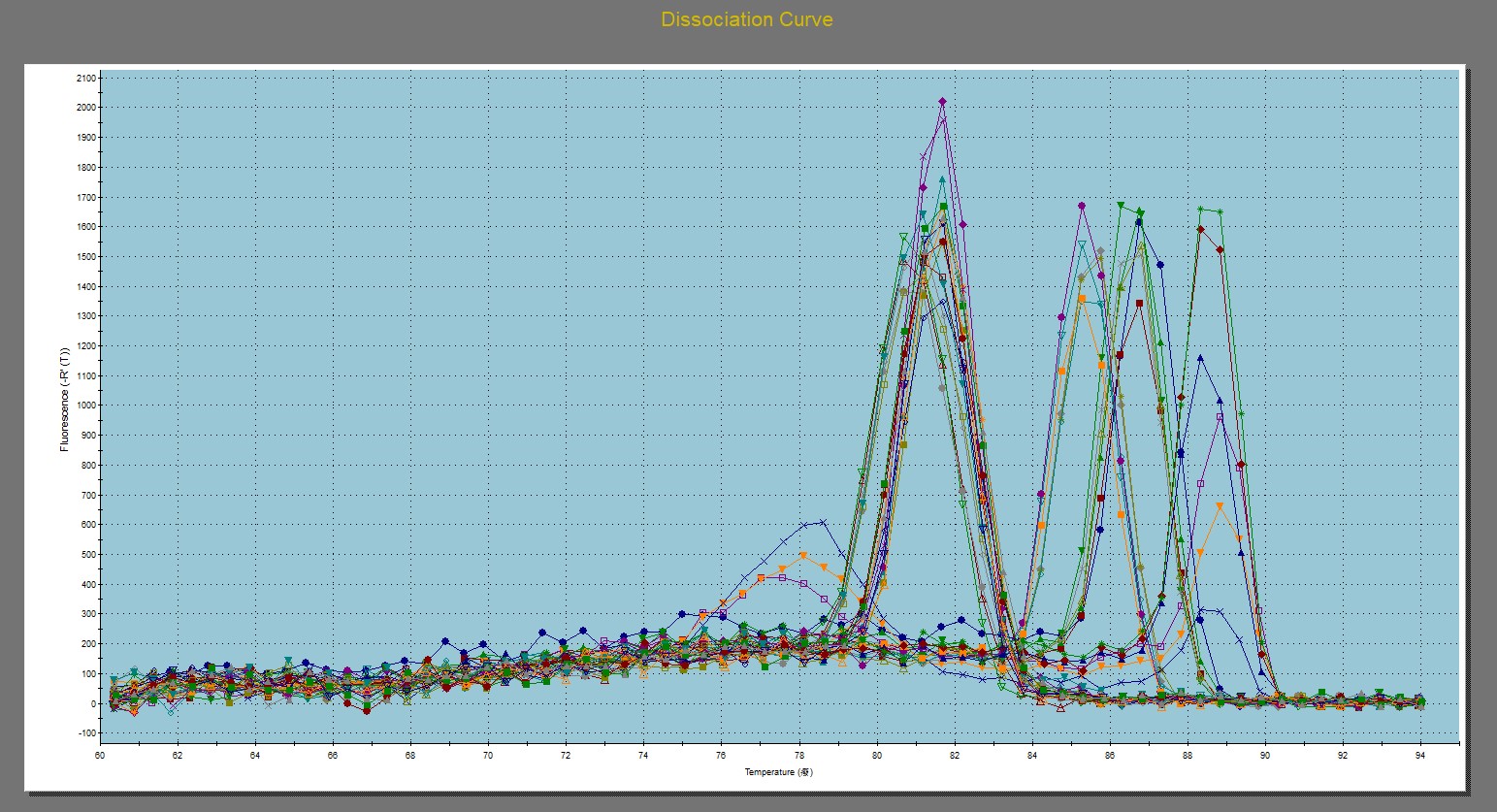

Supplement: Supplementary file 1 [file DataSheet_1.zip › excel+p/17-2.jpg]

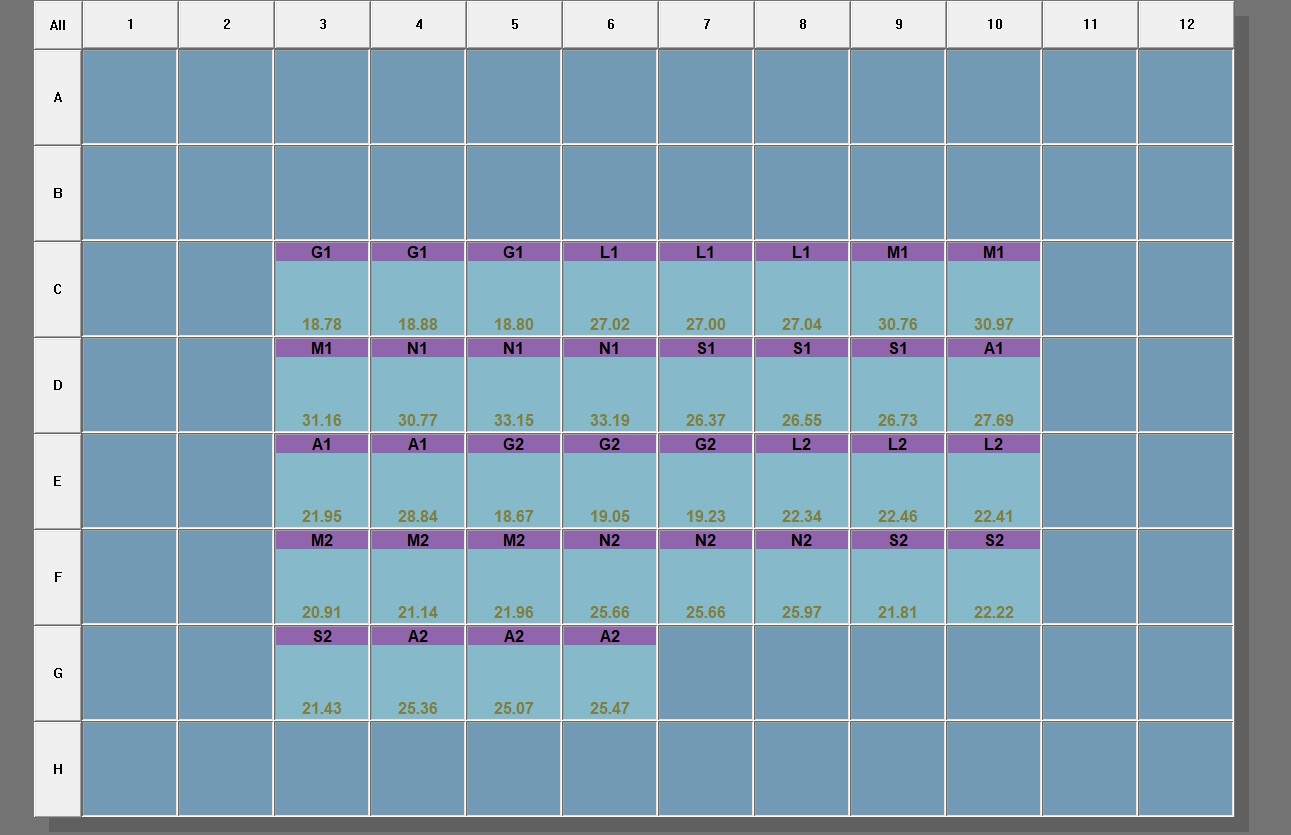

Supplement: Supplementary file 1 [file DataSheet_1.zip › excel+p/17-3.jpg]

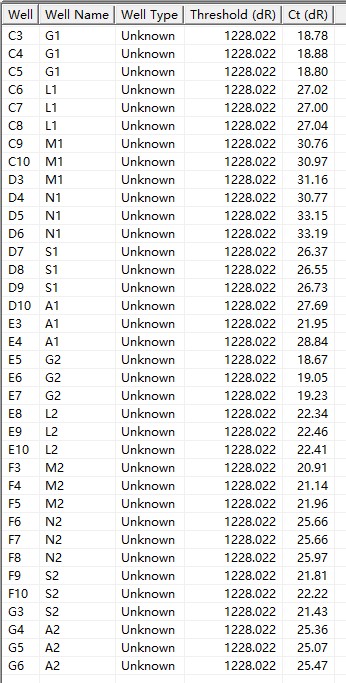

Supplement: Supplementary file 1 [file DataSheet_1.zip › excel+p/17-4.jpg]

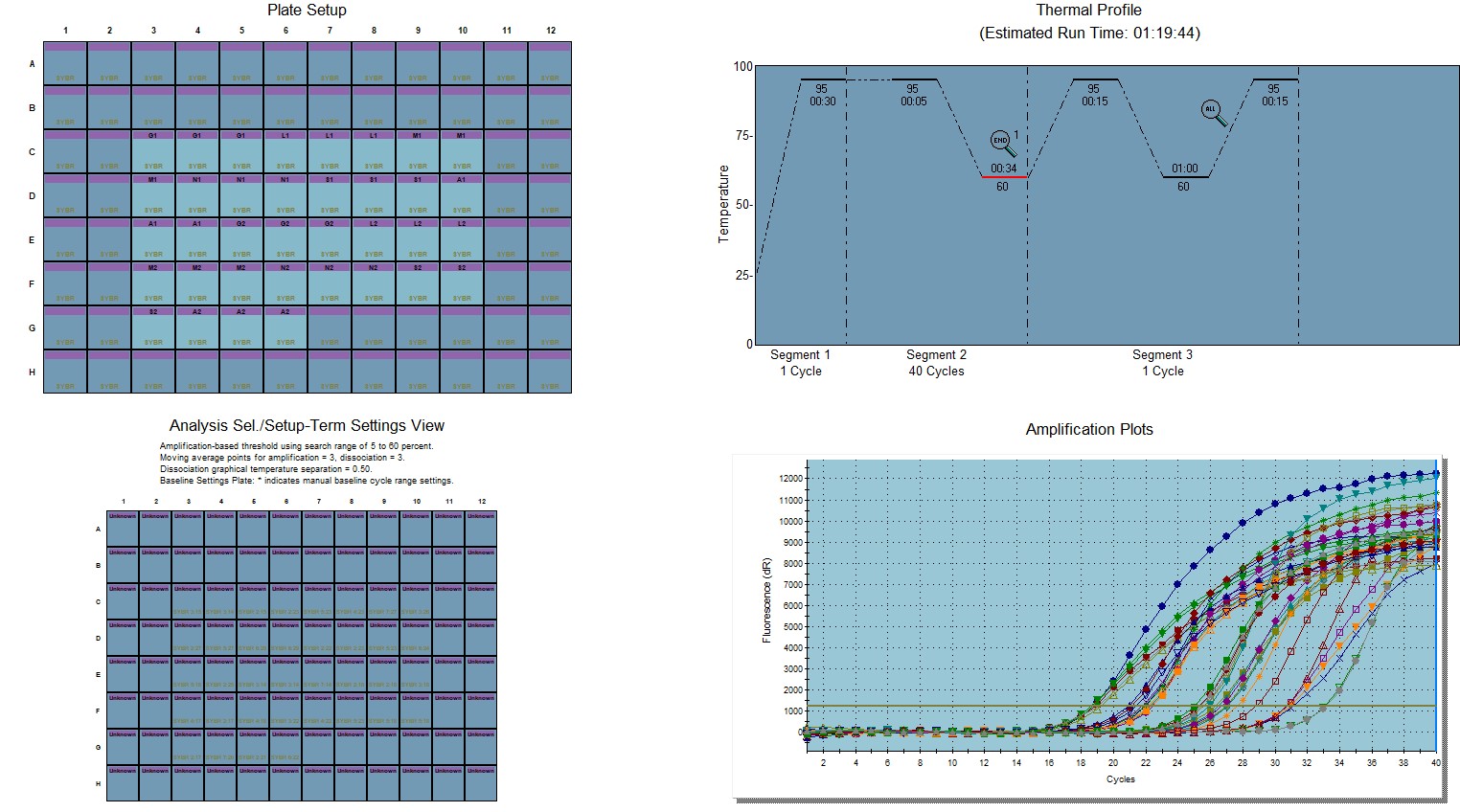

Supplement: Supplementary file 1 [file DataSheet_1.zip › excel+p/17.jpg]

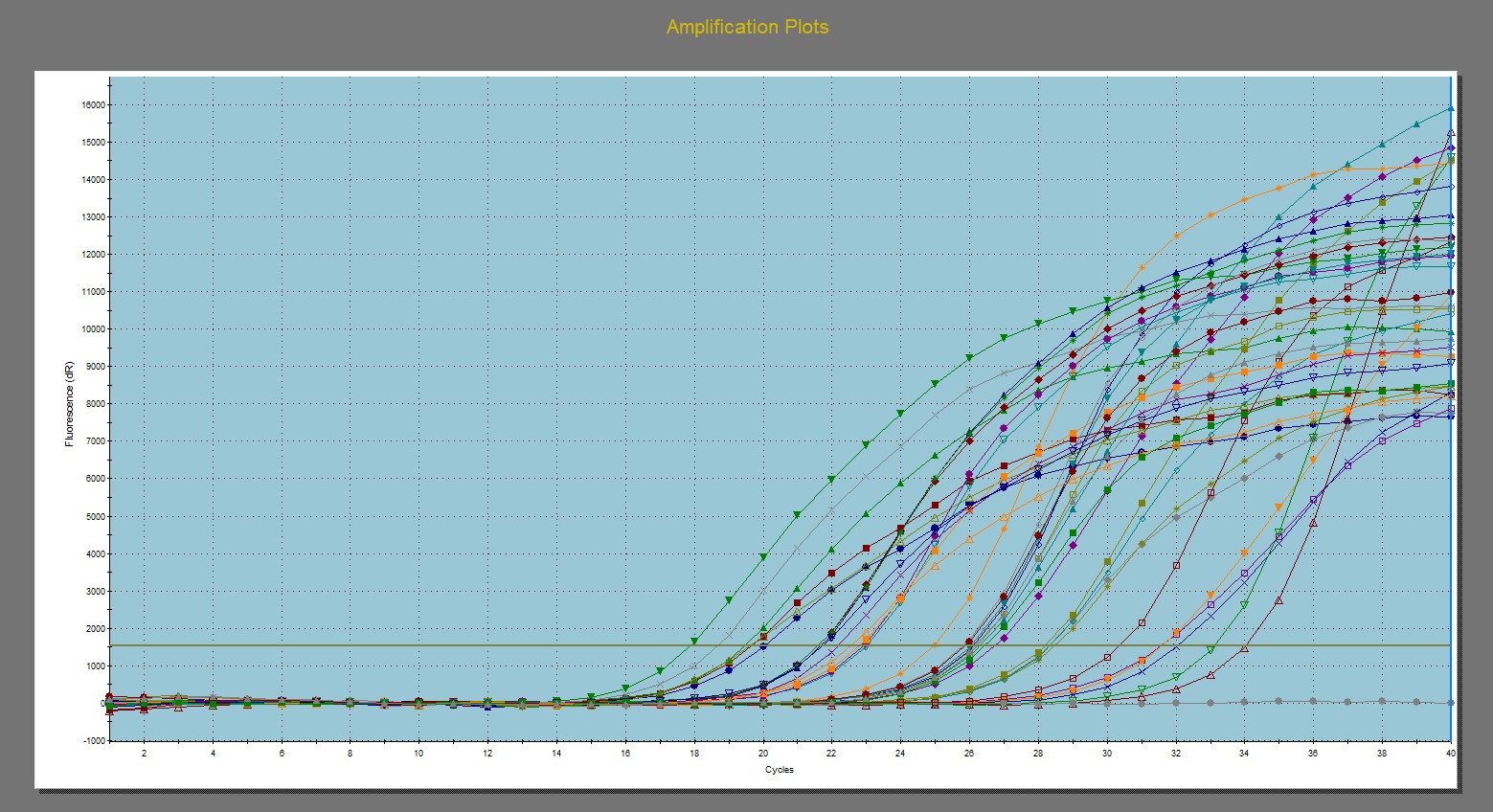

Supplement: Supplementary file 1 [file DataSheet_1.zip › excel+p/18-1.jpg]

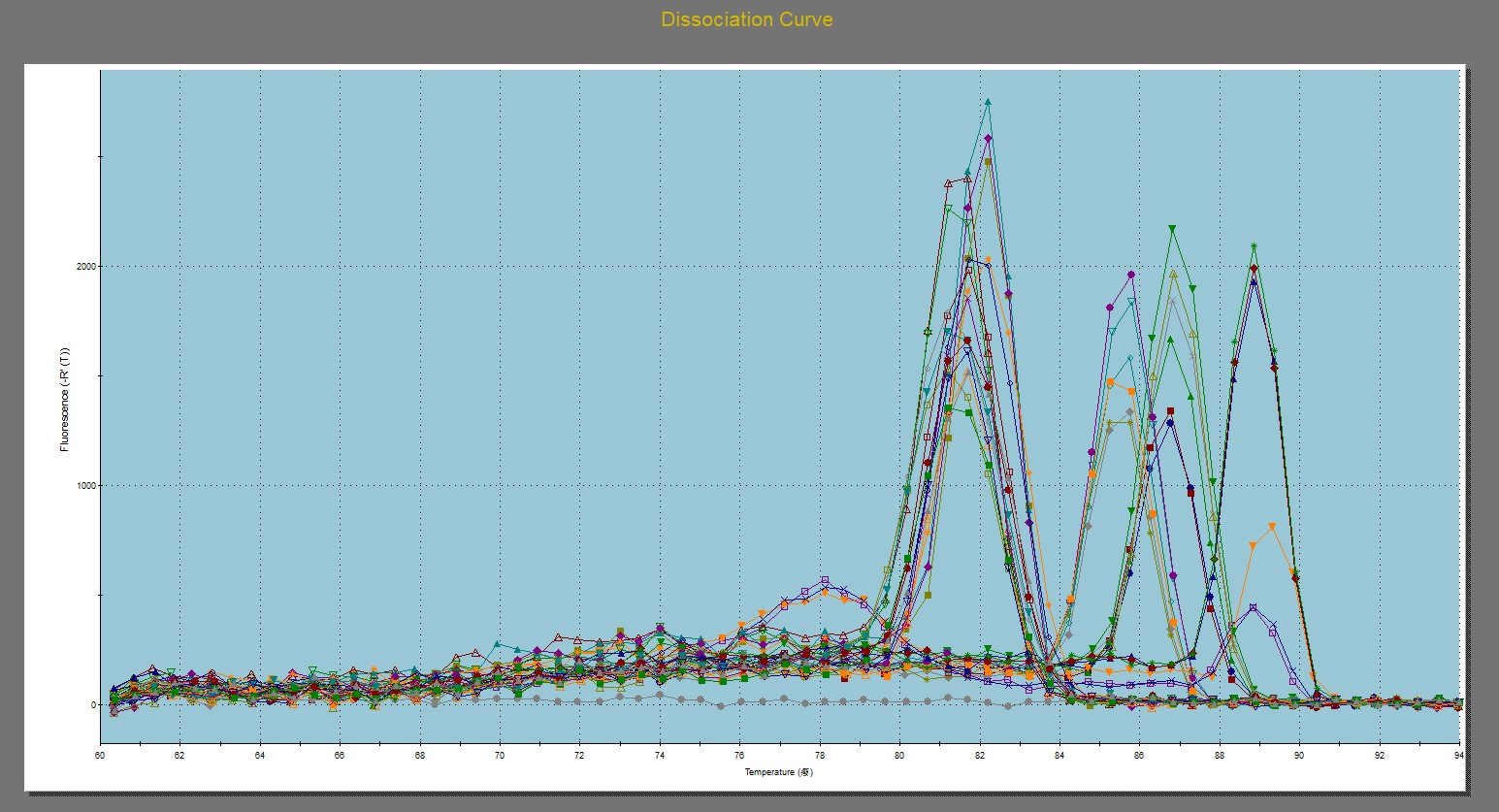

Supplement: Supplementary file 1 [file DataSheet_1.zip › excel+p/18-2.jpg]

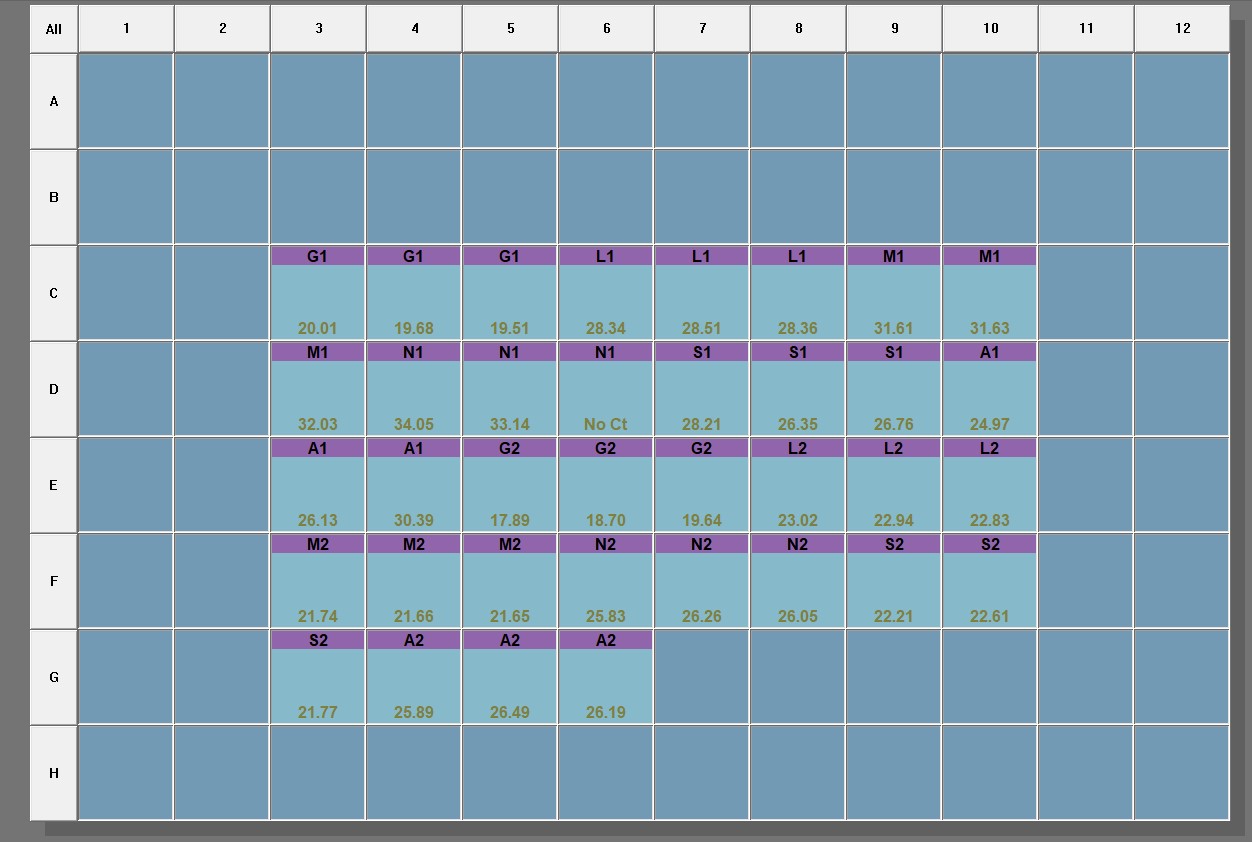

Supplement: Supplementary file 1 [file DataSheet_1.zip › excel+p/18-3.jpg]

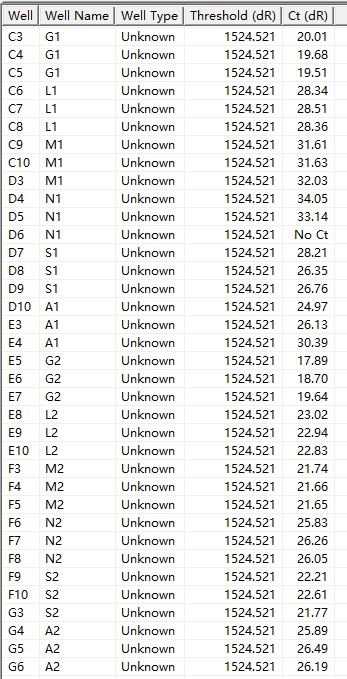

Supplement: Supplementary file 1 [file DataSheet_1.zip › excel+p/18-4.jpg]

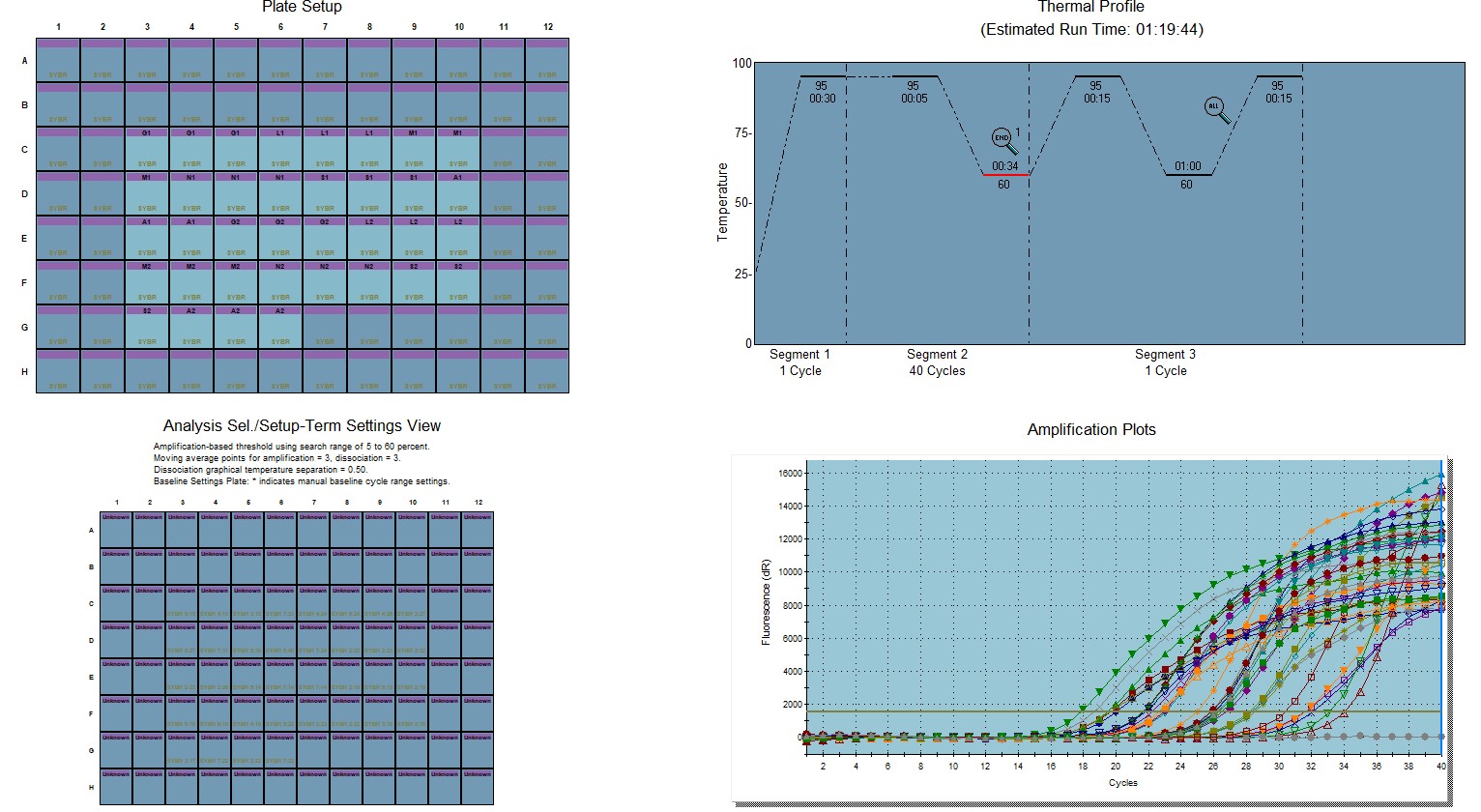

Supplement: Supplementary file 1 [file DataSheet_1.zip › excel+p/18.jpg]

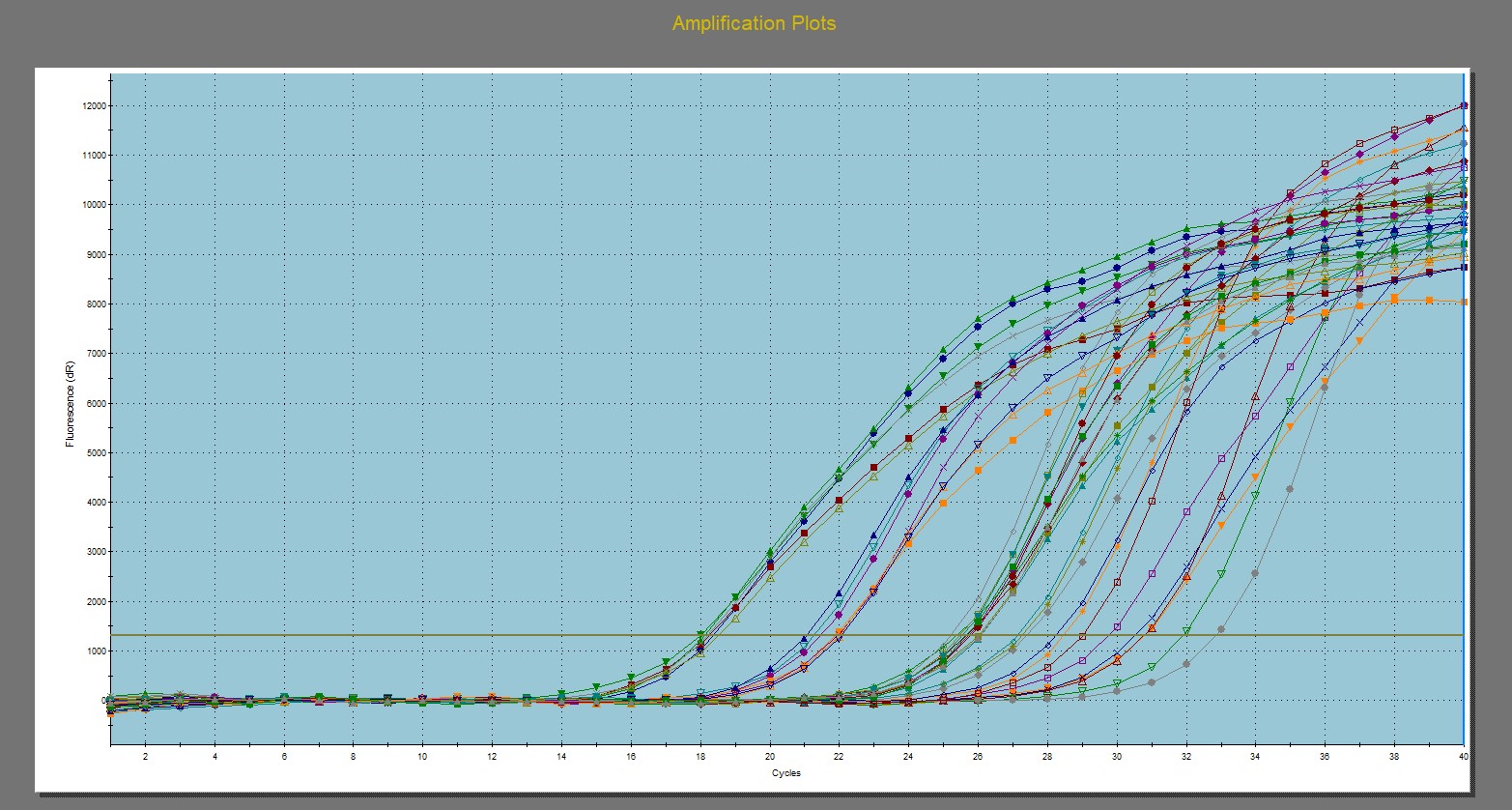

Supplement: Supplementary file 1 [file DataSheet_1.zip › excel+p/19-1.jpg]

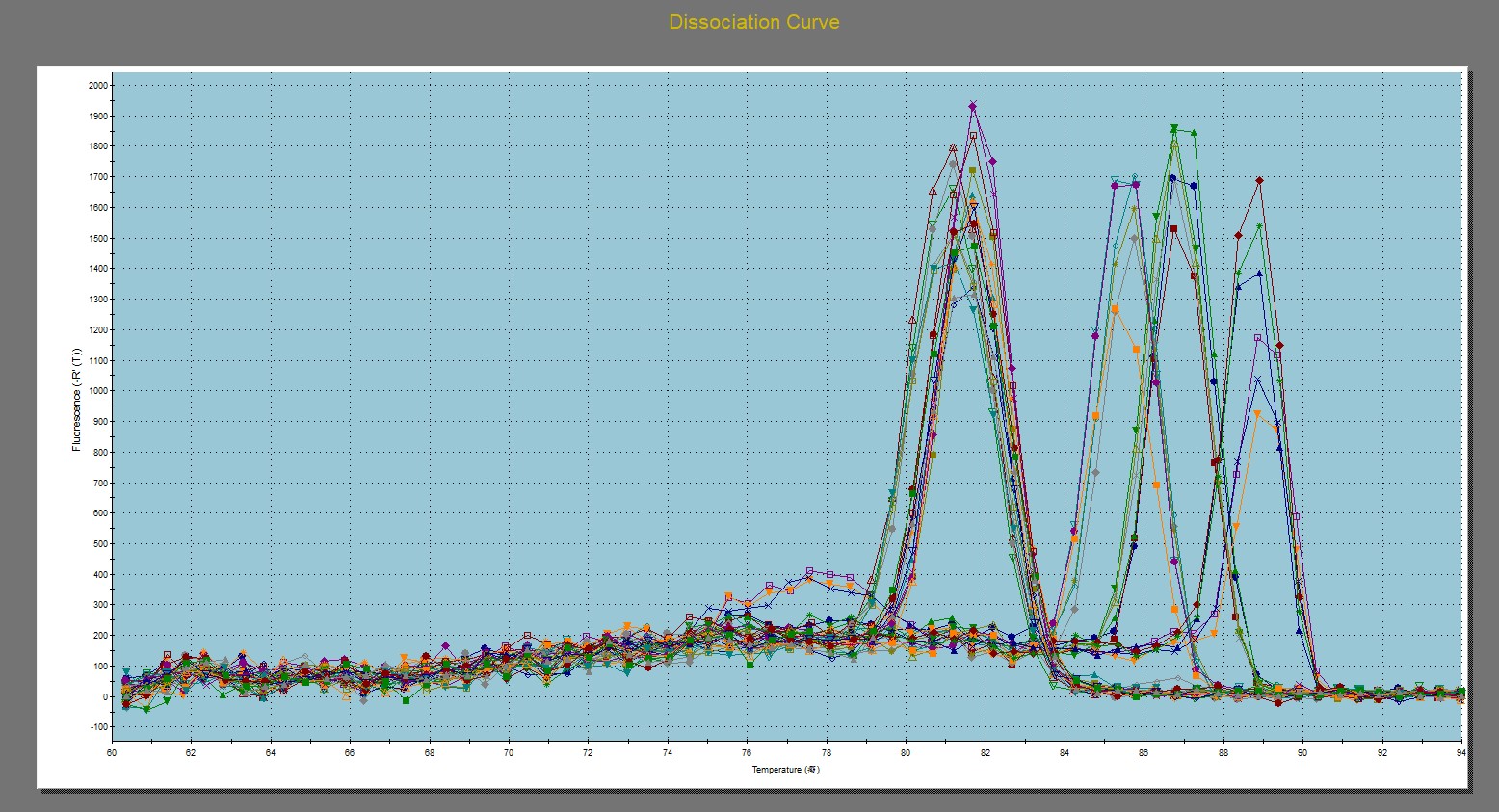

Supplement: Supplementary file 1 [file DataSheet_1.zip › excel+p/19-2.jpg]

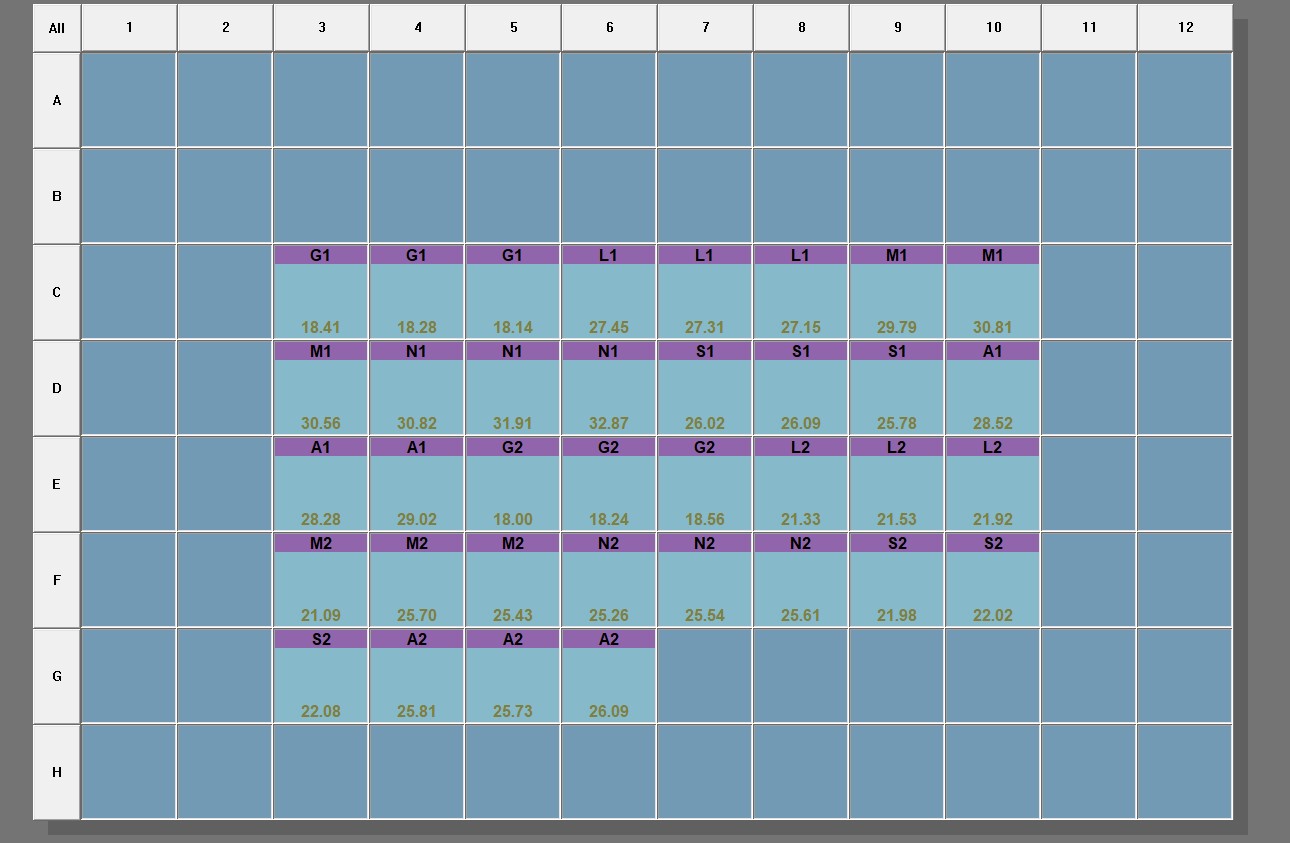

Supplement: Supplementary file 1 [file DataSheet_1.zip › excel+p/19-3.jpg]

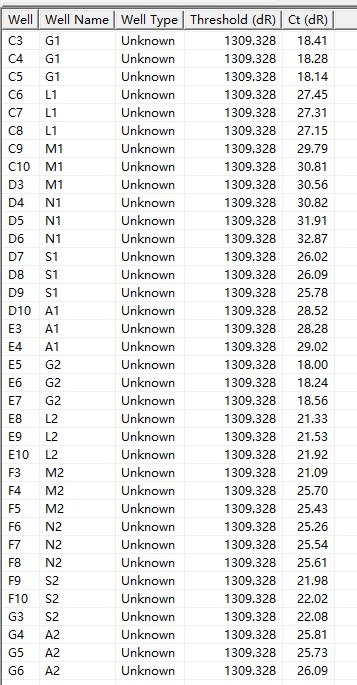

Supplement: Supplementary file 1 [file DataSheet_1.zip › excel+p/19-4.jpg]

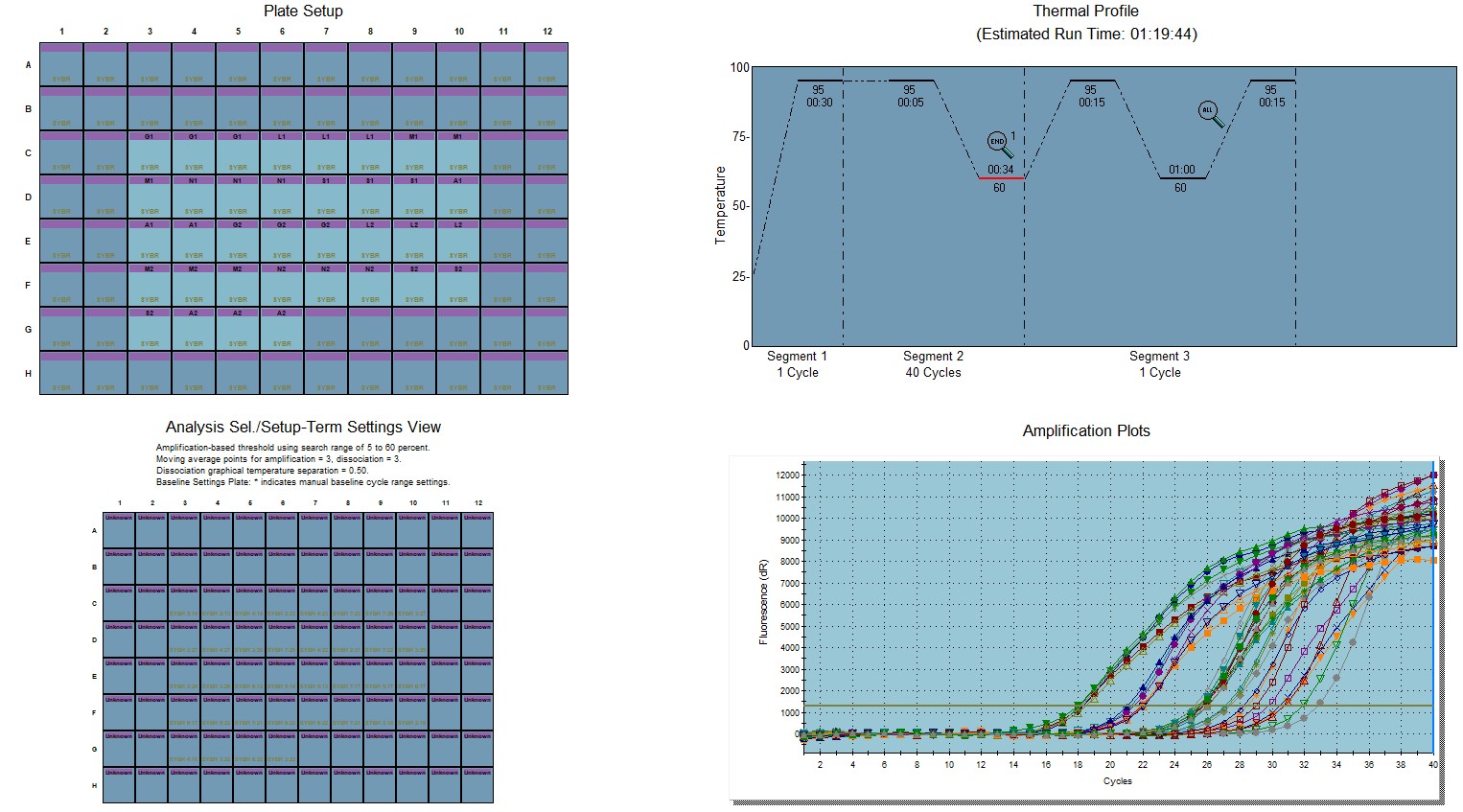

Supplement: Supplementary file 1 [file DataSheet_1.zip › excel+p/19.jpg]

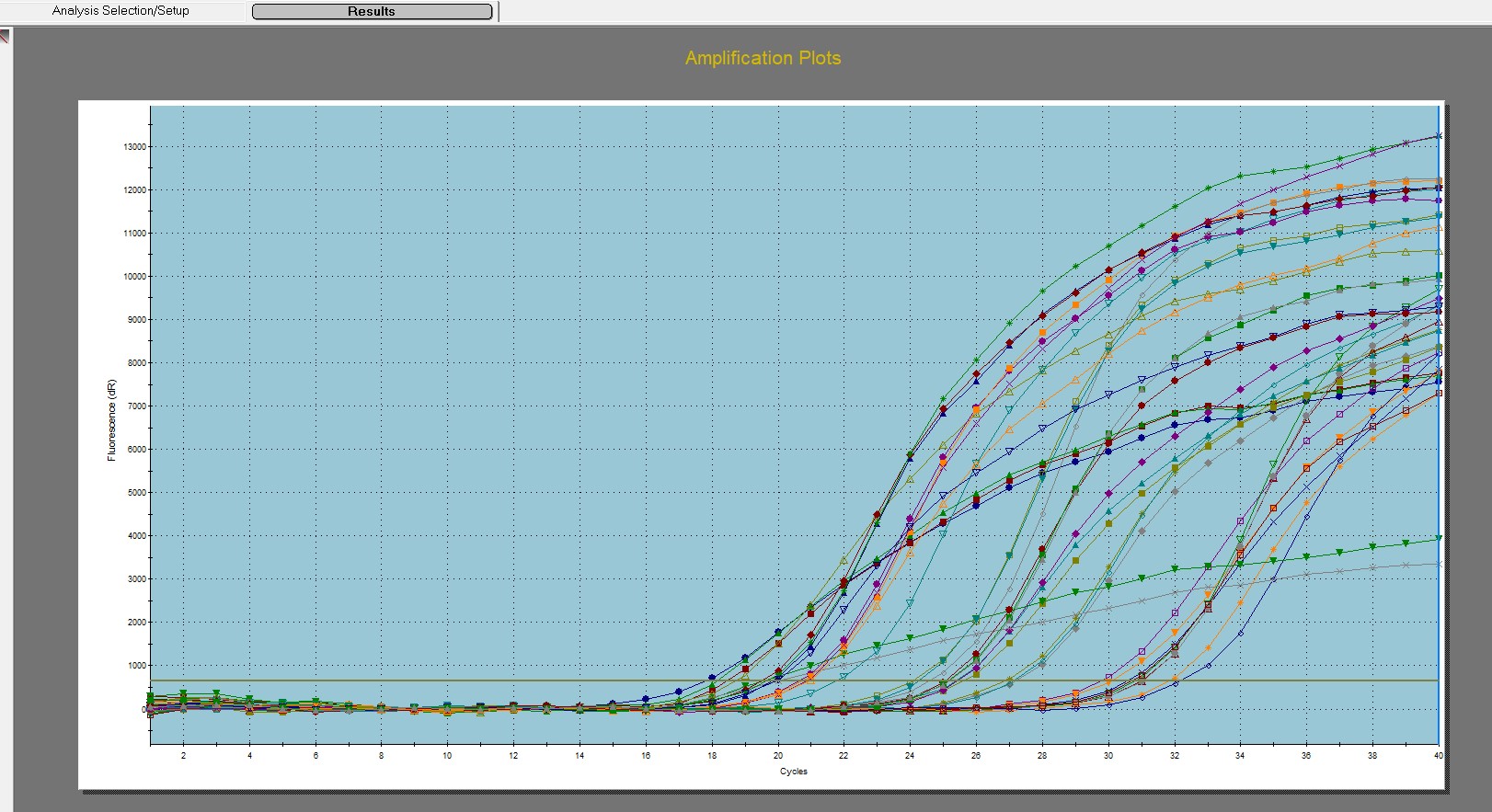

Supplement: Supplementary file 1 [file DataSheet_1.zip › excel+p/2-1.jpg]

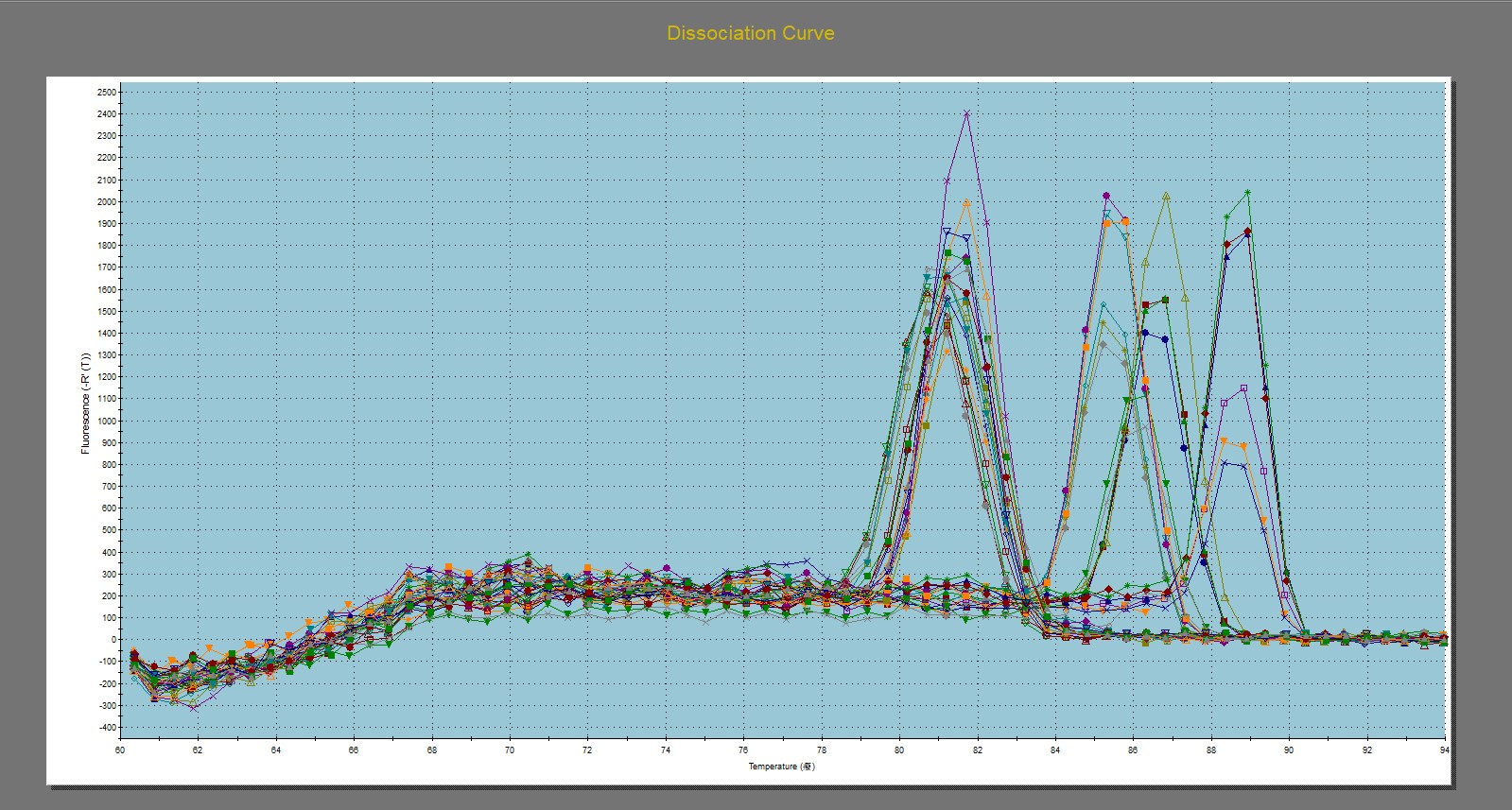

Supplement: Supplementary file 1 [file DataSheet_1.zip › excel+p/2-2.jpg]

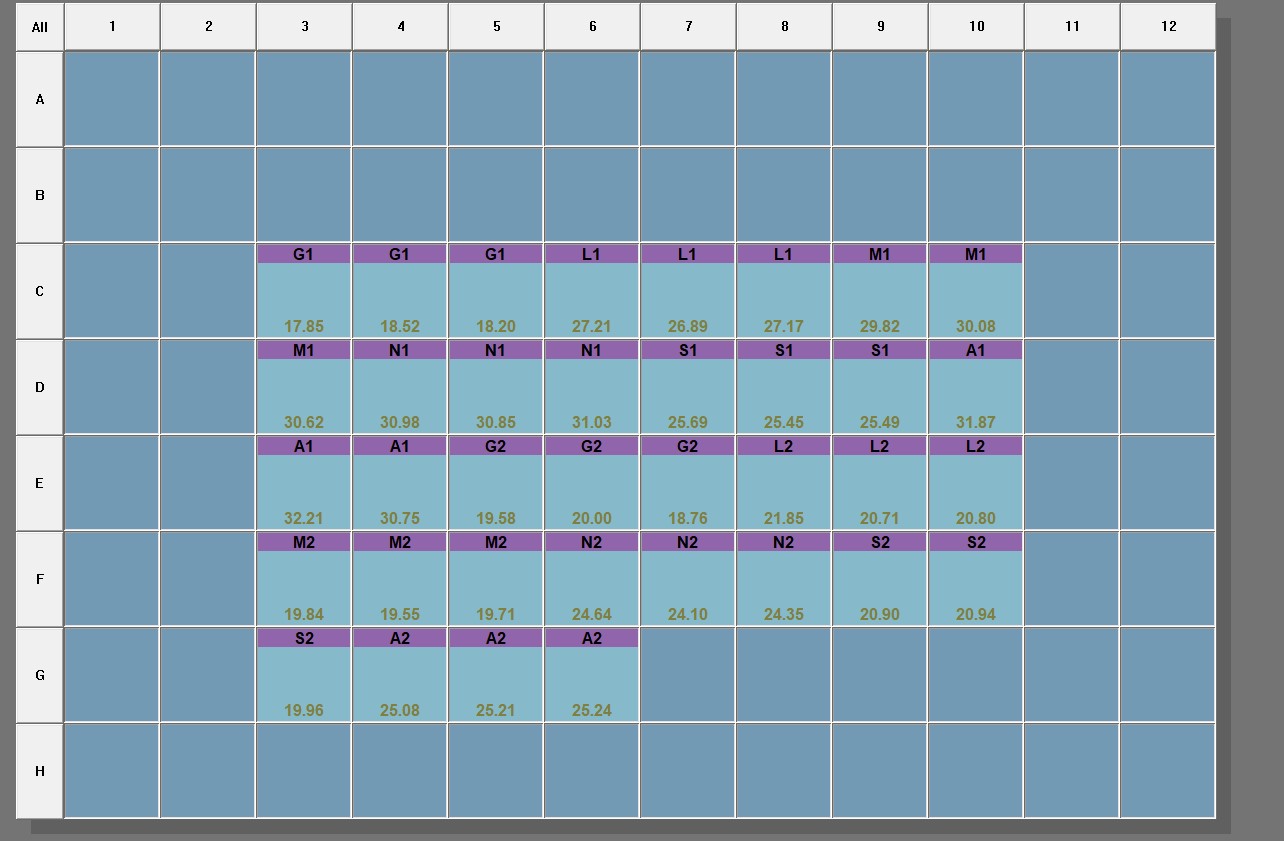

Supplement: Supplementary file 1 [file DataSheet_1.zip › excel+p/2-3.jpg]

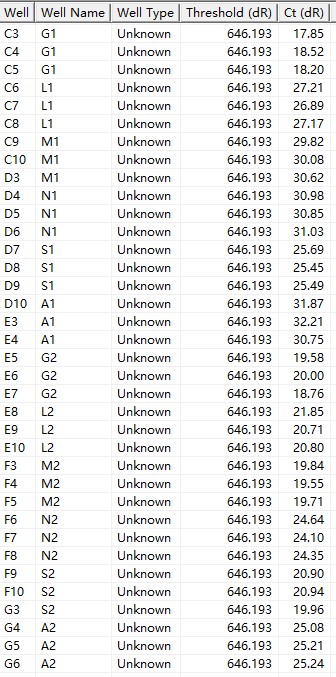

Supplement: Supplementary file 1 [file DataSheet_1.zip › excel+p/2-4.jpg]

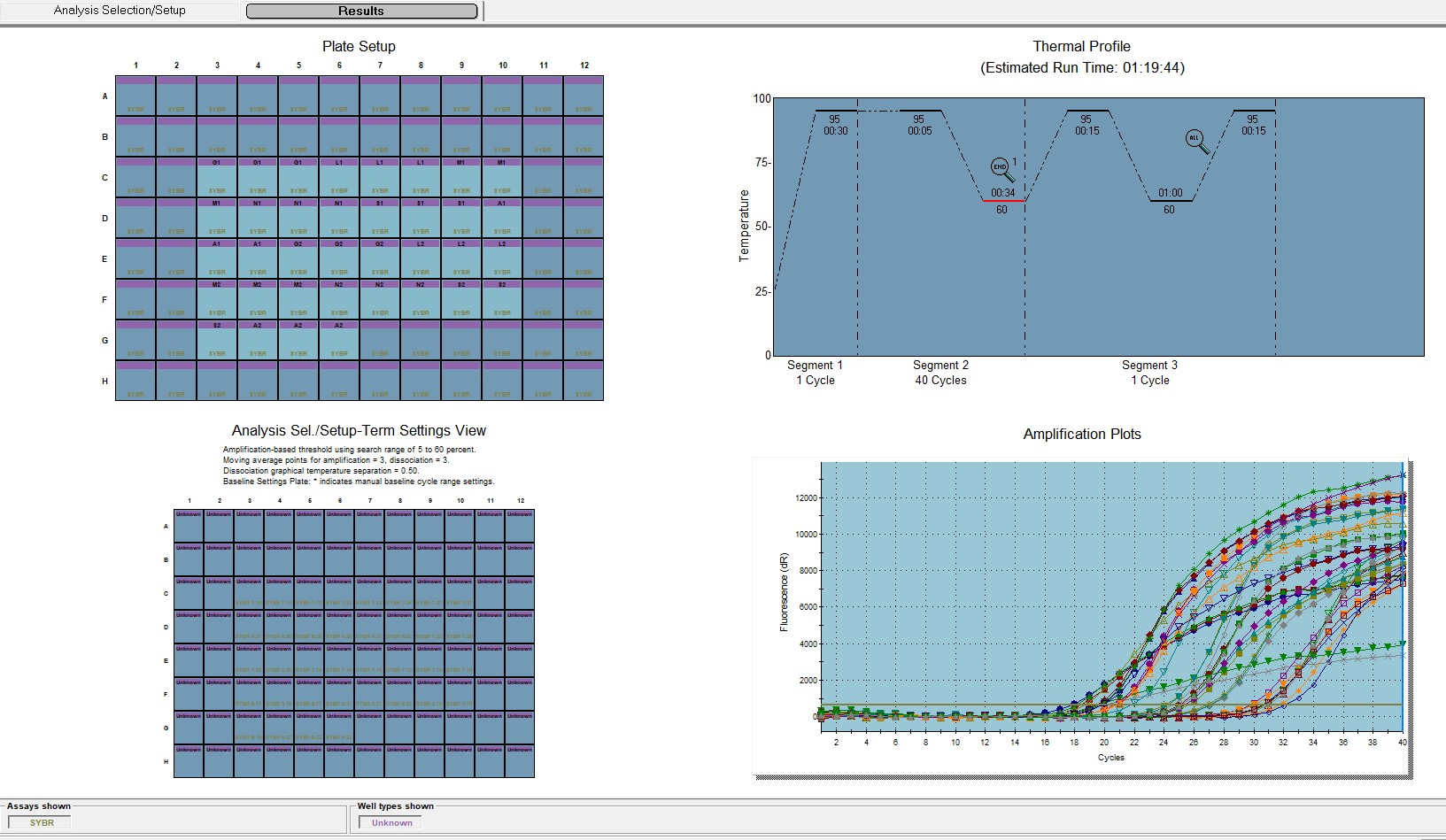

Supplement: Supplementary file 1 [file DataSheet_1.zip › excel+p/2.jpg]

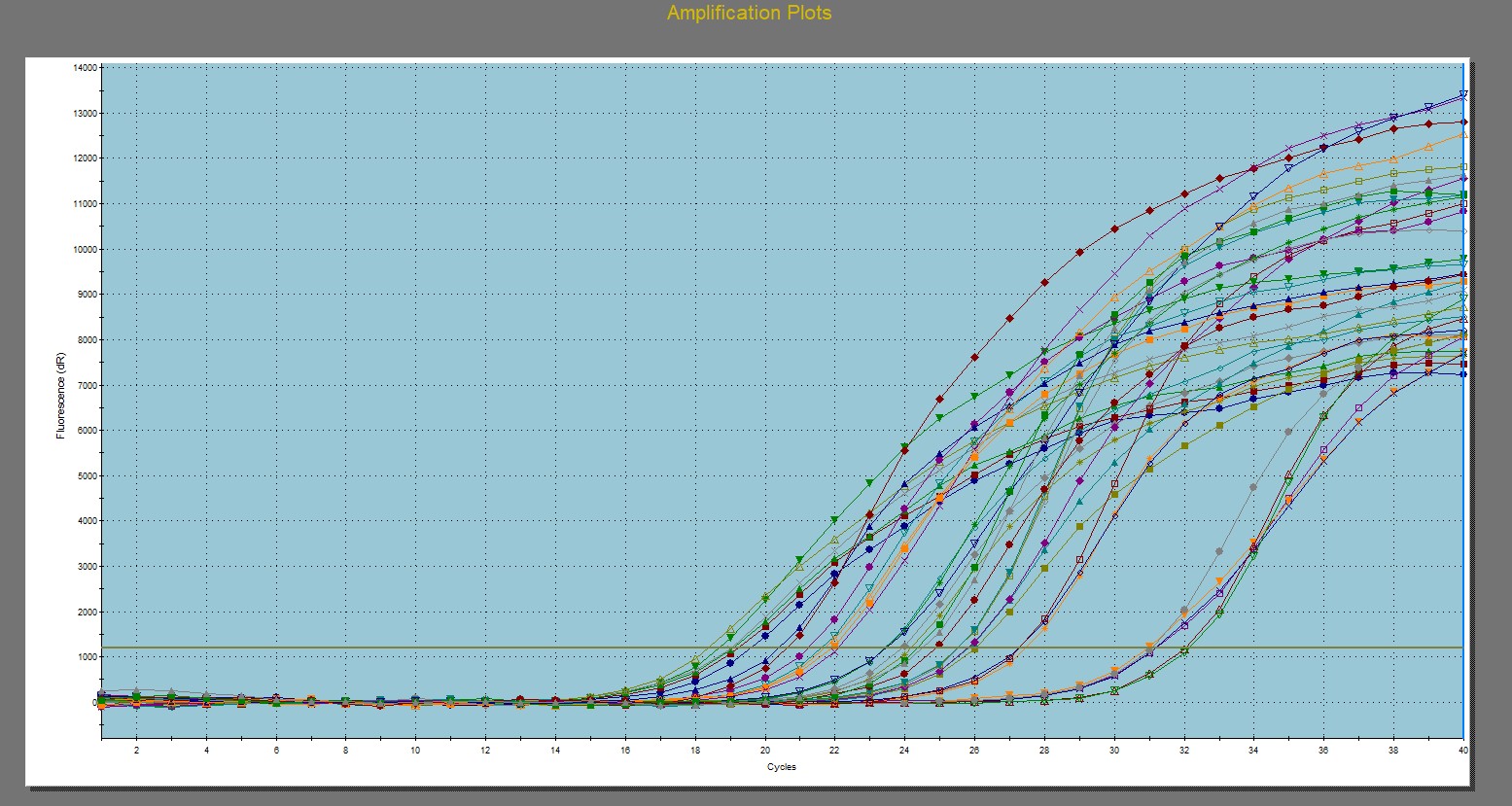

Supplement: Supplementary file 1 [file DataSheet_1.zip › excel+p/20-1.jpg]

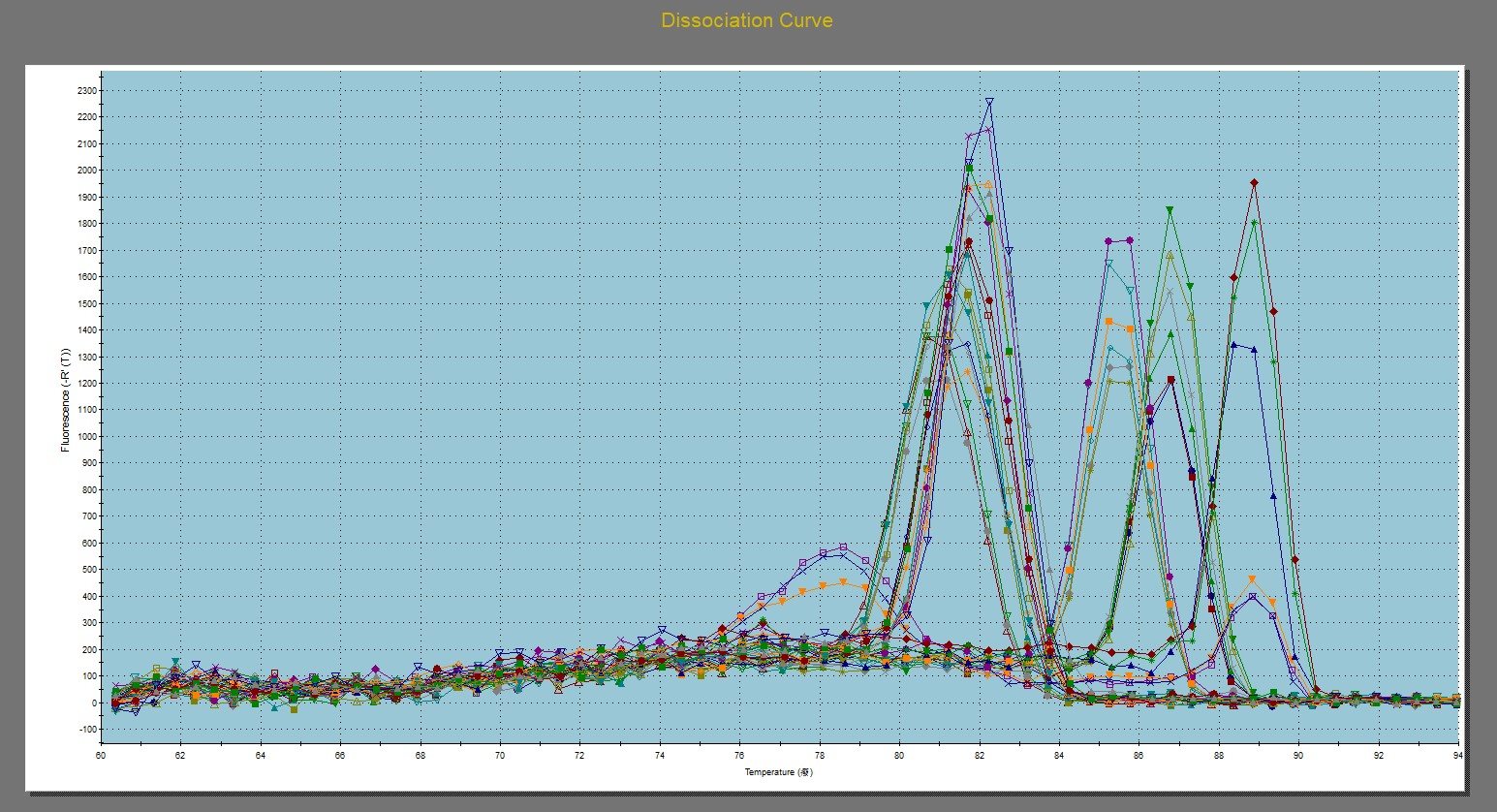

Supplement: Supplementary file 1 [file DataSheet_1.zip › excel+p/20-2.jpg]

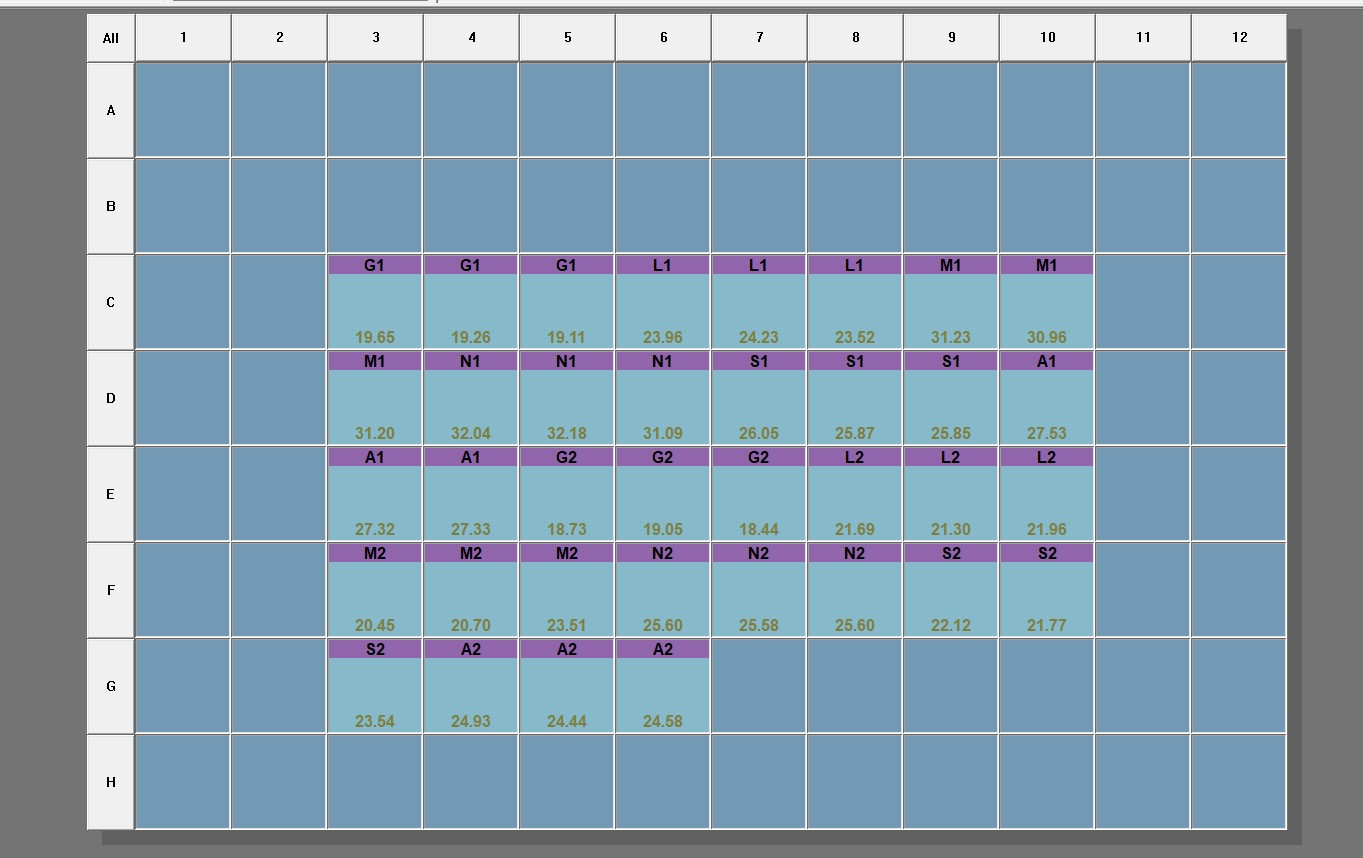

Supplement: Supplementary file 1 [file DataSheet_1.zip › excel+p/20-3.jpg]

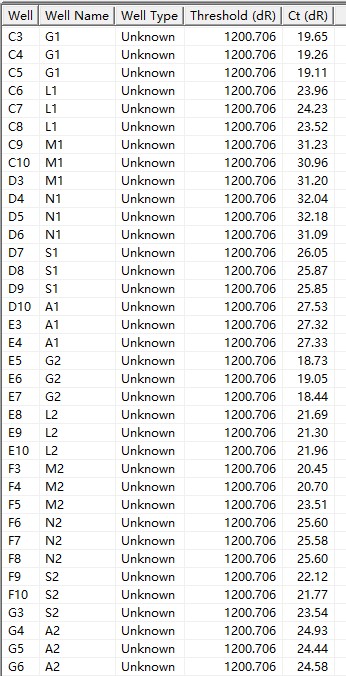

Supplement: Supplementary file 1 [file DataSheet_1.zip › excel+p/20-4.jpg]

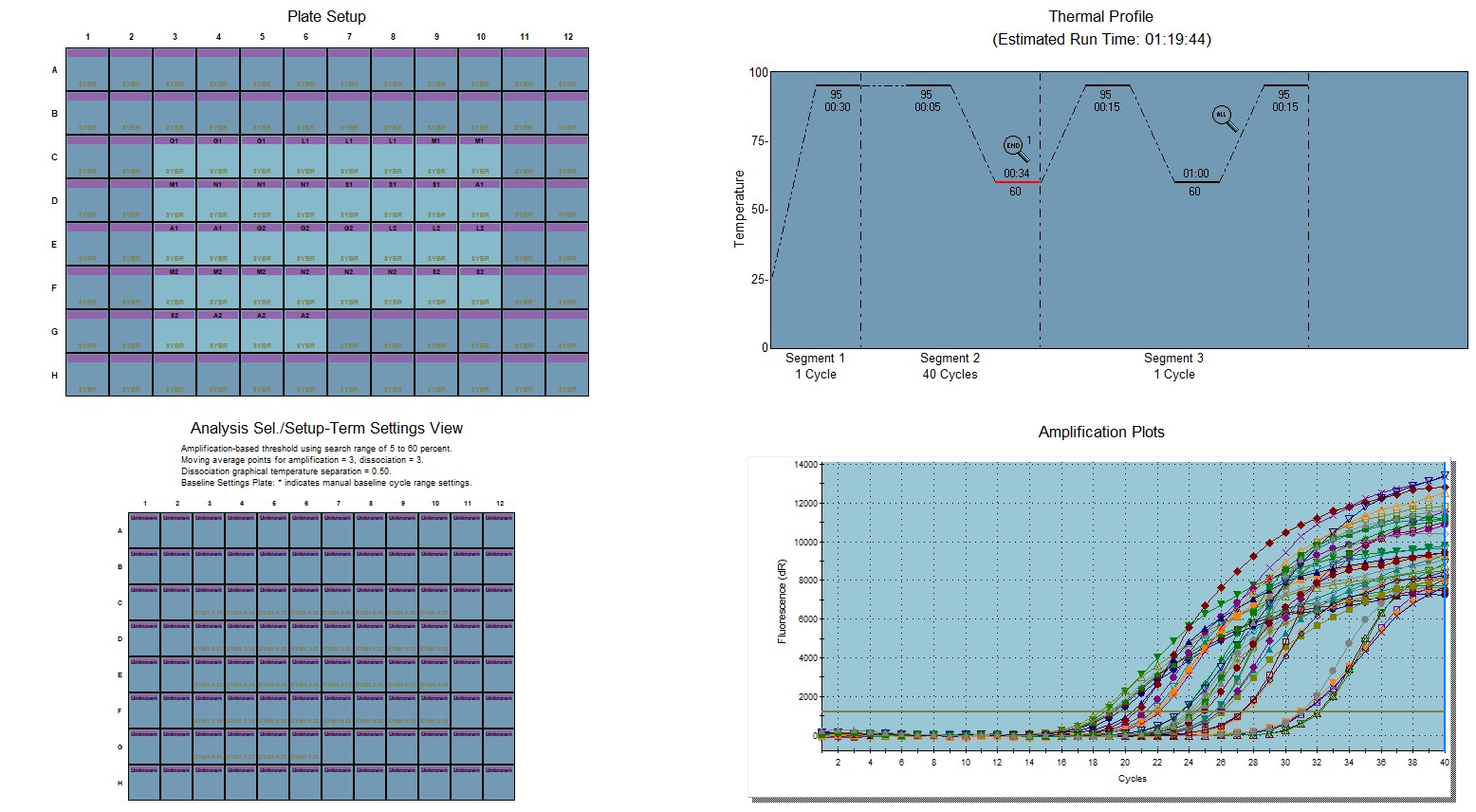

Supplement: Supplementary file 1 [file DataSheet_1.zip › excel+p/20.jpg]

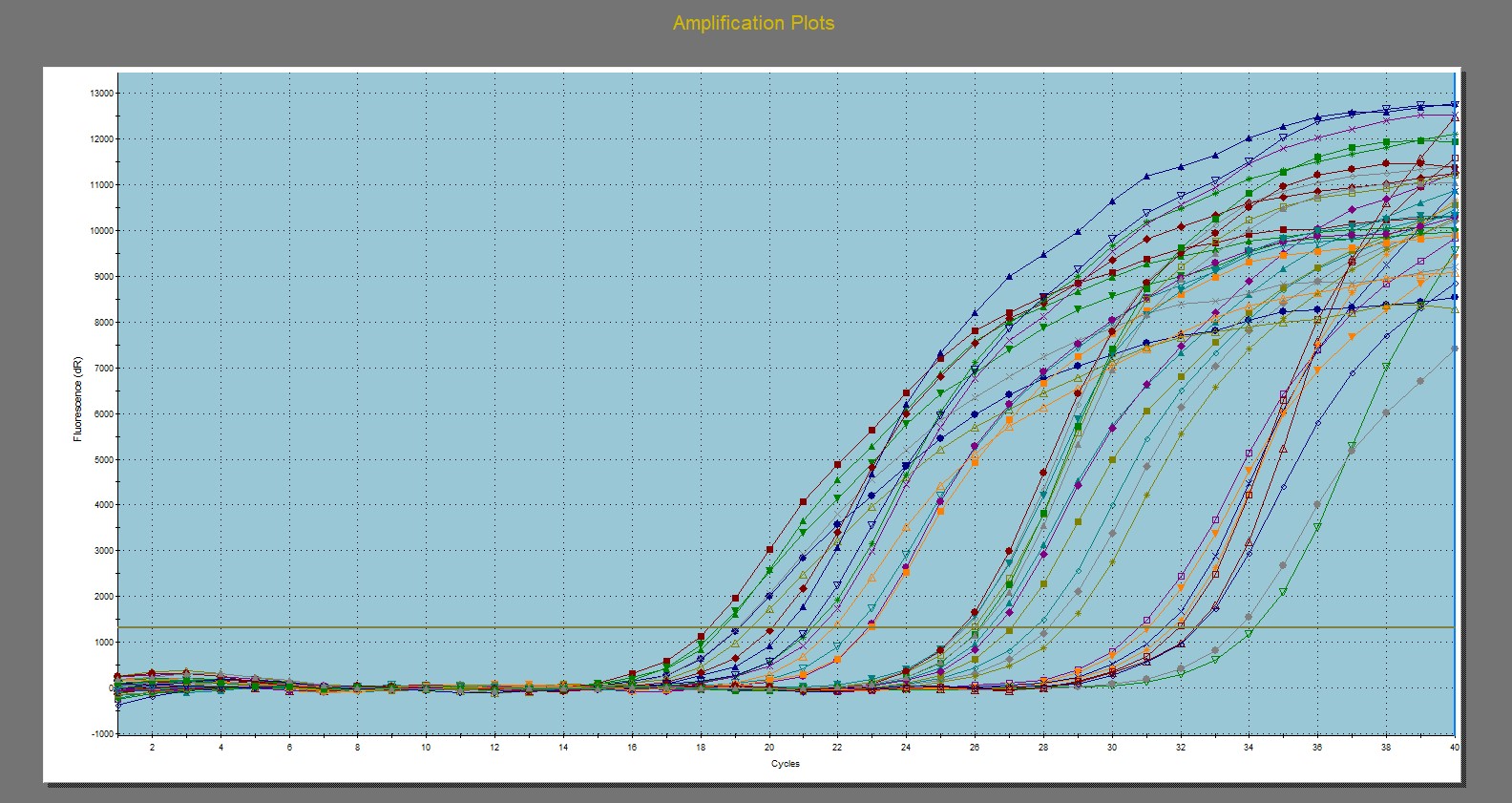

Supplement: Supplementary file 1 [file DataSheet_1.zip › excel+p/3-1.jpg]

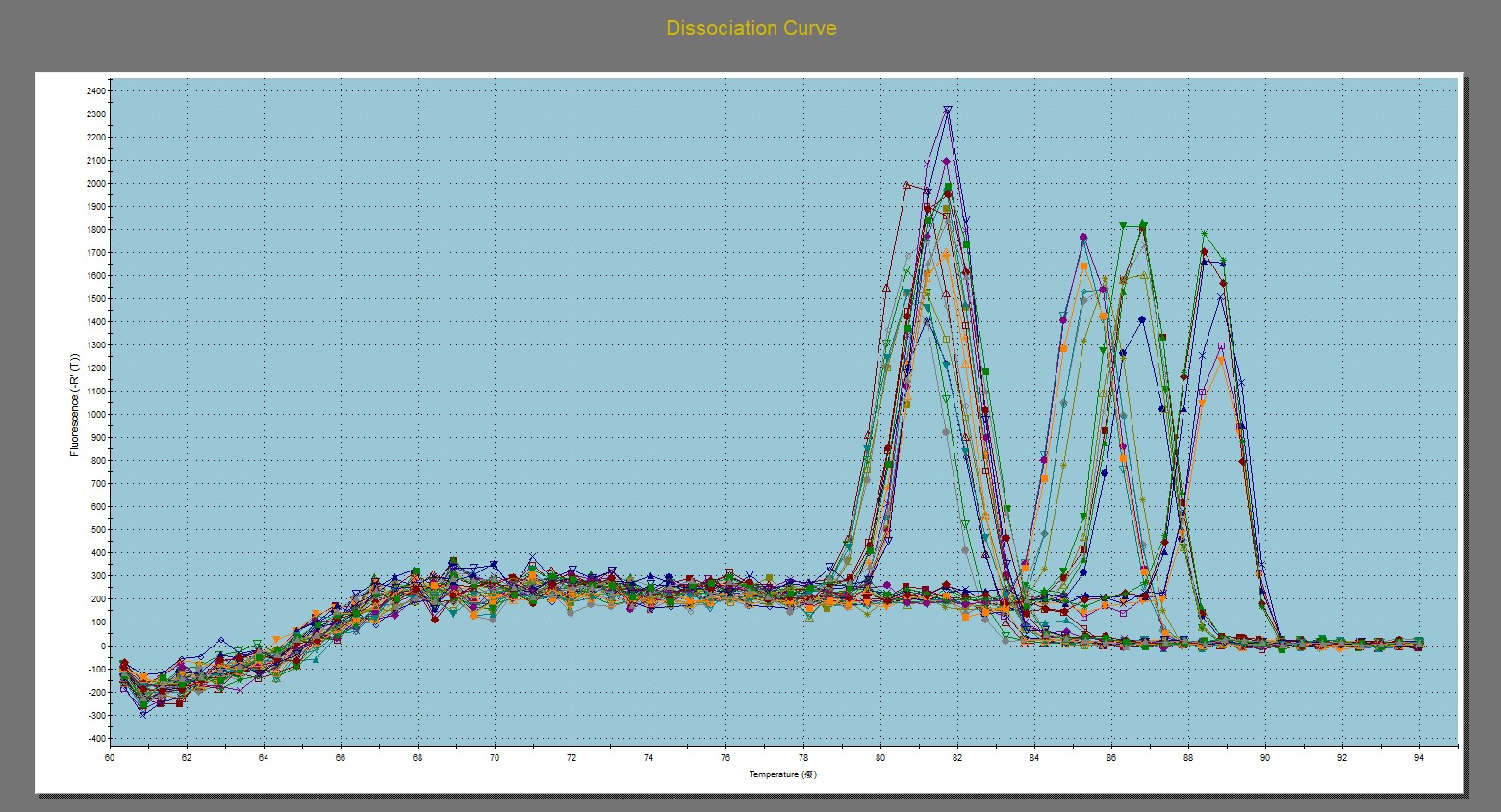

Supplement: Supplementary file 1 [file DataSheet_1.zip › excel+p/3-2.jpg]

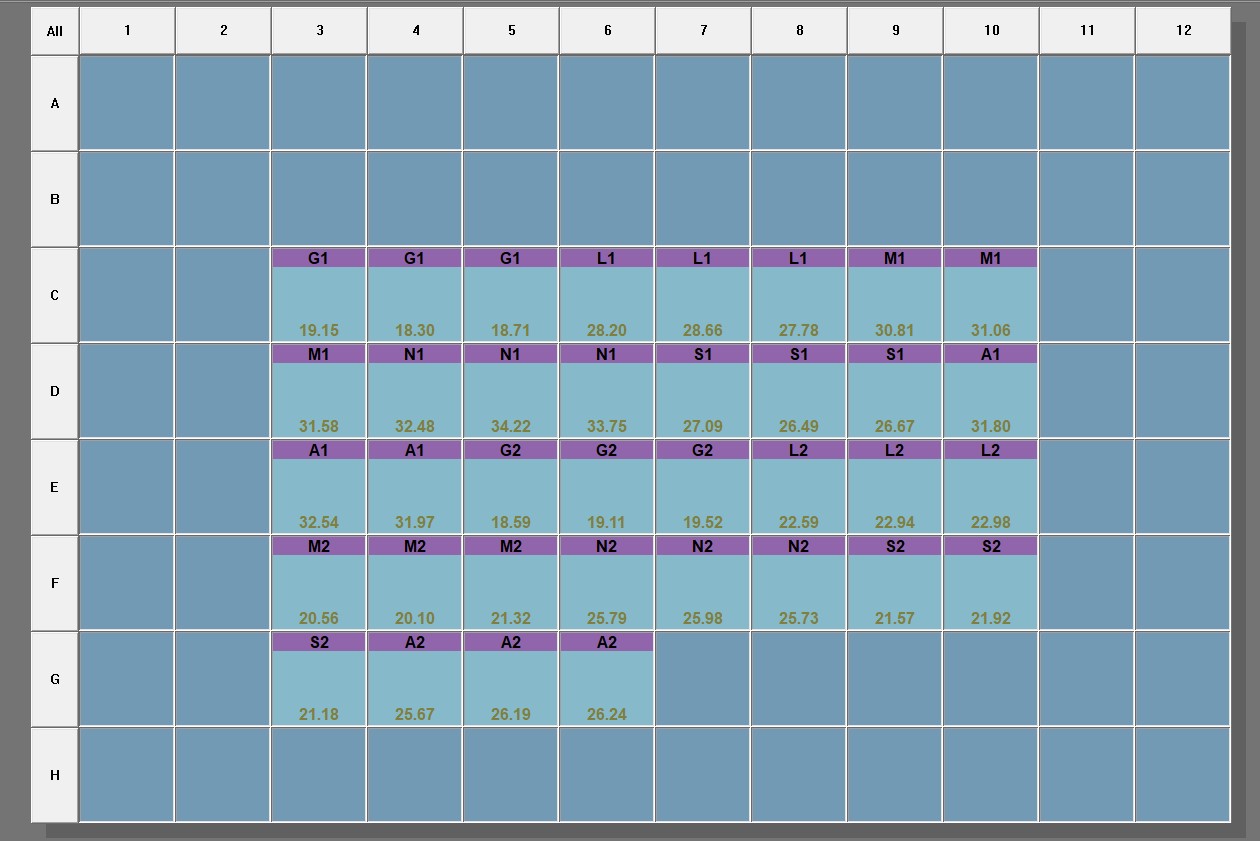

Supplement: Supplementary file 1 [file DataSheet_1.zip › excel+p/3-3.jpg]

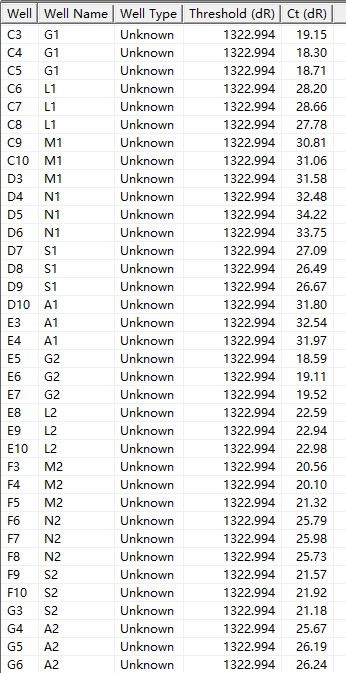

Supplement: Supplementary file 1 [file DataSheet_1.zip › excel+p/3-4.jpg]

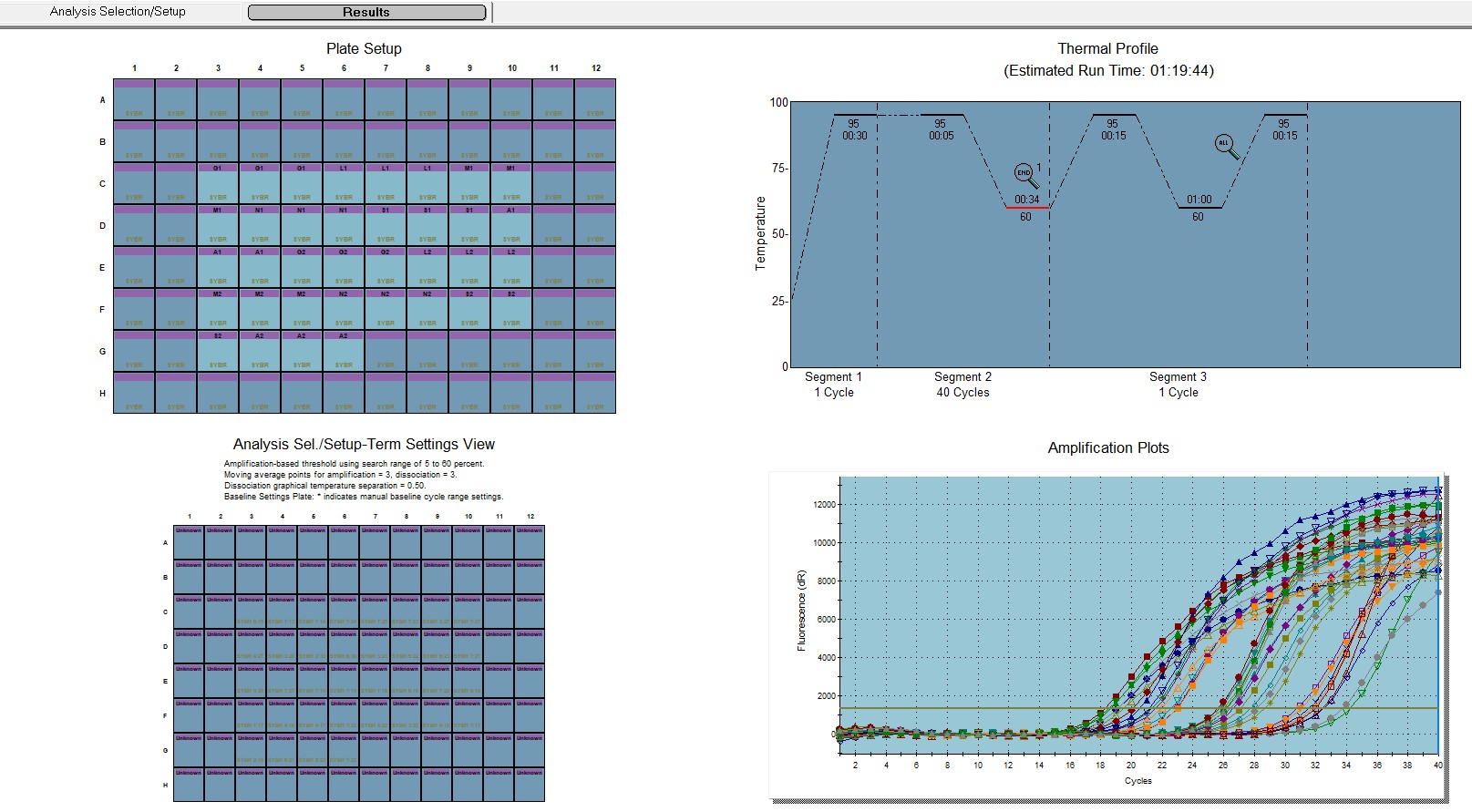

Supplement: Supplementary file 1 [file DataSheet_1.zip › excel+p/3.jpg]

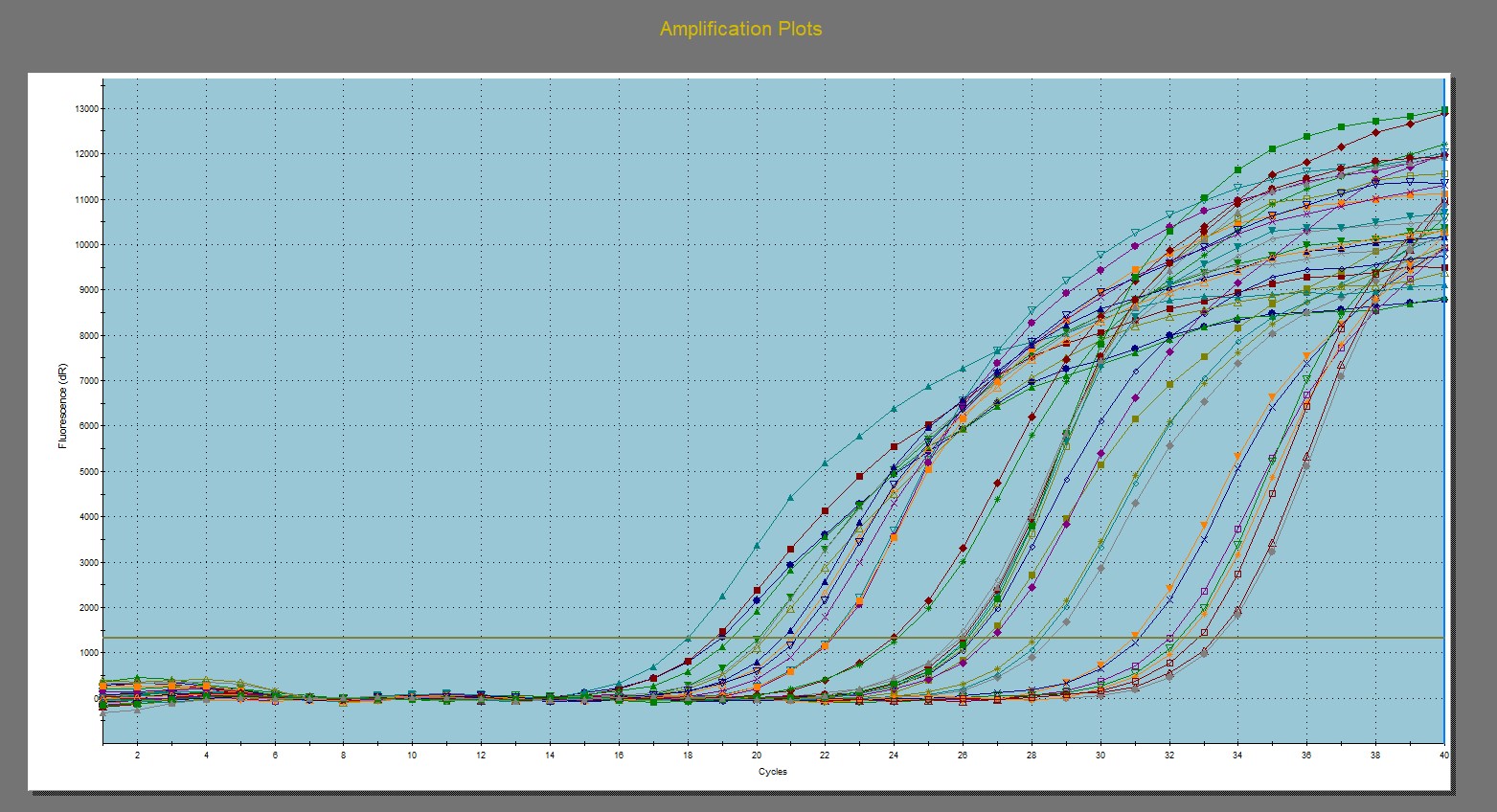

Supplement: Supplementary file 1 [file DataSheet_1.zip › excel+p/4-1.jpg]

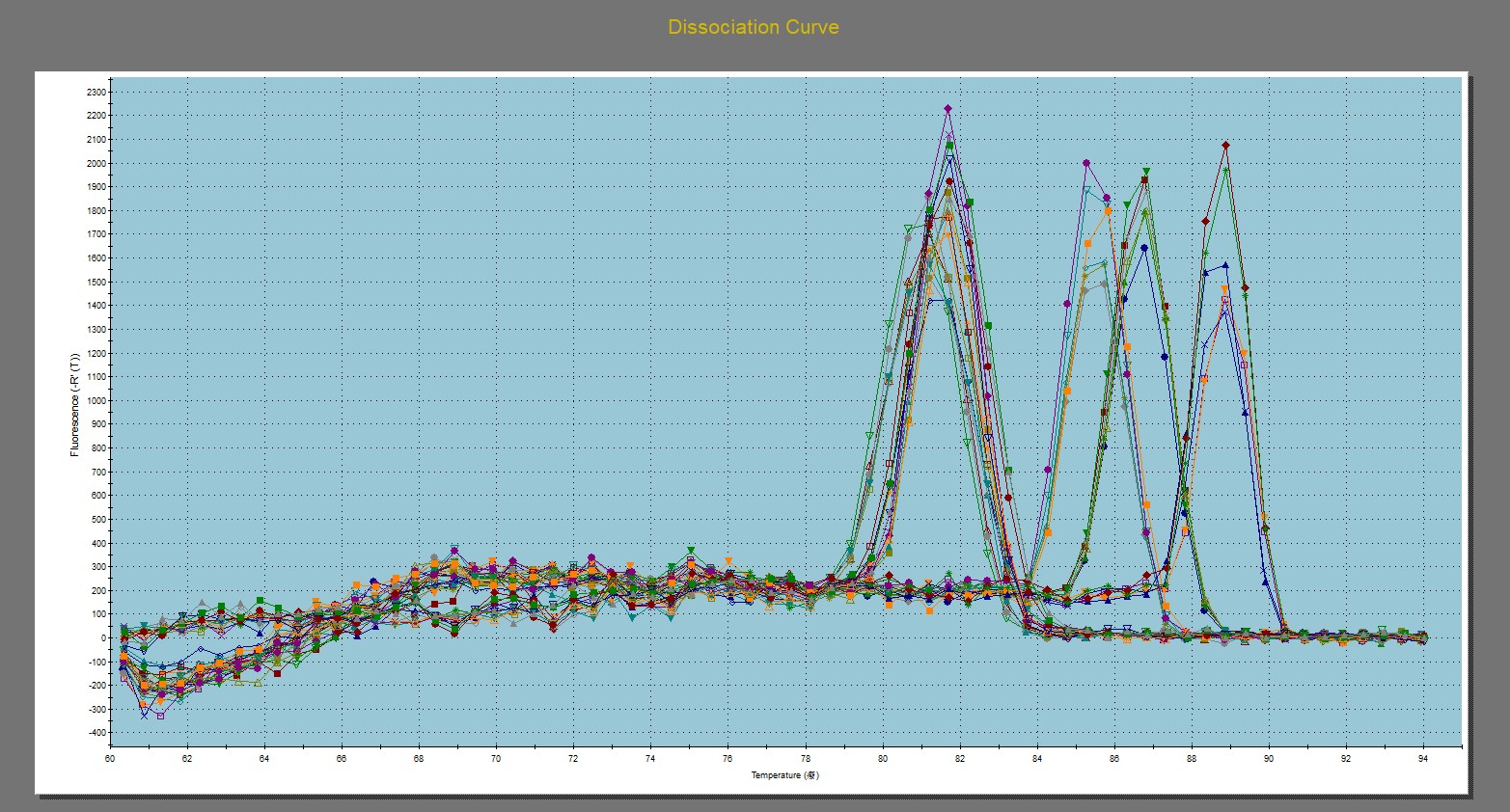

Supplement: Supplementary file 1 [file DataSheet_1.zip › excel+p/4-2.jpg]

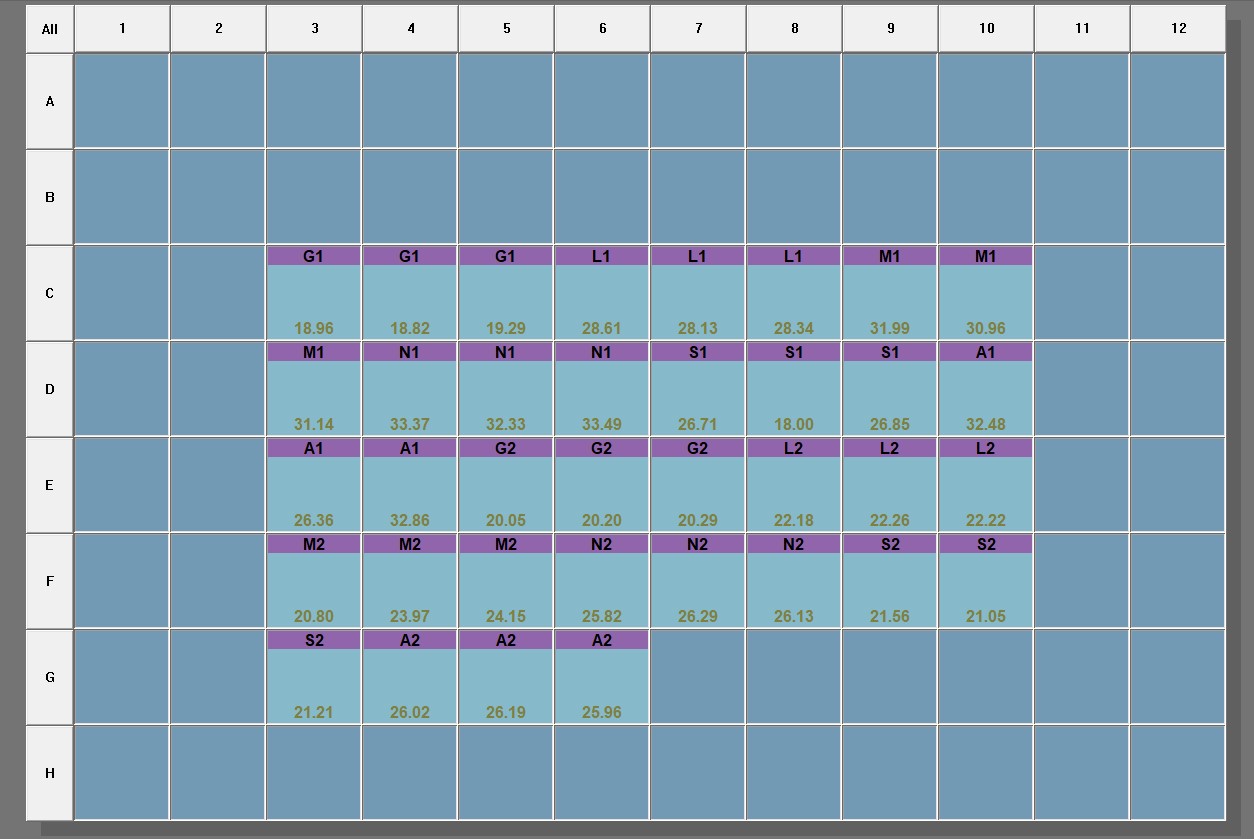

Supplement: Supplementary file 1 [file DataSheet_1.zip › excel+p/4-3.jpg]

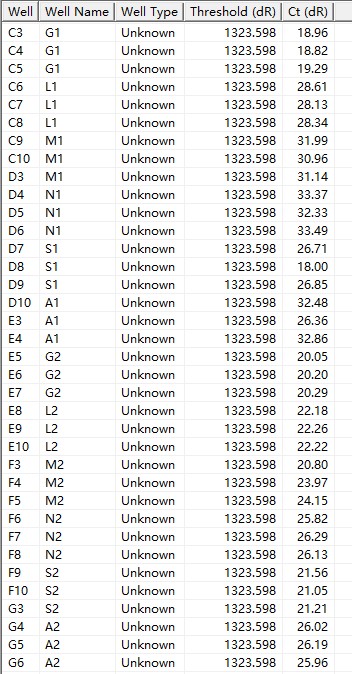

Supplement: Supplementary file 1 [file DataSheet_1.zip › excel+p/4-4.jpg]

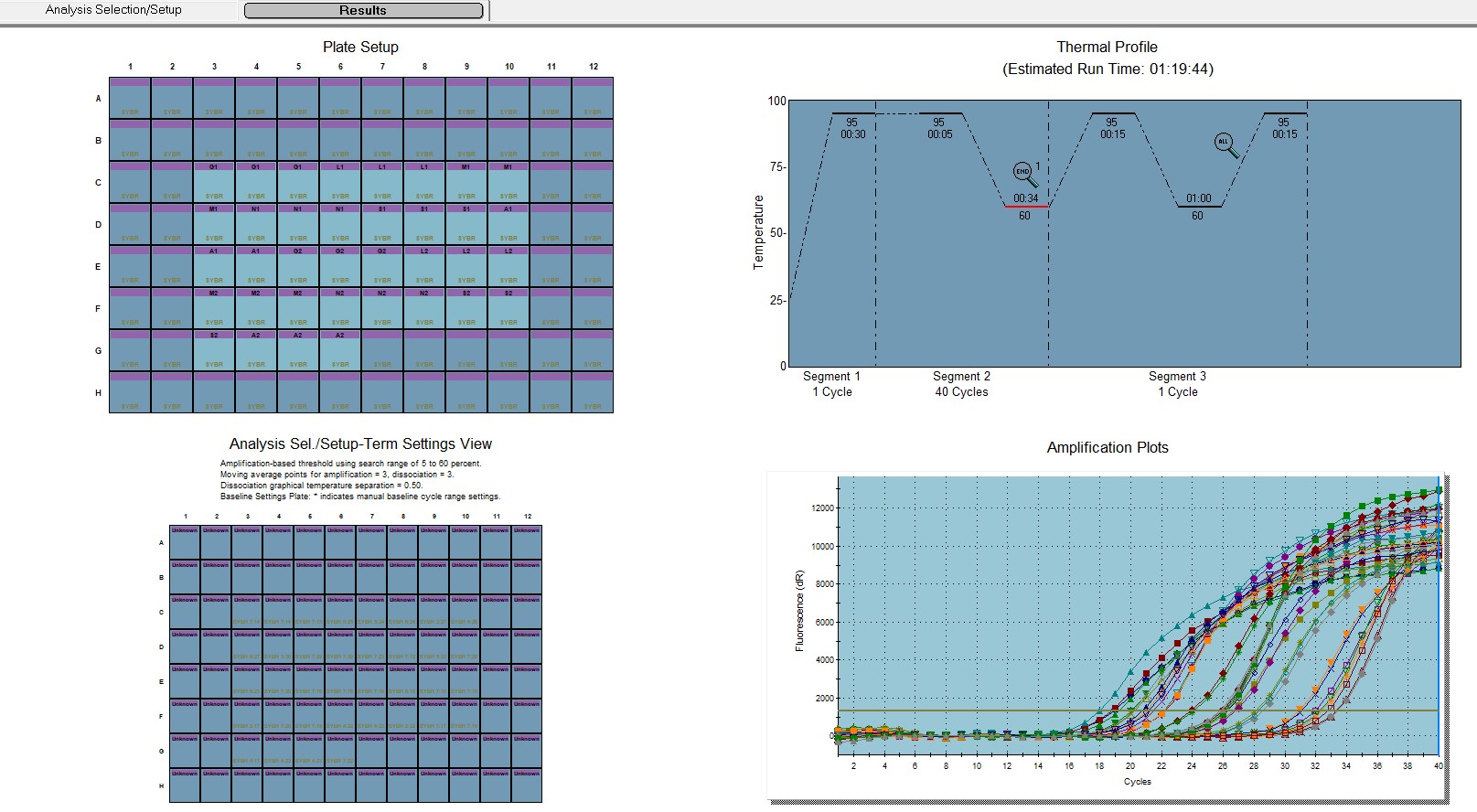

Supplement: Supplementary file 1 [file DataSheet_1.zip › excel+p/4.jpg]

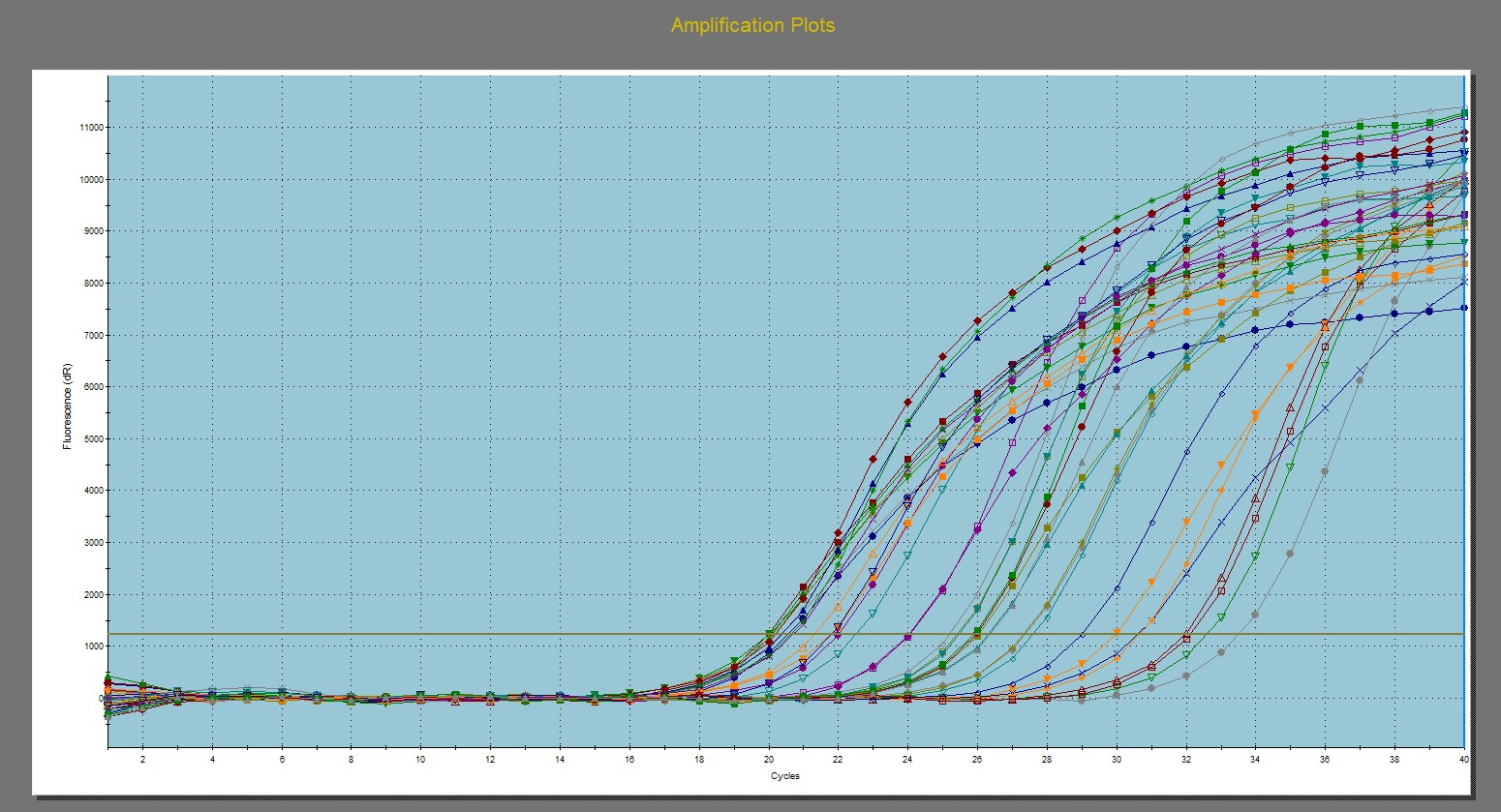

Supplement: Supplementary file 1 [file DataSheet_1.zip › excel+p/5-1.jpg]

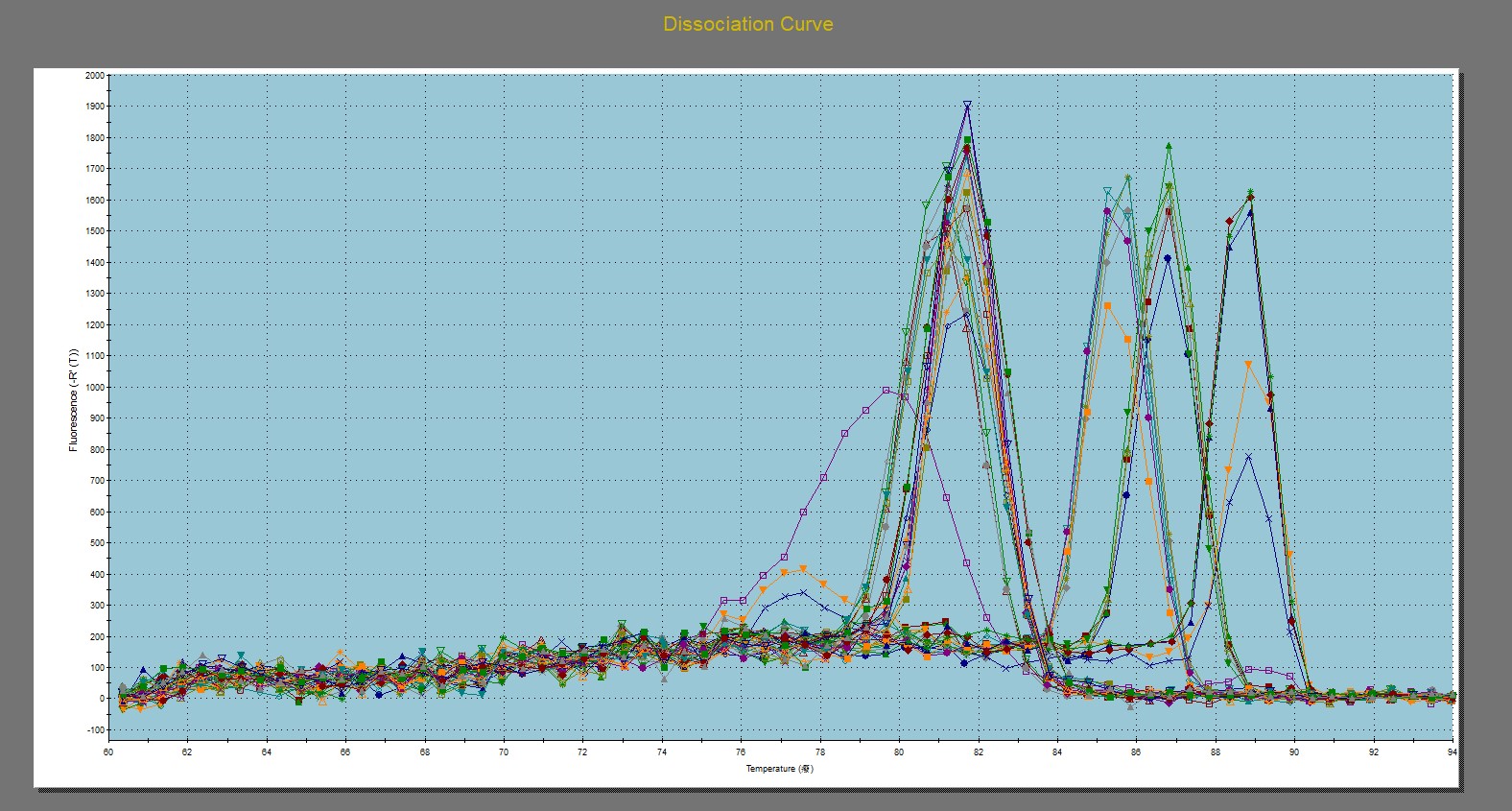

Supplement: Supplementary file 1 [file DataSheet_1.zip › excel+p/5-2.jpg]

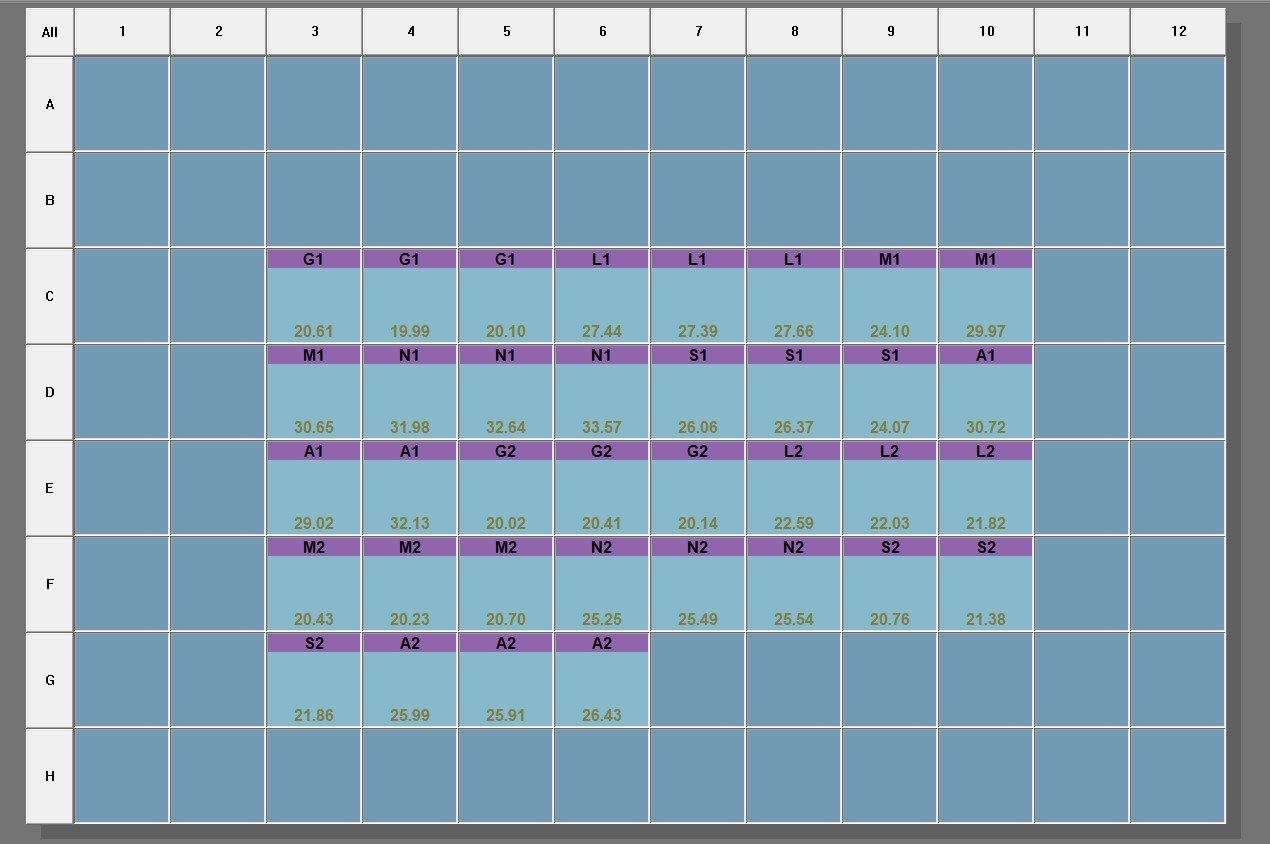

Supplement: Supplementary file 1 [file DataSheet_1.zip › excel+p/5-3.jpg]

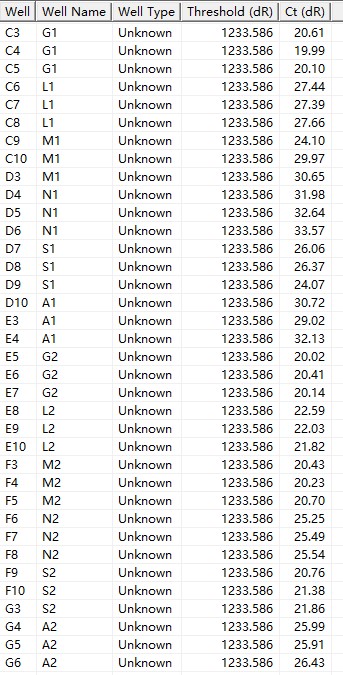

Supplement: Supplementary file 1 [file DataSheet_1.zip › excel+p/5-4.jpg]

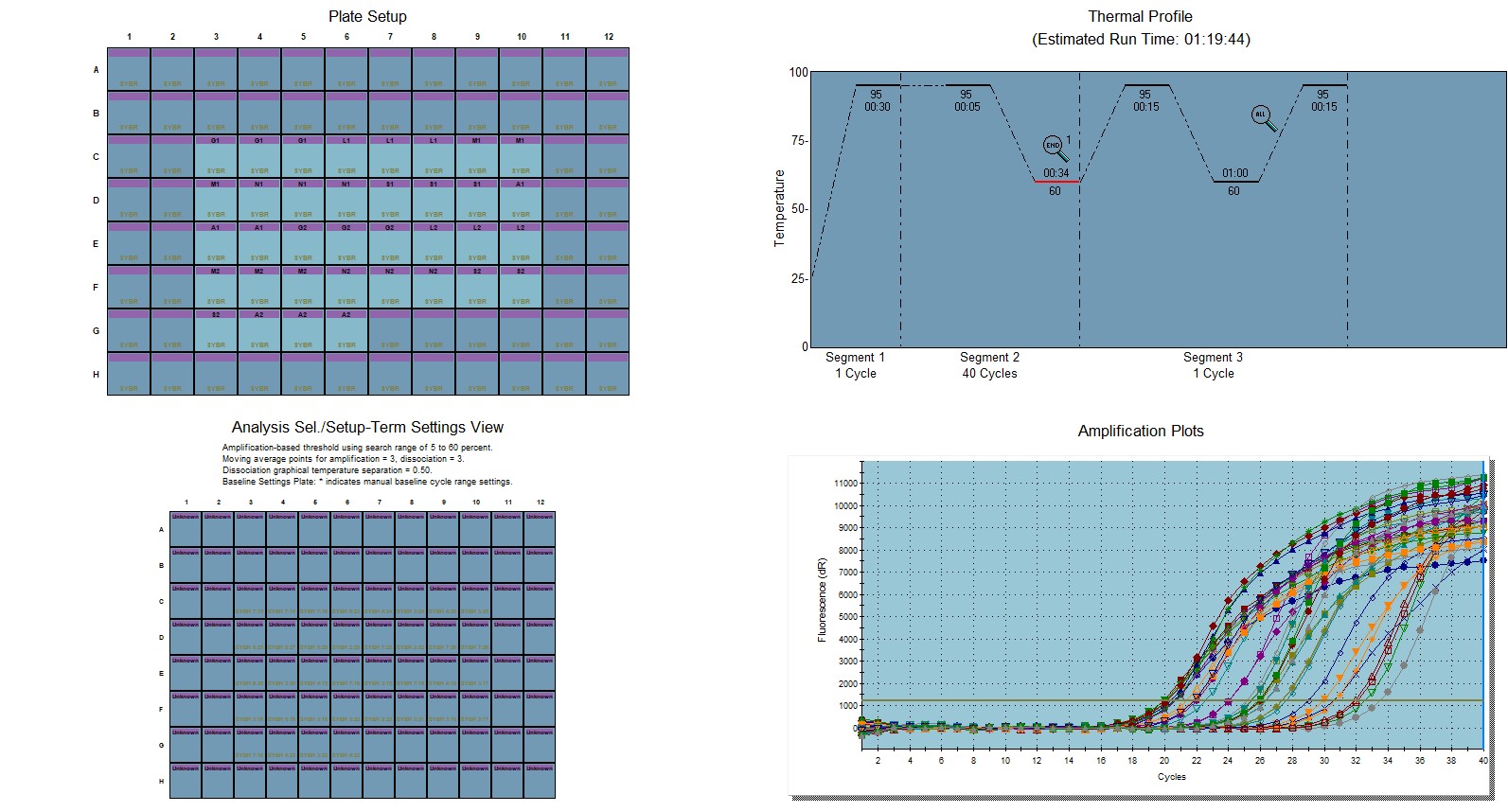

Supplement: Supplementary file 1 [file DataSheet_1.zip › excel+p/5.jpg]

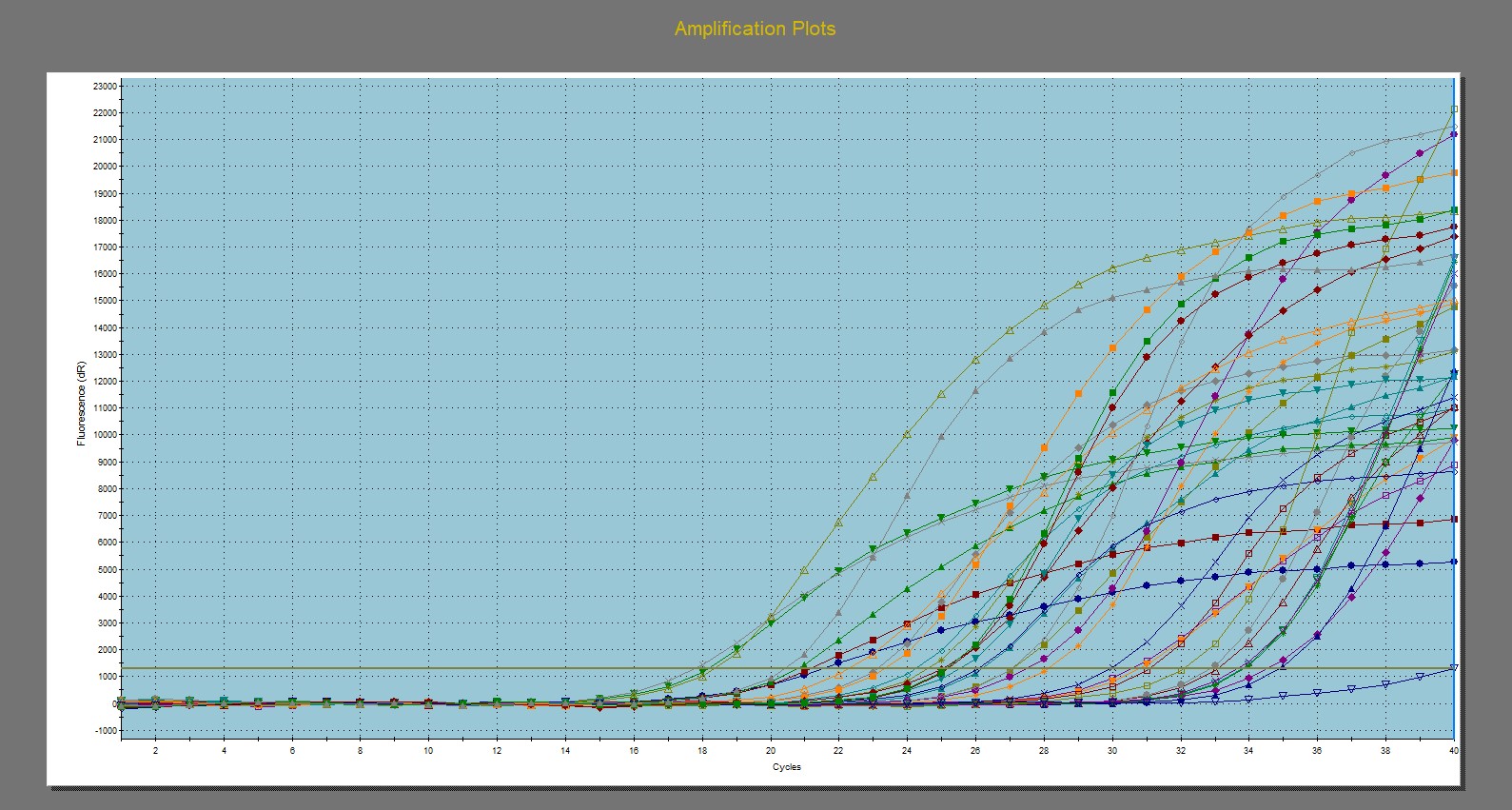

Supplement: Supplementary file 1 [file DataSheet_1.zip › excel+p/6-1.jpg]

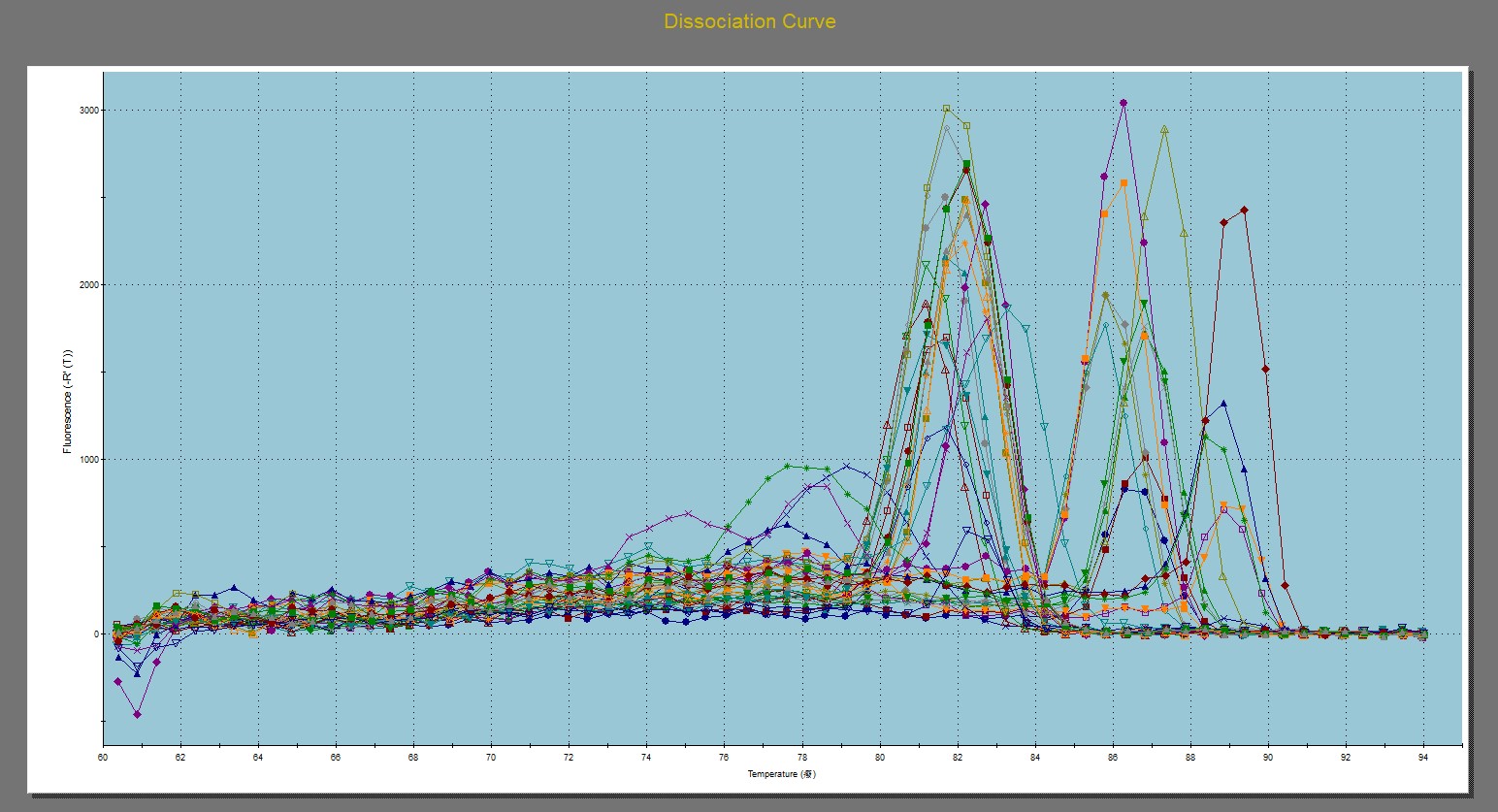

Supplement: Supplementary file 1 [file DataSheet_1.zip › excel+p/6-2.jpg]

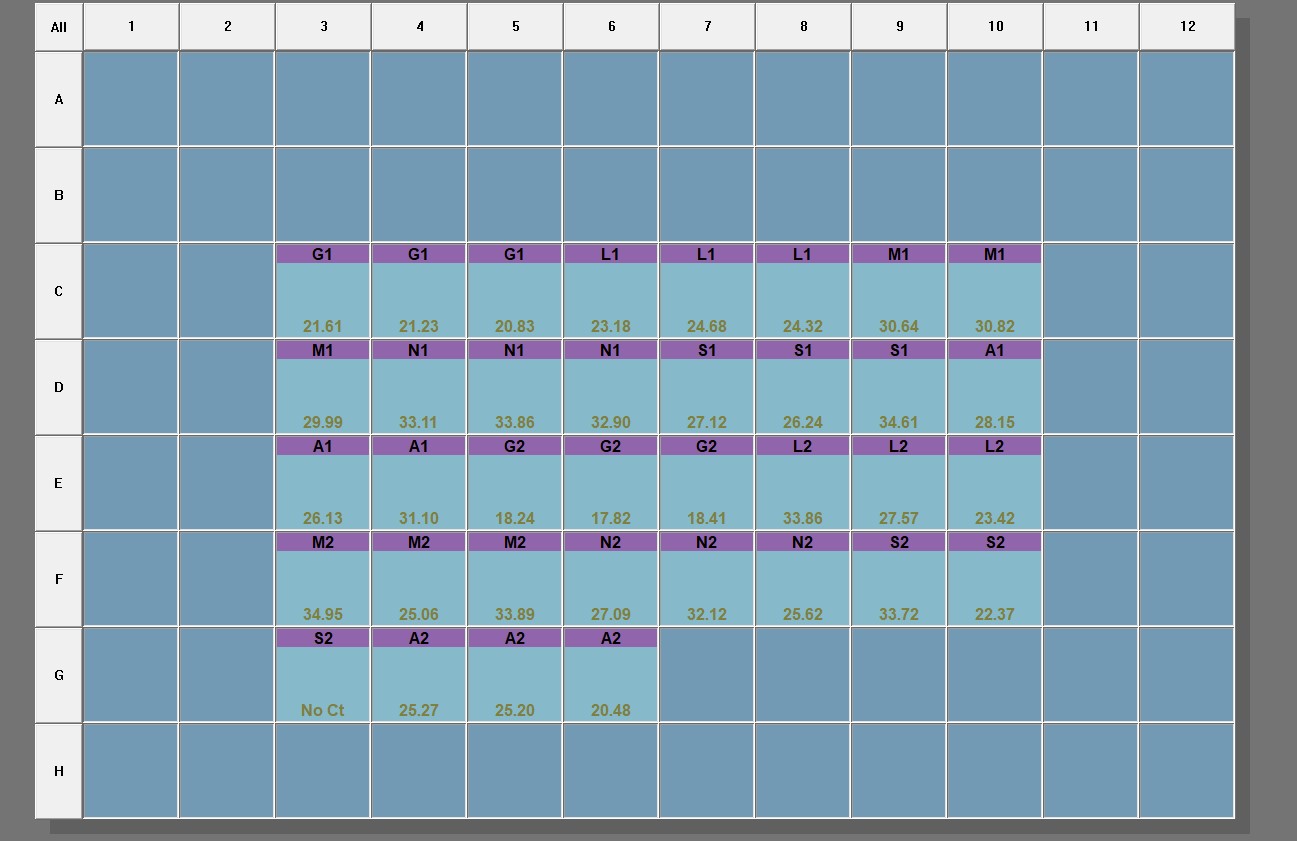

Supplement: Supplementary file 1 [file DataSheet_1.zip › excel+p/6-3.jpg]

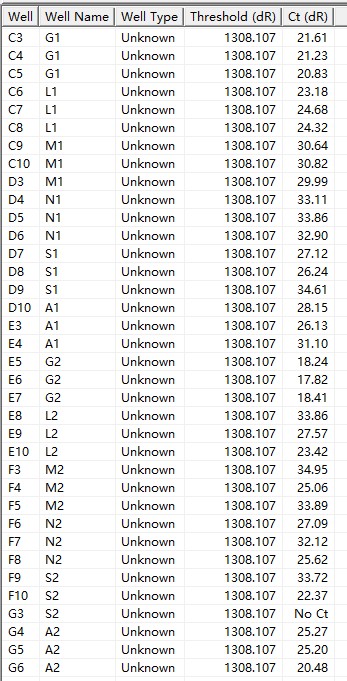

Supplement: Supplementary file 1 [file DataSheet_1.zip › excel+p/6-4.jpg]

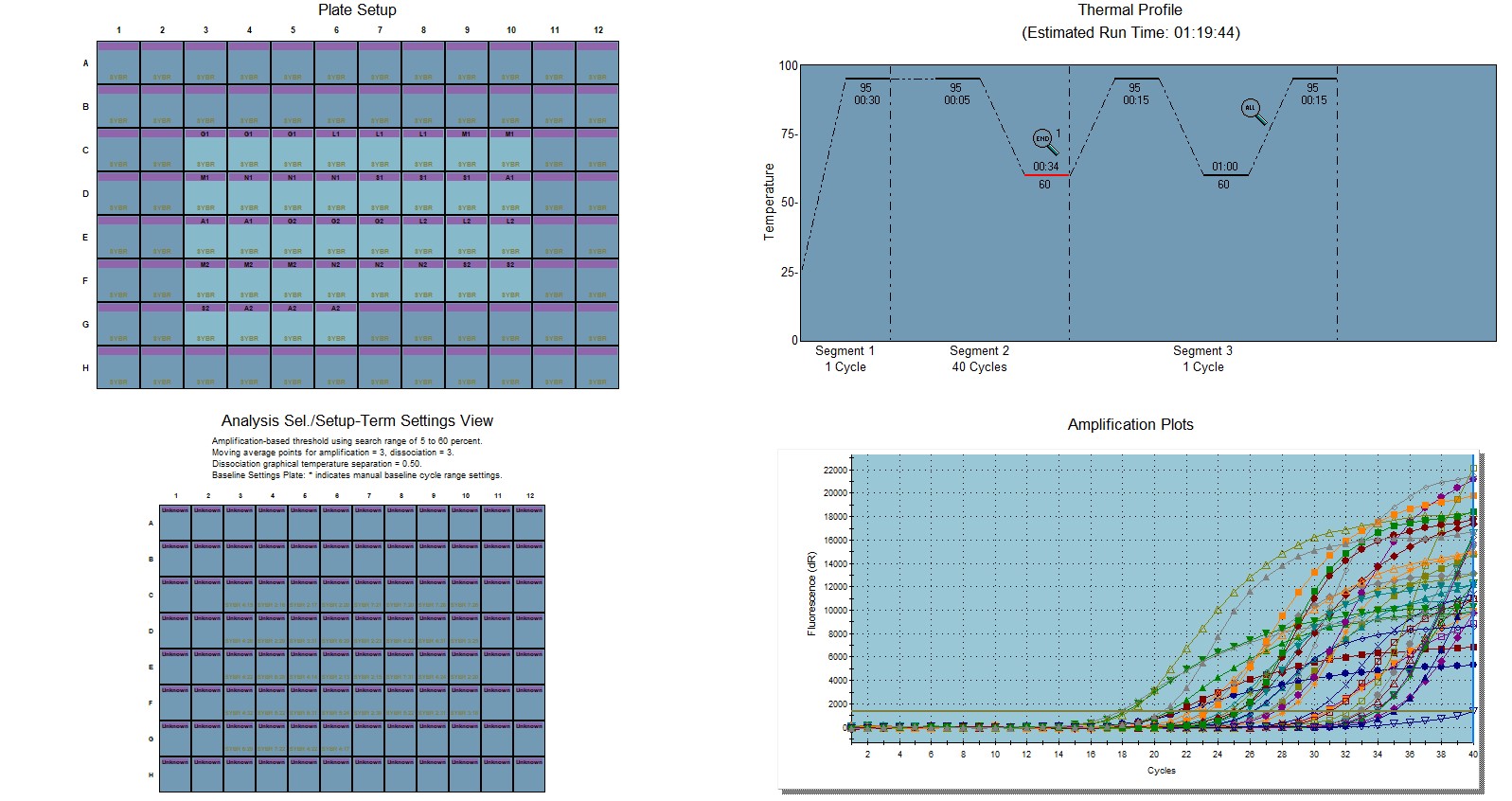

Supplement: Supplementary file 1 [file DataSheet_1.zip › excel+p/6.jpg]

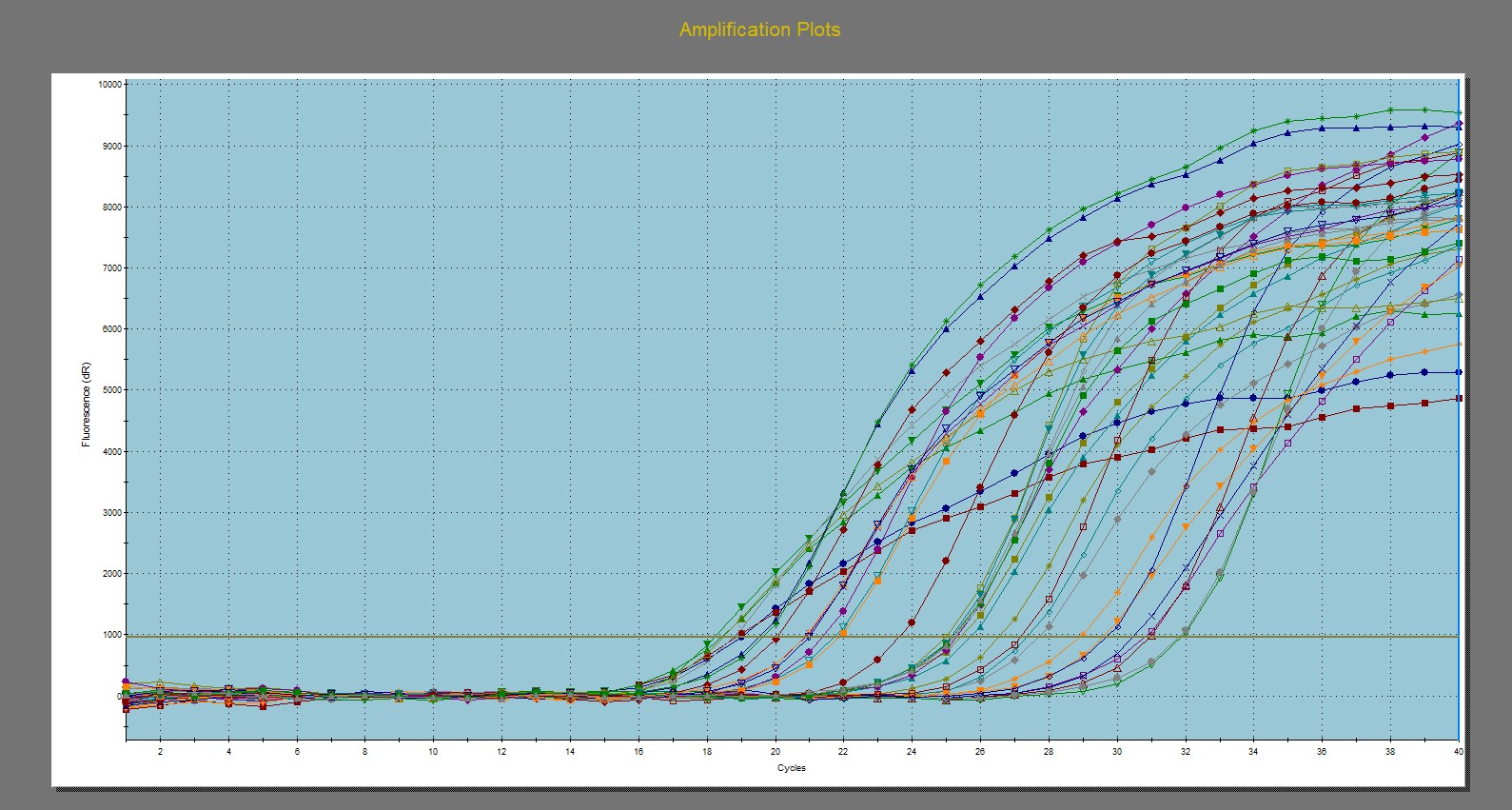

Supplement: Supplementary file 1 [file DataSheet_1.zip › excel+p/7-1.jpg]

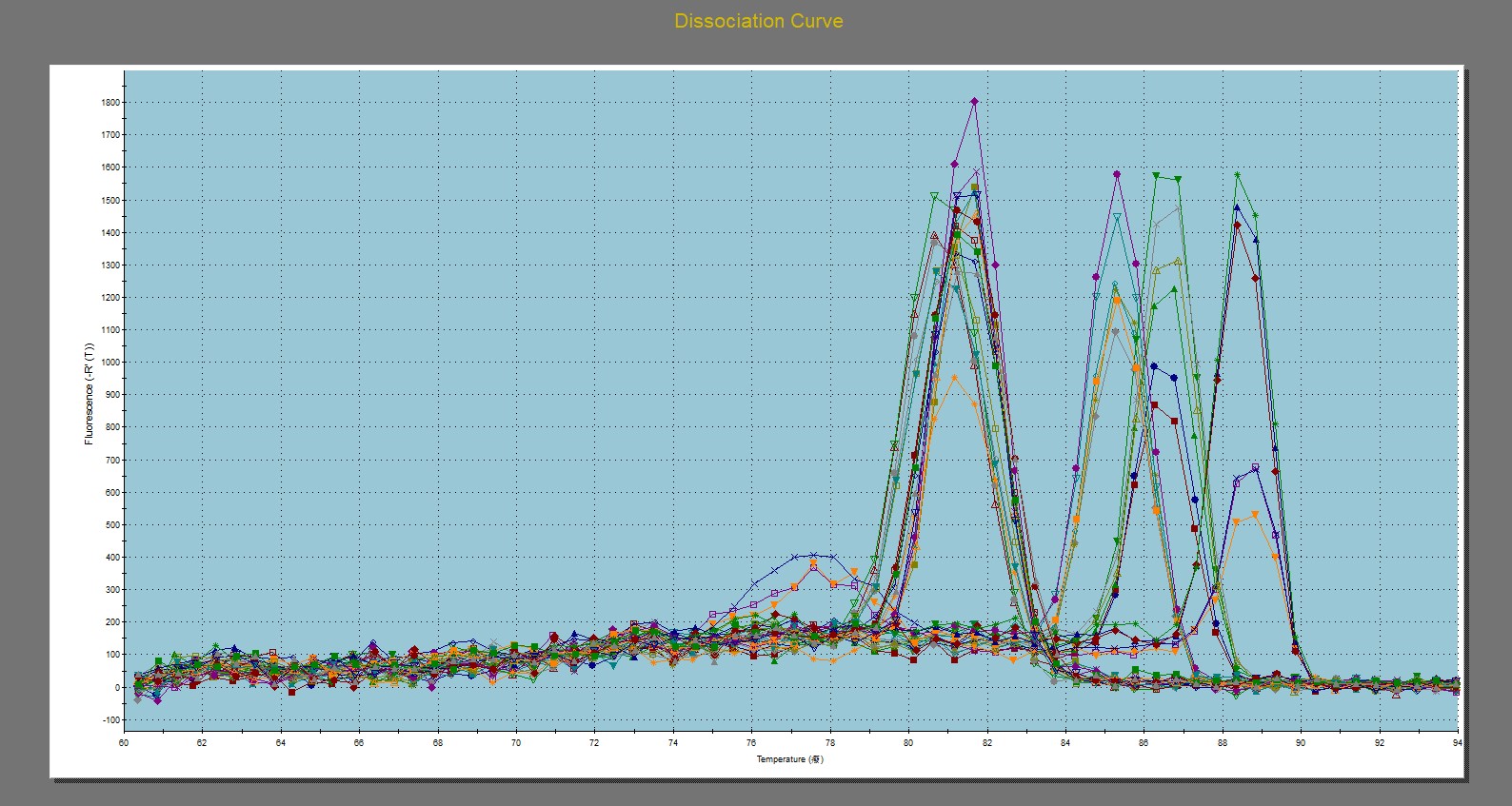

Supplement: Supplementary file 1 [file DataSheet_1.zip › excel+p/7-2.jpg]

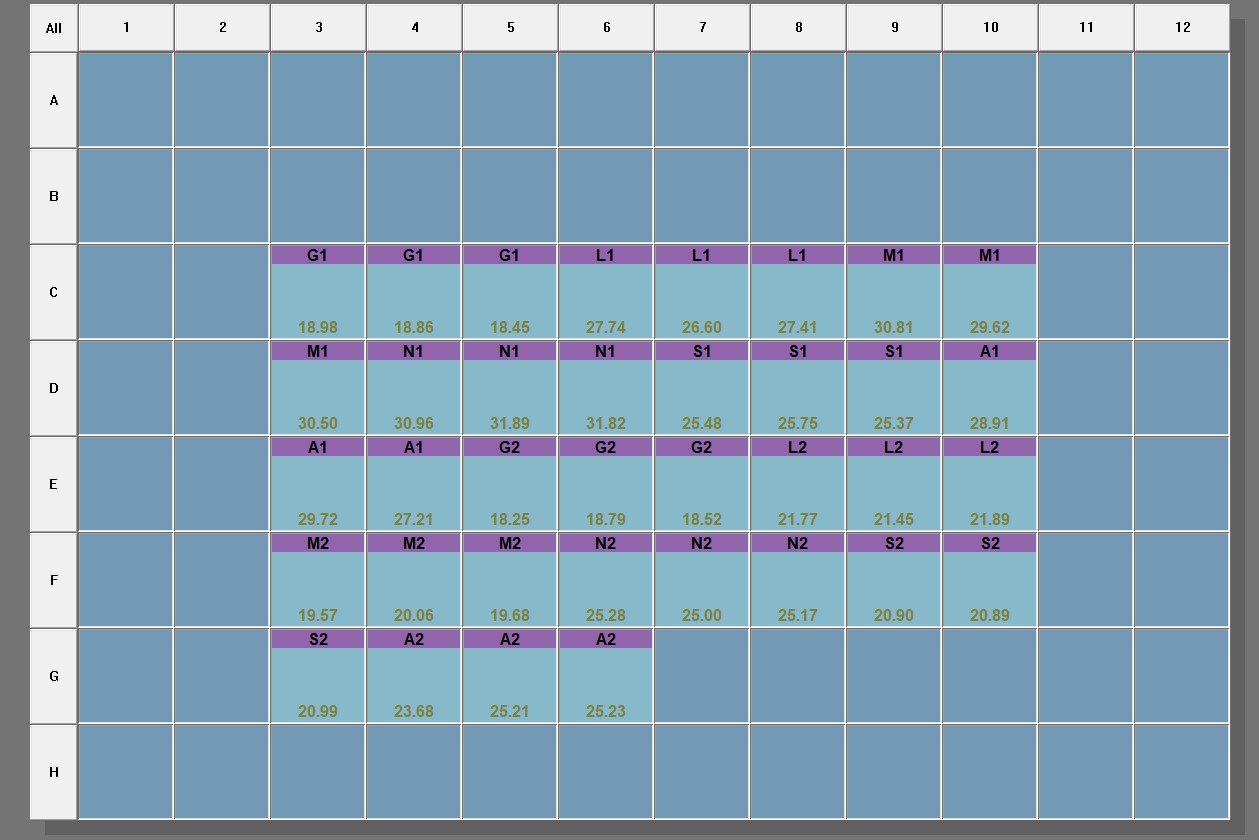

Supplement: Supplementary file 1 [file DataSheet_1.zip › excel+p/7-3.jpg]

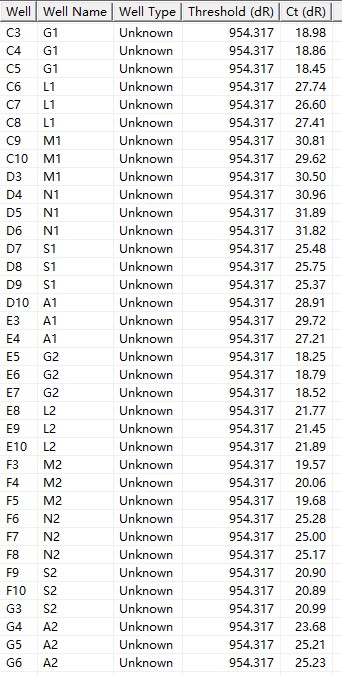

Supplement: Supplementary file 1 [file DataSheet_1.zip › excel+p/7-4.jpg]

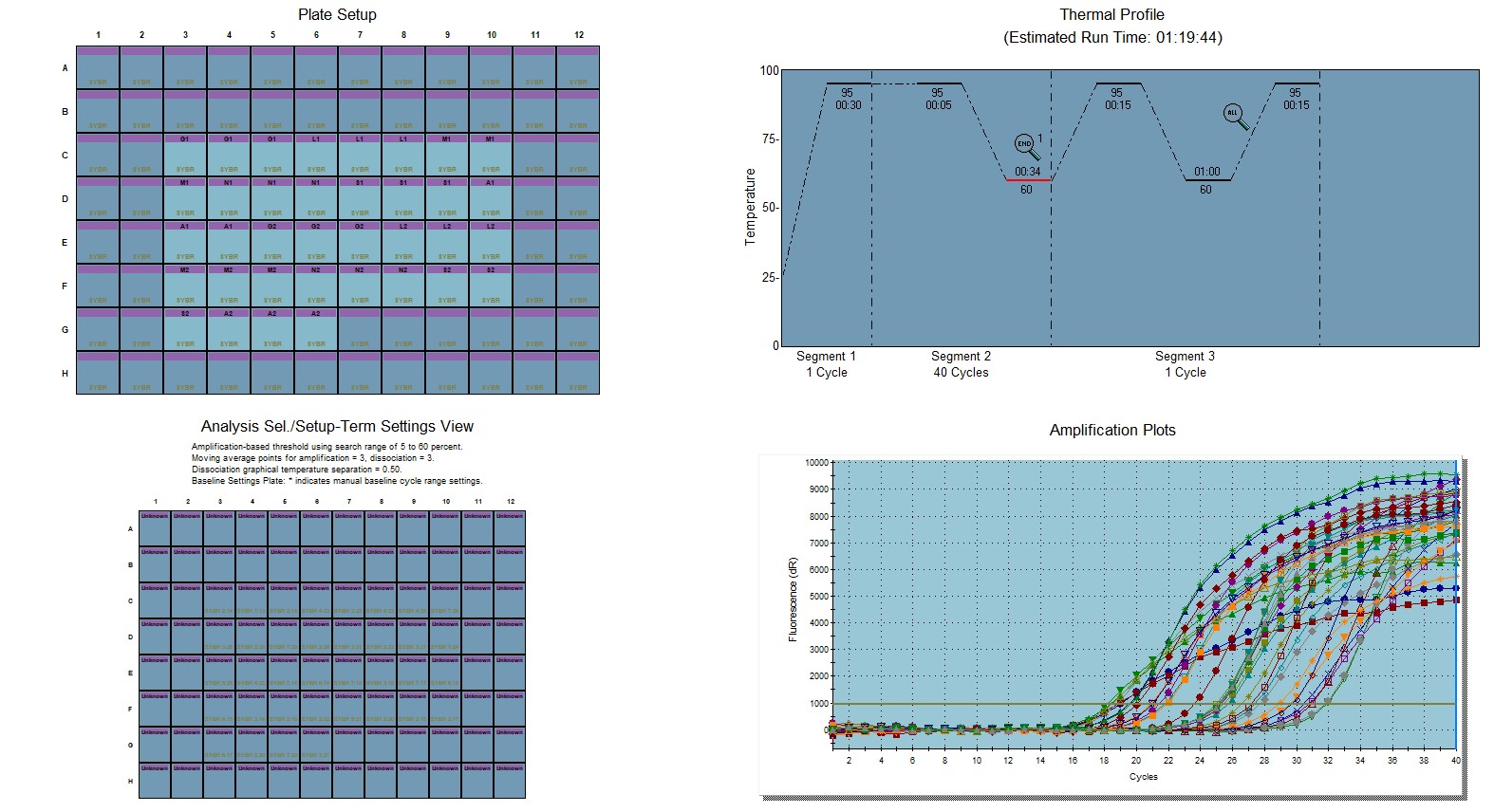

Supplement: Supplementary file 1 [file DataSheet_1.zip › excel+p/7.jpg]

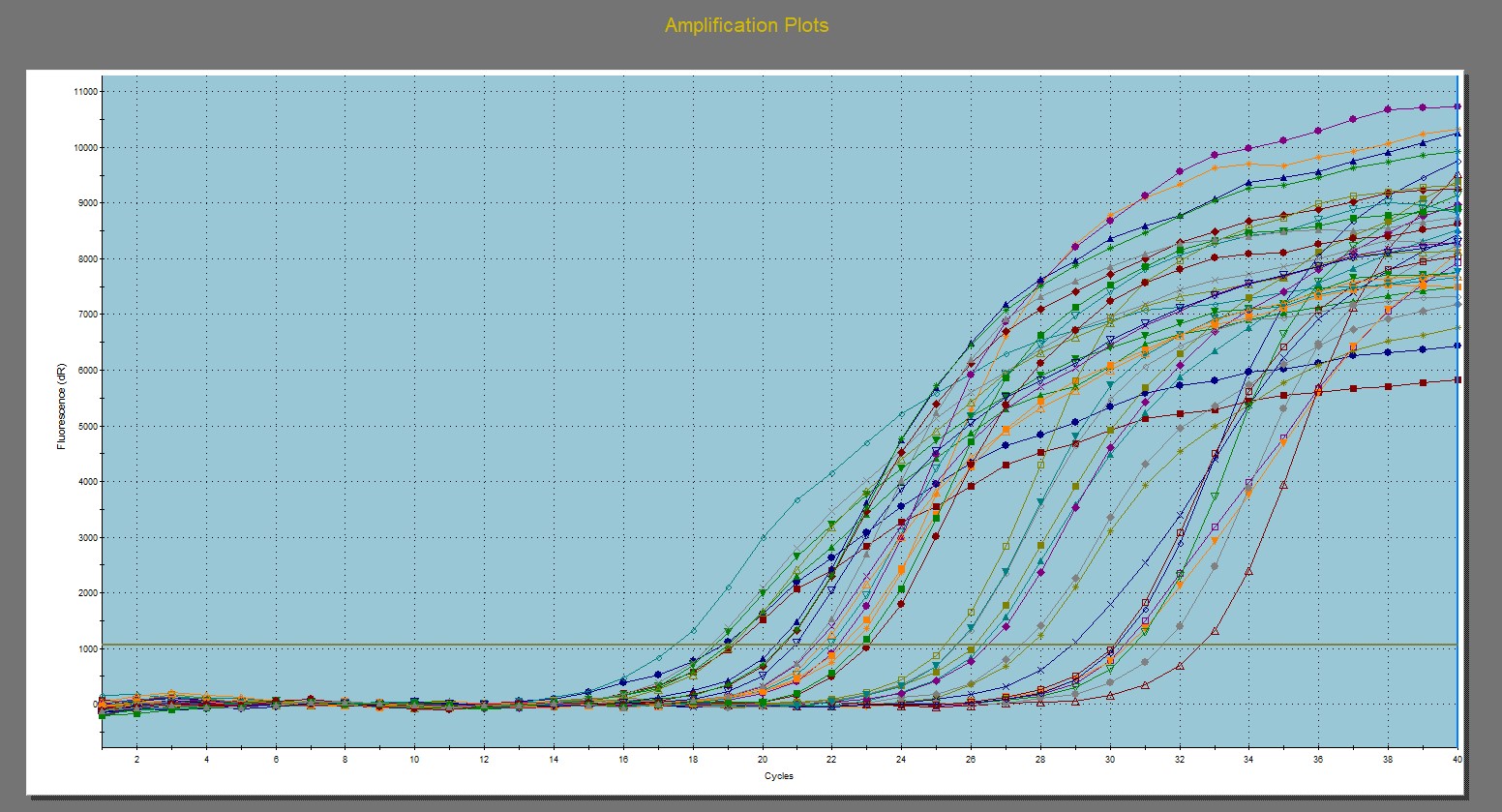

Supplement: Supplementary file 1 [file DataSheet_1.zip › excel+p/8-1.jpg]

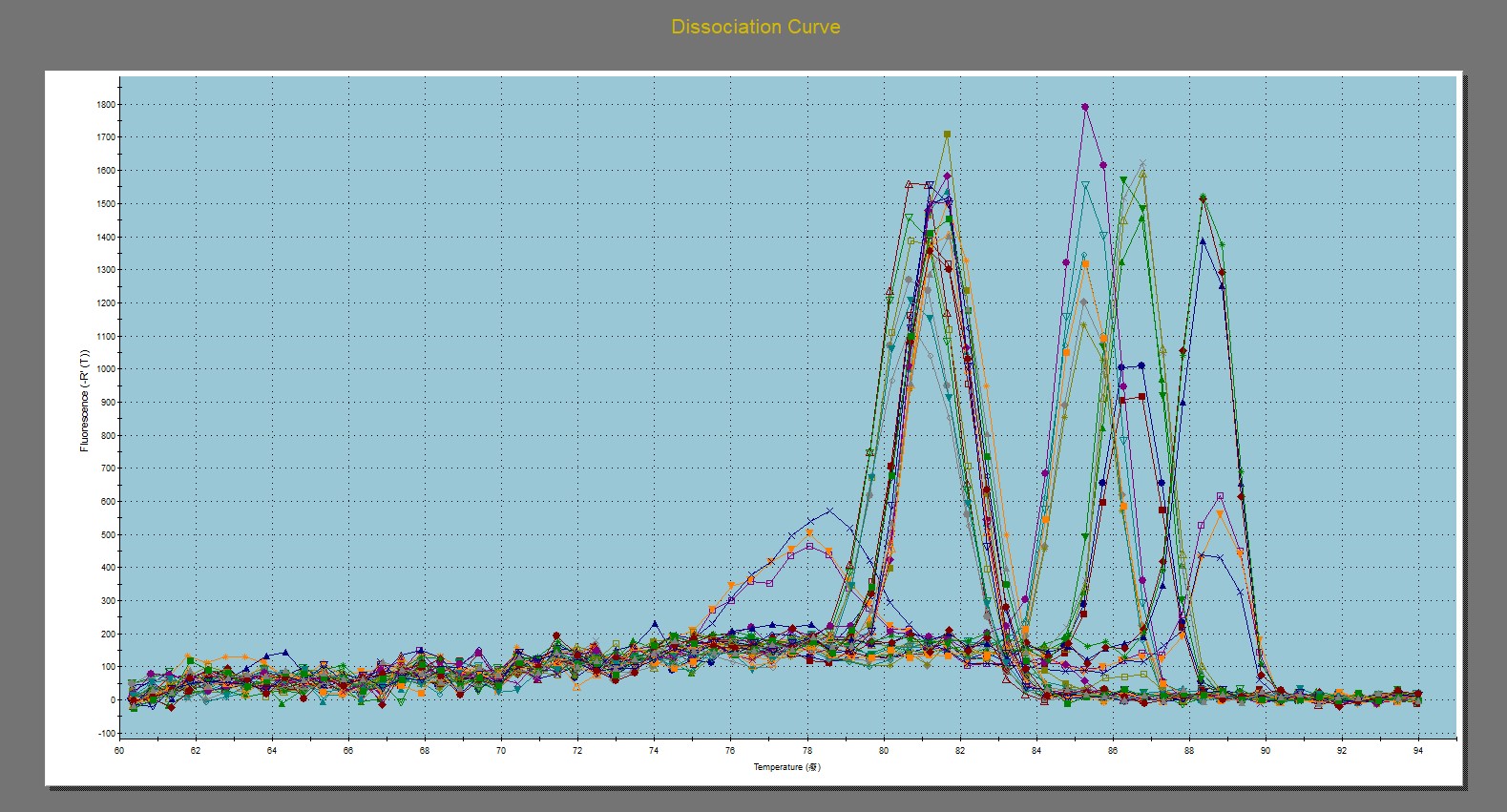

Supplement: Supplementary file 1 [file DataSheet_1.zip › excel+p/8-2.jpg]

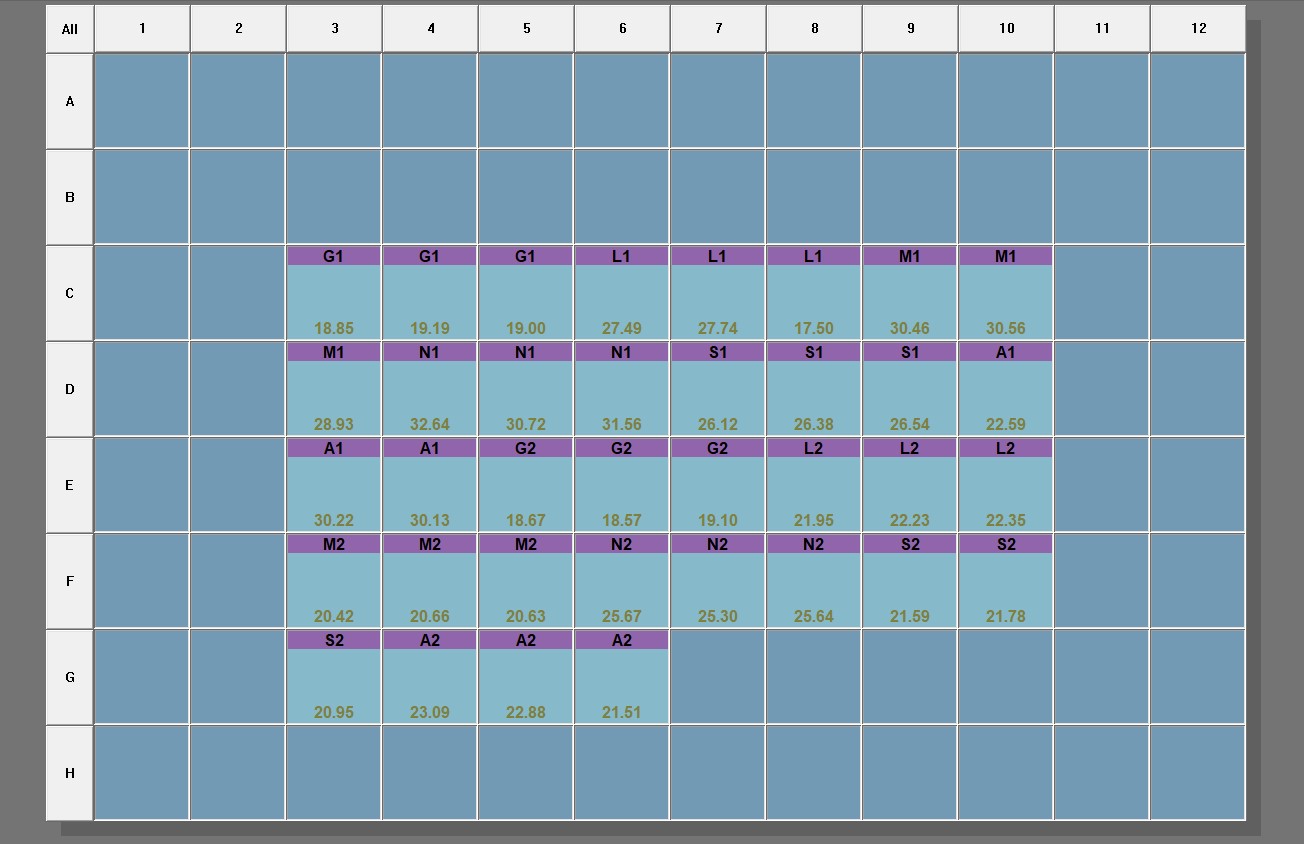

Supplement: Supplementary file 1 [file DataSheet_1.zip › excel+p/8-3.jpg]

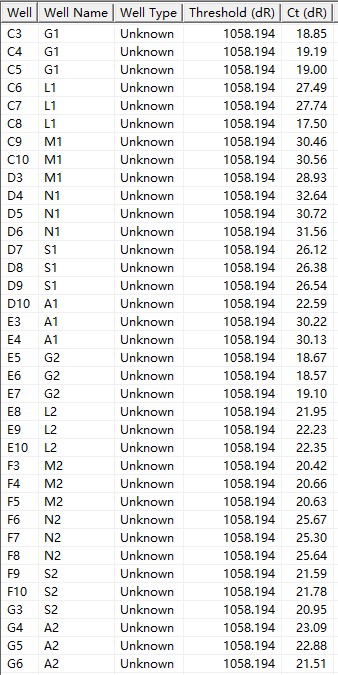

Supplement: Supplementary file 1 [file DataSheet_1.zip › excel+p/8-4.jpg]

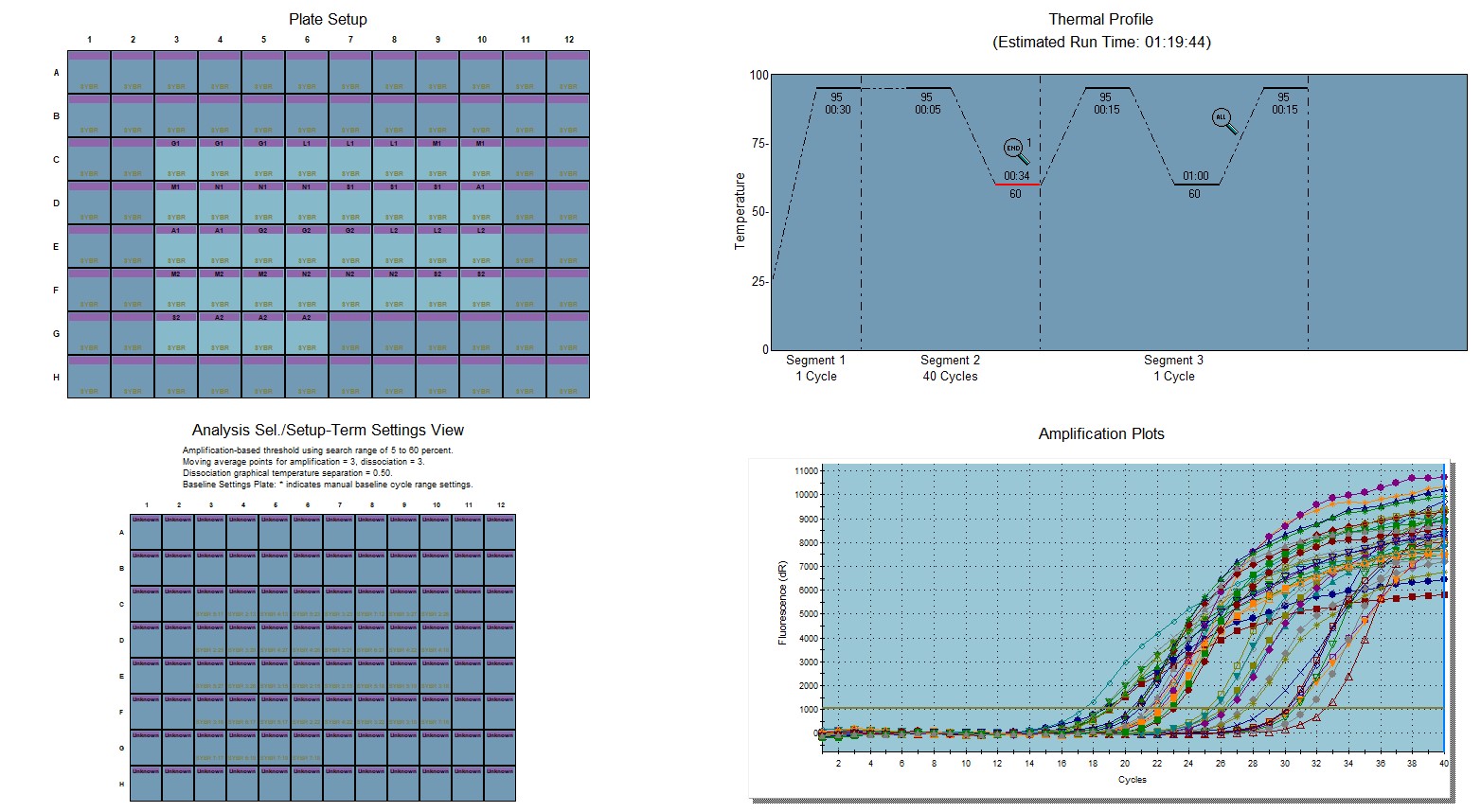

Supplement: Supplementary file 1 [file DataSheet_1.zip › excel+p/8.jpg]

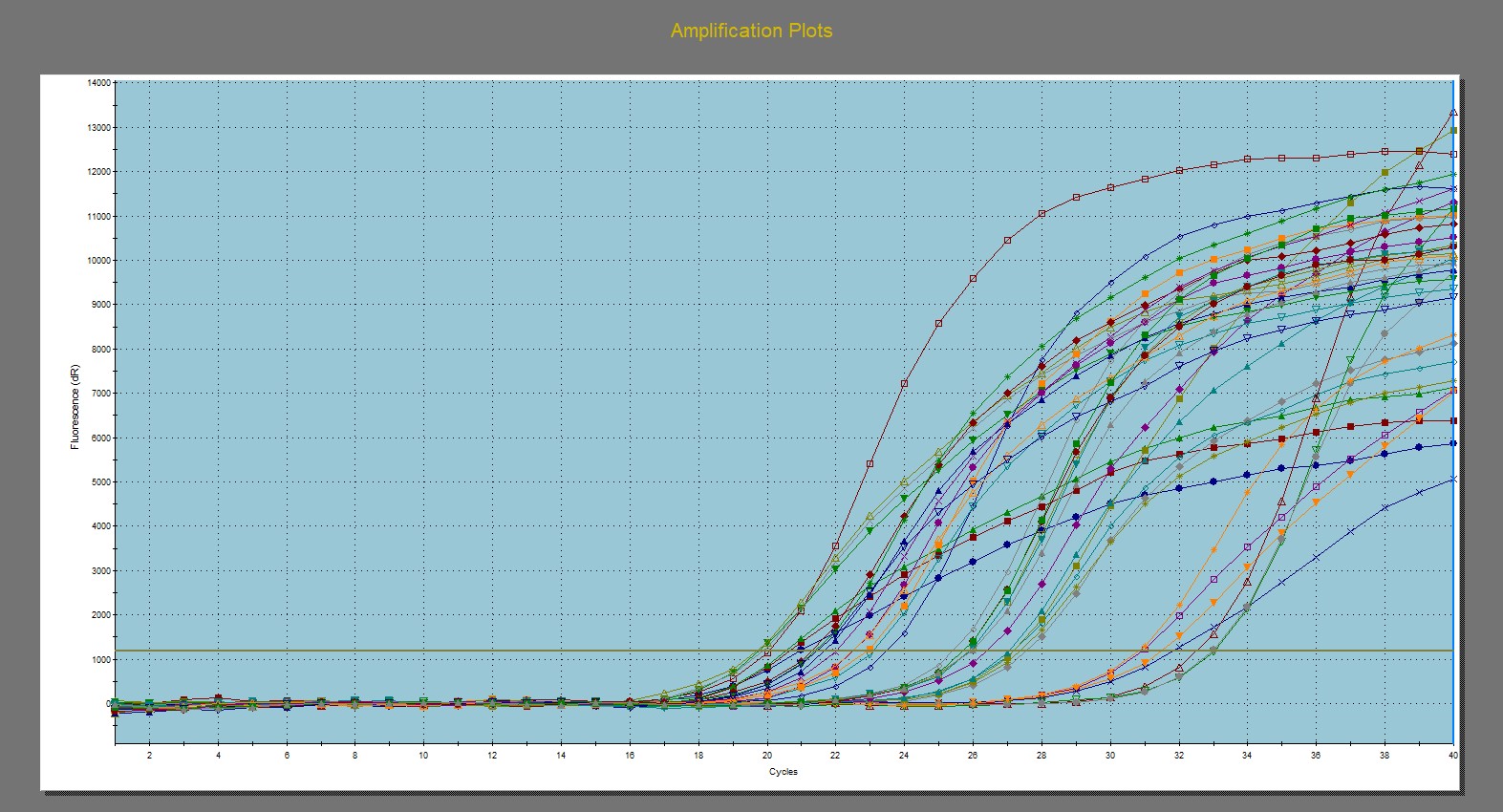

Supplement: Supplementary file 1 [file DataSheet_1.zip › excel+p/9-1.jpg]

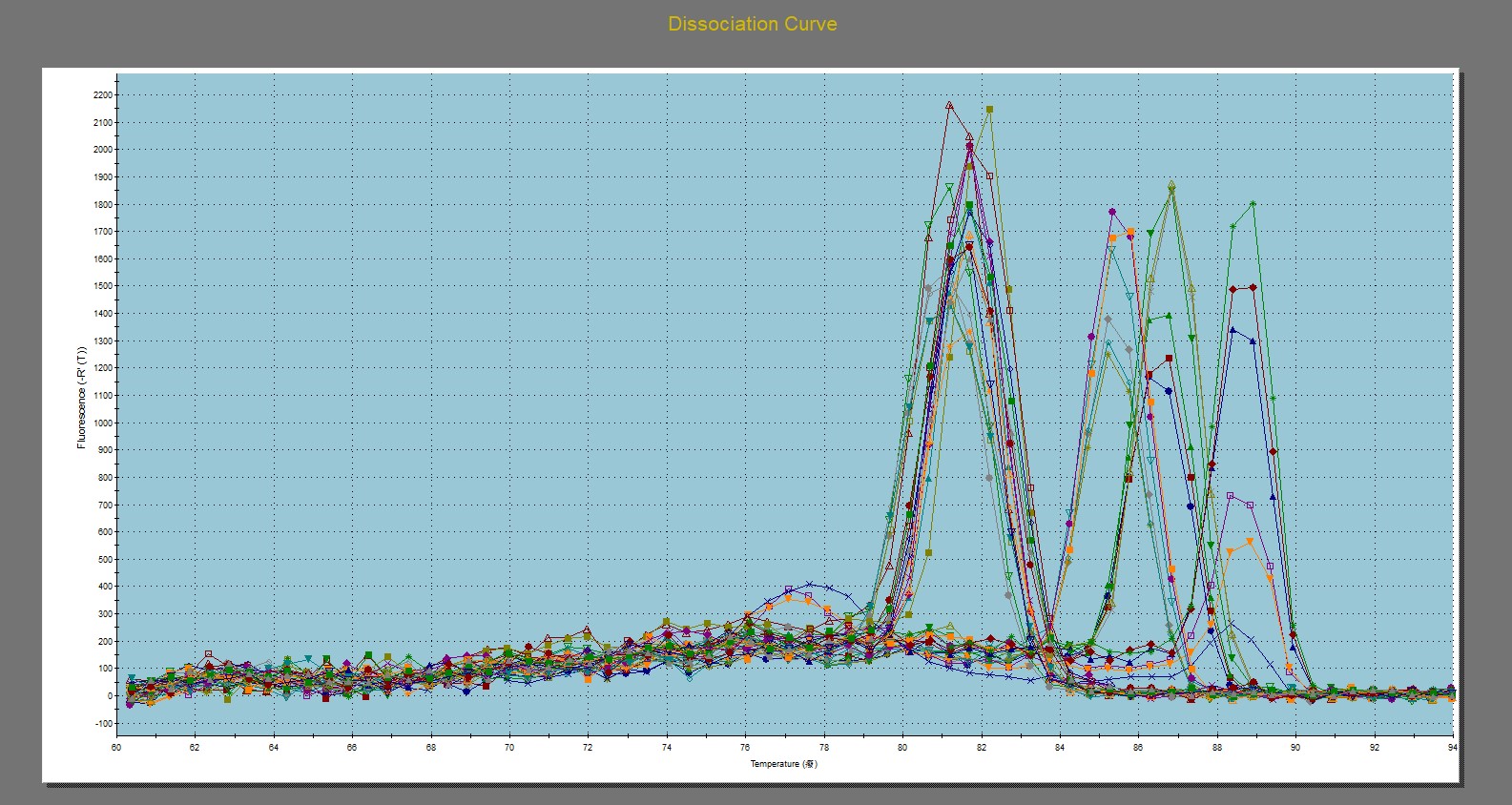

Supplement: Supplementary file 1 [file DataSheet_1.zip › excel+p/9-2.jpg]

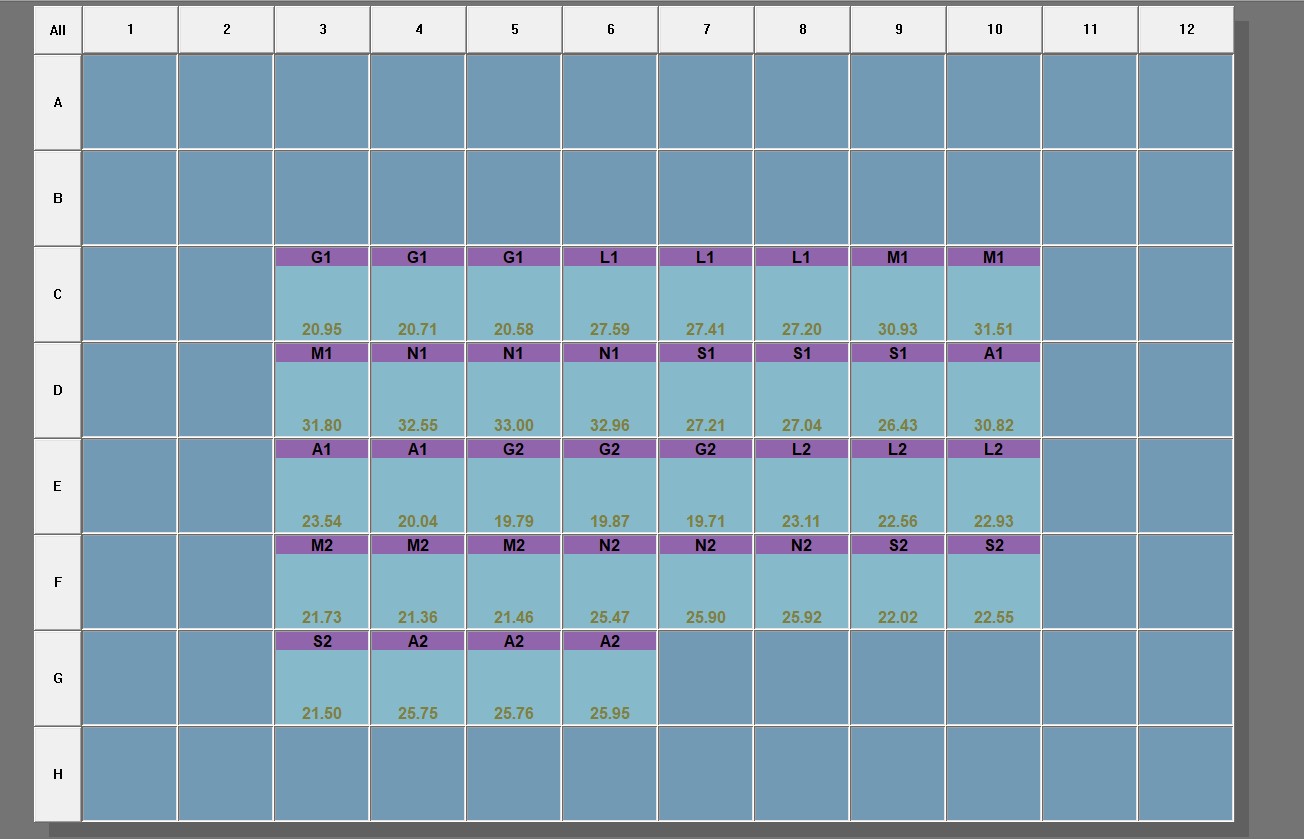

Supplement: Supplementary file 1 [file DataSheet_1.zip › excel+p/9-3.jpg]

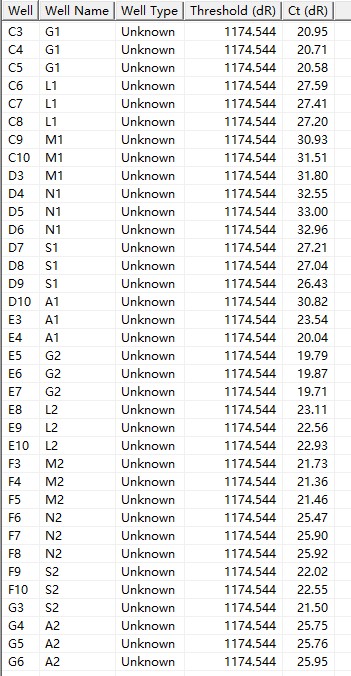

Supplement: Supplementary file 1 [file DataSheet_1.zip › excel+p/9-4.jpg]

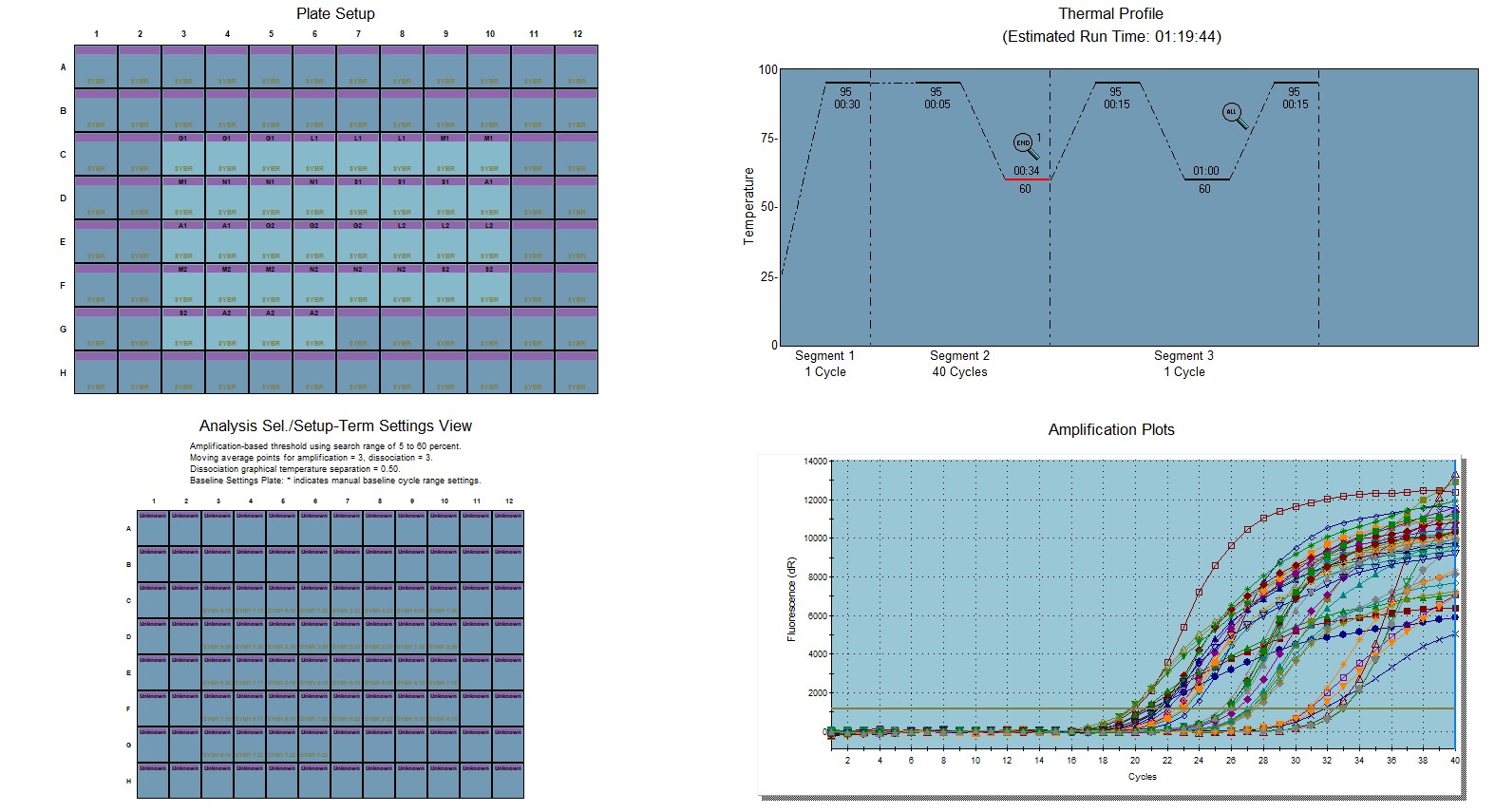

Supplement: Supplementary file 1 [file DataSheet_1.zip › excel+p/9.jpg]
